# Computerised interpretation of fetal heart rate during labour (INFANT): a randomised controlled trial

**DOI:** 10.1016/S0140-6736(17)30568-8

**Published:** 2017-04-29

**Authors:** The INFANT Collaborative Group, Peter Brocklehurst, David Field, Keith Greene, Ed Juszczak, Robert Keith, Sara Kenyon, Louise Linsell, Christopher Mabey, Mary Newburn, Rachel Plachcinski, Maria Quigley, Elizabeth Schroeder, Philip Steer

## Abstract

**Background:**

Continuous electronic fetal heart-rate monitoring is widely used during labour, and computerised interpretation could increase its usefulness. We aimed to establish whether the addition of decision-support software to assist in the interpretation of cardiotocographs affected the number of poor neonatal outcomes.

**Methods:**

In this unmasked randomised controlled trial, we recruited women in labour aged 16 years or older having continuous electronic fetal monitoring, with a singleton or twin pregnancy, and at 35 weeks' gestation or more at 24 maternity units in the UK and Ireland. They were randomly assigned (1:1) to decision support with the INFANT system or no decision support via a computer-generated stratified block randomisation schedule. The primary outcomes were poor neonatal outcome (intrapartum stillbirth or early neonatal death excluding lethal congenital anomalies, or neonatal encephalopathy, admission to the neonatal unit within 24 h for ≥48 h with evidence of feeding difficulties, respiratory illness, or encephalopathy with evidence of compromise at birth), and developmental assessment at age 2 years in a subset of surviving children. Analyses were done by intention to treat. This trial is completed and is registered with the ISRCTN Registry, number 98680152.

**Findings:**

Between Jan 6, 2010, and Aug 31, 2013, 47 062 women were randomly assigned (23 515 in the decision-support group and 23 547 in the no-decision-support group) and 46 042 were analysed (22 987 in the decision-support group and 23 055 in the no-decision-support group). We noted no difference in the incidence of poor neonatal outcome between the groups—172 (0·7%) babies in the decision-support group compared with 171 (0·7%) babies in the no-decision-support group (adjusted risk ratio 1·01, 95% CI 0·82–1·25). At 2 years, no significant differences were noted in terms of developmental assessment.

**Interpretation:**

Use of computerised interpretation of cardiotocographs in women who have continuous electronic fetal monitoring in labour does not improve clinical outcomes for mothers or babies.

**Funding:**

National Institute for Health Research.

## Introduction

Continuous electronic fetal heart rate monitoring in labour is widely used but its potential for improving neonatal outcomes has not been realised.^1^ The reasons for this are complex, but include difficulty interpreting the fetal heart rate trace correctly during labour.^2^, ^3^, ^4^ Computerised interpretation could be used to objectively detect abnormalities in fetal heart rate patterns during labour that are associated with asphyxia and bring them to the attention of birth attendants, who could then take action or expedite delivery and potentially prevent stillbirth or exposure to significant asphyxia.

Guardian (K2 Medical Systems, Plymouth, UK) is an electronic information capture system for managing information from labour monitoring.^5^ It displays the cardiotocograph on a computer screen alongside other clinical data either from external ultrasonographic transducers, or from fetal scalp electrodes (eg, partographs, maternal vital signs, details of maternal anaesthesia and analgesia) collected as part of routine clinical care. Guardian does not interpret any of the data gathered, but acts as an interface to collect and display data at the bedside, centrally on the labour ward, in consultants' offices, or remotely.

INFANT (K2 Medical Systems) is a decision-support software that was developed to run on the Guardian system. It analyses the quality of fetal heart signals and, if these signals are adequate, displays baseline heart rate; heart-rate variability; accelerations and type and timing of decelerations; the quality of the signal; and the contraction pattern.^6^, ^7^, ^8^ INFANT then makes an assessment of the overall pattern, which, if necessary, will result in a colour-coded alert (blue is the least severe, yellow is moderate severity, and red is the most severe; appendix). The decision-support software does not provide recommendations for any action that should be taken in response to abnormalities—such decisions are at the discretion of attending clinicians. In the UK's National Health Service (NHS), all clinicians supervising labour are expected to have been trained in the appropriate response to an abnormal cardiotocographic reading—eg, by completing computer-based training packages every 6–12 months, attending annual lectures, or attending regular cardiotocographic review meetings. However, training is not standardised, and individual competence is not assessed in most units.^9^

**Research in context**

**Evidence before the study**

The National Institute for Health Research searched the Cochrane library, PubMed, NHS Evidence, and the DARE database for articles published in the 5 years before it commissioned this trial in 2006. No studies of electronic decision support in interpretation of cardiotocography were identified. One previous trial of decision support in labour has been done, in which direct measurement of the fetal electrocardiograph—which requires the application of a fetal scalp electrode—was used. That trial recruited 7730 women and showed no evidence of a difference between the groups on the primary outcome of metabolic acidosis as measured in cord blood.

**Added value of this study**

This trial, INFANT, was the first to assess the use of decision support in the interpretation of the cardiotocographs in women undergoing continuous electronic fetal heart-rate monitoring. The aim was to address the effect on substantive outcomes of neonatal mortality and morbidity. The size of the INFANT trial provides sufficient power to detect small differences in the main outcome of perinatal mortality and serious morbidity, and outcomes such as metabolic acidosis. No differences were noted. Additionally, it had very high power to assess whether use of this decision-support system had any effect on the risk of operative delivery, and again we noted no evidence of any difference.

**Implications of all the available evidence**

The quality of care in labour is a major concern for women, their families, and the health professionals providing care for them. Technologies to improve intrapartum monitoring that identify early signs of fetal hypoxia during labour are needed so that clinicians can intervene and attempt to prevent poor neonatal outcomes. Our results show that the method of decision support we tested does not achieve this aim.

We did a randomised controlled trial to test the hypotheses that a substantial proportion of substandard care results from failure to correctly identify abnormal fetal heart rate patterns, that improved recognition of abnormality would reduce substandard care and poor outcomes, and that improved recognition of normality would decrease unnecessary intervention.

## Methods

### Study design and participants

We did a pragmatic, unblinded, randomised controlled trial in maternity units in the UK and Ireland. All 24 sites in the UK and Ireland that used Guardian at the time of the trial took part. Doctors and midwives were able to recruit participants. Eligible women were judged to require continuous electronic fetal heart rate monitoring by the local clinical team on the basis of their existing practice, had a singleton or twin pregnancy, were at 35 weeks' gestation or further along, had no known gross fetal abnormality, including any known fetal heart arrhythmia such as heart block, aged 16 years or older, and able to give consent to participate as judged by the attending clinicians. Continuous electronic fetal heart rate monitoring during labour is not routine in the UK. Clinical guidance for the NHS recommends that women assessed as having a low risk of complications should be offered intermittent auscultation during labour.^10^

Pregnant women attending recruiting hospitals were provided with written information about the trial during pregnancy and in labour. For women who met the eligibility criteria, written informed consent was sought by means of a dated signature from the woman and from the person who obtained informed consent. Research ethics committee approval for the study was granted by the National Research Ethics Service—Northern and Yorkshire Research Ethics Committee (09/H0903/31). The study protocol has been published.^11^

### Randomisation and masking

The Guardian system was used to confirm that all necessary eligibility criteria were met and then to randomly allocate women (1:1) to cardiotocography with or without decision support. The allocations were computer generated in Stata (version 10.1) by the trial statistician, who used stratified block randomisation, in which variable block sizes were used to balance between the two trial arms by whether the pregnancy was a singleton (block sizes 12, 14, 16, 18, 20, 22, and 24, allocated in proportion to the elements of Pascal's triangle—1:6:15:20:15:6:1) or twin (block sizes 2, 4, and 6, allocated in proportion to the elements of Pascal's triangle 1:2:1), and within each participating centre. The trial was not blinded, which allowed indirect measurement of any changes in clinician behaviour, such as how much time the attending midwife spent with the woman on the basis of the knowledge that the decision-support system was active or not.

### Procedures

Clinicians in participating centres were trained in the use of the decision-support software by staff from the trial office. A training team at each site was responsible for cascading training among the local clinicians. Women in the trial were managed according to standard procedures. No additional training was provided to clinical staff in how to respond to fetal heart rate abnormalities.

Labour data and outcomes were stored contemporaneously on the Guardian system, which were then sent electronically to the trial office. Data were extracted from notes of babies admitted to the neonatal unit and for all neonatal deaths. All children surviving were flagged at the NHS Information Centre for those born in England and NHS Greater Glasgow and Clyde Safe Haven for those born in Scotland, which meant that all deaths occurring after discharge in these countries could be identified. Ireland does not have a similar system for monitoring deaths, so Irish data had to be excluded from the denominator for these calculations. A sample of surviving children were followed up to age 2 years via a parent-completed questionnaire to assess the child's health, development, and wellbeing (appendix).

All babies with an adverse outcome potentially associated with intrapartum asphyxia (ie, the trial primary outcome plus cord artery pH <7·05 with base deficit 12 mmol/L or more) and all neonatal deaths and intrapartum stillbirths had their care in labour assessed by review of de-identified case notes by a panel comprising a senior obstetrician, neonatologist, and midwife, to see if care was suboptimal—ie, if it was possible or probable that different management would have prevented the adverse outcome.^9^, ^12^

### Outcomes

The trial had two primary outcomes. The first was a binary (present or not present) composite of poor neonatal outcome, including deaths (intrapartum stillbirths plus neonatal deaths up to 28 days after birth, except for congenital anomalies deaths) and significant morbidity (moderate or severe neonatal encephalopathy, defined as the use of whole-body cooling or admission to the neonatal unit within 48 h of birth for 48 h or more with evidence of feeding difficulties or respiratory illness, with evidence of compromise at birth suggesting mild asphyxia or mild encephalopathy, or both). The second was a continuous outcome of developmental progress measured by the Parent Report of Children's Abilities—Revised (PARCA-R) composite score^13^, ^14^ at age 2 years for a subset of children.

Infant secondary outcomes, all of which are binary unless specified, were intrapartum stillbirth (excluding deaths from congenital anomalies); neonatal deaths up to 28 days after birth (excluding deaths from congenital anomalies); moderate or severe encephalopathy; admissions to the neonatal unit within 48 h of birth for 48 h or more with evidence of feeding difficulties or respiratory illness (when there was evidence of compromise at birth suggesting mild asphyxia or mild encephalopathy, or both); admission to a higher level of care; an Apgar score of less than 4 at 5 min; distribution of cord blood gas data for cord artery pH; metabolic acidosis (cord artery pH <7·05 and base deficit of 12 mmol/L or more); resuscitation interventions (categorical); seizures; destination immediately after birth (categorical); length of hospital stay (continuous); health and development outcomes at 24 months (continuous); score on the non-verbal cognition scale (continuous), vocabulary sub-scale (continuous), and sentence complexity sub-scale (continuous) of PARCA-R; deaths to 24 months; major disability and non-major disability at 2 years; and cerebral palsy. Maternal secondary outcomes were mode of delivery (categorical); operative intervention (caesarean section and instrumental delivery) for fetal indications, failure to progress, a combination of fetal indications and failure to progress, or any other reason; grade or urgency of caesarean section^15^ (categorical); episiotomy; any episode of fetal blood sampling; length of first stage, length of second stage, and total length of labour from trial entry (continuous); destination immediately after birth (categorical); and admission to a higher level of care.

Because trial allocation was not blinded, it was important to measure any change in clinical care that could result from clinicians being aware of whether the decision-support system was in operation. We measured the total number of cardiotocographic abnormalities and the proportion of women with cardiotocographic abnormalities in each arm; the time taken between last red alert and delivery (for these first three outcomes, we retrospectively used the decision-support software after the trial was over to analyse the cardiotocographic trace, and used these data to determine when the alert would have occurred); the number of routine measurements recorded during labour, including the number of vaginal examinations, use of epidural analgesia, use of labour augmentation, and presence of meconium; and the number of thumb entries (similar to a signature in paper notes) per hour from time of trial entry to first yellow level of concern or until the cervix was fully dilated (as a proxy measure to assess presence of a health professional in the delivery room during the labour).

### Statistical analysis

A sample size of 46 000 births was needed.^11^ We postulated an incidence of the primary outcome of three per 1000 births by summing the previous reported rates of intrapartum stillbirth, neonatal death, moderate and severe encephalopathy, and mild encephalopathy (reliable data for significant asphyxial morbidity were not available and so could only be estimated).^16^, ^17^, ^18^ The effect size that could be detected with 46 000 women (23 000 in each group), assuming a 5% level of significance and 90% power, was a 50% reduction in poor neonatal outcomes from three to 1·5 per 1000. In a study of preterm infants,^14^ the mean PARCA-R composite score at 2 years was 80 (SD 33) and the mean Mental Development Index (Bayley Scales of Infant Development II) was around half an SD below the mean of 100. Assuming that a healthy group of term infants would have a PARCA-R composite score half an SD higher than this sample of preterm infants, we estimated a mean 2 year score of 96 (SD 33). A follow-up sample of size 7000 (3500 per arm) had more than 90% power to detect a difference of 3 points in the PARCA-R component score with a two-sided 5% significance level. The incidence of severe metabolic acidosis (cord artery pH <7·05) is ten per 1000.^19^, ^20^, ^21^, ^22^ A sample of 46 000 women enabled us to detect a 28% relative risk reduction in this incidence with more than 80% power, assuming a 5% level of significance, in babies in whom cord artery pH was measured.

During the early part of the trial, and with advice from the data-monitoring committee, the primary outcome definition was refined to ensure that it captured babies who were likely to have experienced hypoxia during labour. The original component of the primary outcome—admission to neonatal unit within 48 h of birth for 48 h or more with evidence of feeding difficulties, respiratory illness, or encephalopathy—resulted in the inclusion of many babies with a range of disorders, many of which were unlikely to be related to hypoxia. Each case fulfilling this component of the primary outcome was reviewed by an independent panel of neonatologists (who were blinded to allocation) to ascribe it as fulfilling the revised definition or not (appendix).

The trial steering committee approved the statistical analysis plan before the analysis (appendix). For the main comparative analysis, participants were analysed in the groups into which they were randomly allocated, irrespective of allocation received. All women and babies with available data were included, except for women for whom a valid signed consent form could not be located or women who withdrew consent. The numbers and percentages of babies in whom the primary outcomes were noted are for each group, and the risk ratios plus 95% CIs were calculated. Risk ratios were estimated with generalised linear models with a binomial distribution and a log link (or a Poisson distribution with a log link if convergence could not be achieved). Hazard ratios were estimated with Cox regression and rate ratios with Poisson regression. We adjusted for the stratification factors used in the randomisation (centre and singleton or twin pregnancy), and used robust variance estimators in all models to account for the correlation in outcomes between twins and siblings delivered in a subsequent pregnancy during the trial period.^11^ The mean (SD) PARCA-R composite score was calculated for each group, and the mean difference between groups plus 95% CI were calculated and compared with a Gaussian model with identity link. For secondary outcomes including the components of the primary outcome, a 1% level of significance was employed.

We did prespecified subgroup analyses with the statistical test for interaction for singletons versus twins, suspected fetal growth restriction at labour onset versus no growth restriction, body-mass index group, and centre. These analyses were done for the trial primary outcomes, all neonatal outcomes, instrumental vaginal deliveries, and caesarean section. Additionally, we did a subgroup analysis of all process outcomes by centre. Major disability at 2 years was classified in terms of neuromotor function, seizures, auditory function, communication, visual function, cognitive function, and other physical disability.^23^, ^24^

We used Stata/SE for Windows (version 13.1) for all analyses. The trial was overseen by an independent trial steering committee and an independent data-monitoring committee. The data-monitoring committee used the Haybittle-Peto approach^25^ for interim analyses, with three SEs as the cutoff for consideration of early cessation, preserving the type-1 error across the trial.

The trial is registered with ISRCTN, number 98680152.

### Role of the funding source

The funder of the study had no role in the trial design; data collection, analysis, or interpretation; or writing of the report. The corresponding author had full access to all the data in the study and had final responsibility for the decision to submit for publication.

## Results

Between Jan 6, 2010, and Aug 31, 2013, 47 062 women were recruited to the INFANT trial (appendix). 1020 women (2·2%) were excluded from the analysis of the primary outcome (figure; appendix), mostly because of missing or incomplete consent forms. Data at the time of birth were available for 100% of women and babies eligible to be analysed. Follow-up data at 2 years were available for 7066 of those contacted; data were sufficiently complete for 6707 children.

Baseline characteristics were similar between the two groups (table 1). Median maternal age was 29 years (IQR 25–33). Around 60% of women were having their first baby, and most women had a gestational age between 38 and 41 completed weeks (table 1). Very few women had a previous stillbirth (1%) and around 6% had previously had a previous caesarean section (table 1). Almost 60% of women had their labour induced.

The incidence of the primary outcome—poor neonatal outcome—did not differ significantly between the groups (table 2). 172 (0·7%) of 23 263 babies had a poor outcome in the decision-support group compared with 171 (0·7%) of 23 351 babies in the no-decision-support group (adjusted risk ratio [RR] 1·01, 95% CI 0·82–1·25; table 2). Similarly, we noted no evidence of a difference in any component of the composite primary outcome between the groups (table 2). A prespecified sensitivity analysis, in which we used a different cutoff for defining compromise at birth (a score of 7 or greater indicating very severe compromise rather than a score of 3 or greater on a scale from 0 to 14), made no difference to the interpretation of the measure of effect for the primary outcome (adjusted RR 0·97, 95% CI 0·58–1·63; appendix). We noted no evidence of any differences in any of the trial's secondary outcomes for the baby (table 2), including Apgar scores, admission to the neonatal unit, metabolic acidosis of cord blood samples, the need for neonatal resuscitation, or duration of hospital stay.

Just over half of all births were spontaneous vaginal births and the frequency did not differ significantly between the two groups (adjusted RR 0·99, 99% CI 0·97–1·01; table 2). More women underwent fetal blood sampling in the decision-support group than in the no-decision-support group (2366 [10·3%] *vs* 2187 [9·5%]; adjusted RR 1·08, 99% CI 1·01–1·16). No other significant differences were noted between the two groups from trial entry to birth in terms of clinical outcomes (table 2).

For babies with an adverse outcome and cord metabolic acidosis who underwent expert review, the overall proportion of babies judged to have received suboptimal care likely to have affected the outcome was 38%—14 of 35 babies in the decision-support group and 13 of 36 babies in the control group—which is similar to that reported previously.^26^ We could not investigate whether in all cases not reviewed appropriate action was taken in response to recognised abnormality.

In women with any level of concern as measured by INFANT (table 3), blue levels of concern were most frequent (median seven alerts during labour—roughly 1·1 per h), followed by yellow alerts (median two alerts per labour), and then red alerts (median one per labour). A lower rate of yellow levels of concern was noted in the decision-support group compared with the no-decision-support group (adjusted rate ratio 0·87, 99% CI 0·84–0·89; table 3). Frequency of blue and red alerts did not differ significantly between groups (table 3).

Although there was a worry that women in the decision-support group would be left alone more frequently during labour than those in the no-decision-support group, the frequency of thumbprint entries on the Guardian did not differ significantly between groups (4·22 per h *vs* 4·21; adjusted rate ratio 0·99, 99% CI 0·95–1·03; table 3).

Time from the last red level of concern to birth was similar in both groups (median 58 min; table 3). In a subgroup of 473 readable traces from a sample of 500 taken as a similar number of consecutive cases from each contributing centre, the last red level of concern was judged (by expert investigator, PS) to be a valid fetal concern for 276 (58%) traces. Maternal heart rate triggered the red level of concern in 128 (27%) cases, misclassified accelerations in 36 (8%) cases, and other reasons in 33 (7%) cases.

Families were contacted when their surviving child or children born reached age 2 years. Nearly 7000 families returned a questionnaire. The characteristics of the mothers who responded differed significantly from those of the entire trial cohort and from those of mothers who did not respond (appendix). Compared with the entire trial cohort, responders were more likely to be white, to have given birth at a later gestational age, and to have been having their first baby (appendix).

Of the 7066 infants for whom a questionnaire was returned, data could be analysed for 6707 (95%). We noted no significant differences between the two groups for any of the 2 year outcomes, including the primary outcome, PARCA-R score (table 4). Nearly 6% of children for whom data were available had a major disability. The classification of disability^23^, ^24^ used meant that large numbers of children were assigned a major disability as a result of having poor growth (between 2·8% and 3·0% of all children) and cognitive difficulties (between 1·2% and 1·5% of all children). Other major disabilities such as physical disability, blindness, and deafness were all very uncommon (appendix).

We noted no evidence that the decision-support software performed significantly differently between any of the pre-specified subgroups for either the primary outcome or a range of secondary outcomes (appendix). Furthermore, no differences were noted in the distribution of cord blood pH measurements (appendix). The number of alerts differed significantly by centre, but no other significant differences were noted by centre (appendix).

## Discussion

In this trial of more than 46 000 women, we found no evidence that the use of decision-support software in conjunction with cardiotocography reduced the likelihood of poor neonatal outcomes compared with cardiotocography alone.

In another randomised trial, which also recruited in the UK, the use of decision support was assessed in women monitored during labour with fetal electrocardiographic monitoring.^27^ This study also showed no evidence that decision support improved the primary outcome of cord blood metabolic acidosis in 7730 women.^27^ The results of one small trial^28^ of 220 women in Bulgaria have suggested that decision support is associated with benefits with respect to cord blood metabolic acidosis. In the UK continuous electronic fetal heart-rate monitoring is not routine, making generalisability to settings in which it is routine less certain.

The strengths of this study are its contemporaneous data collection and size. Potential weaknesses include the challenges of use of a composite primary outcome measure, the potential for staff to learn from exposure to the decision-support arm of the trial, resulting in improved outcomes in the control arm, and the issue of accounting for multiple comparisons.

Use of a composite primary outcome might not always be helpful if different components of the outcome respond differently to the intervention.^29^ We initially hypothesised that the components of the outcome would have similar incidences, with each component likely to contribute around a third to the composite. Estimates of the incidence of these components for eligible women were difficult to find before the trial began.^11^ The perinatal mortality in our study (13 per 46 614 babies [0·3 per 1000]) was lower than the previous estimate (1·05 per 1000), and the incidence of neonatal encephalopathy requiring cooling was also lower than previous estimates (0·8 per 1000 *vs* 1·3 per 1000).^11^ However, the incidence of prolonged admission to neonatal units with evidence of compromise at birth, for which we had no good data when planning the trial, occurred more frequently (291 per 46 614 [six per 1000]) and contributed substantially more to the higher-than-anticipated overall primary event rate of seven per 1000 compared with our estimated three per 1000. This frequency afforded us power to detect smaller differences in the composite outcome than we had originally planned.

The potential weakness of staff learning from exposure to the decision-support system was identified when planning the trial. We acknowledged that passive learning from the decision-support system would be possible. However, part of our previous hypothesis was that, although some poor cardiotocographic interpretation is due to a lack of training, some clinicians have poor intrinsic pattern-recognition abilities, which, by definition, would not be affected by training, and the performance of such clinicians would be particularly improved by assistance from automatic interpretation. We collected a range of process outcomes to measure the impact on clinician behaviour during the trial, and these data suggested some evidence for behaviour change in the decision-support arm: fetal blood sampling was more frequent and the incidence of repeated yellow alerts lower than in the control group. Perhaps different action was taken in response to the alerts in the decision-support arm of the trial—eg, clinicians might have reduced the dose of an oxytocin infusion in women having their labour augmented if the infusion was leading to very frequent contractions. Such actions could have prevented further yellow alerts, leading to a decrease in this group, although we do not have any direct evidence for this scenario. Even if this effect did occur, it did not result in any significant change in clinical outcomes. Although the median time from last level of red concern to birth might seem lengthy (58 min), some red levels of concern did not prompt immediate delivery—eg, the cardiotocographic monitor picking up the maternal heart rate.

We accounted for multiple comparisons in the trial by using 99% CIs for all secondary outcomes. Significant findings in secondary outcomes require careful interpretation irrespective of the level of significance, and factors such as the strength of the finding and plausibility need to be taken into account.^30^ In this trial, the only two significant findings relate to behaviour change in clinicians favouring decision support. These findings were in the expected direction of effect and are mutually supportive, suggestive of a real effect.

Detection of abnormalities in the fetal heart rate can improve outcome only if caregivers respond appropriately to the alerts. A review of all severe adverse outcomes in the trial showed no evidence of differences in suboptimal care between the two groups. Therefore our hypothesis that substandard care is largely related to failure to identify pathological fetal heart-rate patterns is not supported. Most adverse outcomes associated with preventable substandard care seemed to involve failure to take appropriate management decisions once the cardiotocographic abnormality had been recognised. This aspect will be reported in detail in a follow-up paper. Our hypothesis that unnecessary intervention would be reduced was also not supported.

The decision-support software used in this trial identifies fetal heart-rate abnormalities.^6^, ^7^, ^8^ However, the alerts do not take into account other information about the labour, such as duration of labour, the rate of labour progress, and presence of meconium, all of which could modify the way a clinician interprets the fetal heart rate and acts on this information. Further development of decision-support software could improve the quality of feedback that the system provides to clinicians to make a difference to outcomes. In view of the importance for parents, clinicians, and health services of the consequences of intrapartum hypoxia, identification of signs of early compromise during labour so that timely intervention can be used to reduce poor outcomes is an urgent unmet need.

Correspondence to: Prof Peter Brocklehurst, Birmingham Clinical Trials Unit, Institute of Applied Health Research, College of Medical and Dental Sciences, University of Birmingham, Edgbaston, Birmingham B15 2TT, UK **p.brocklehurst@bham.ac.uk**

## Figures and Tables

**Figure:**
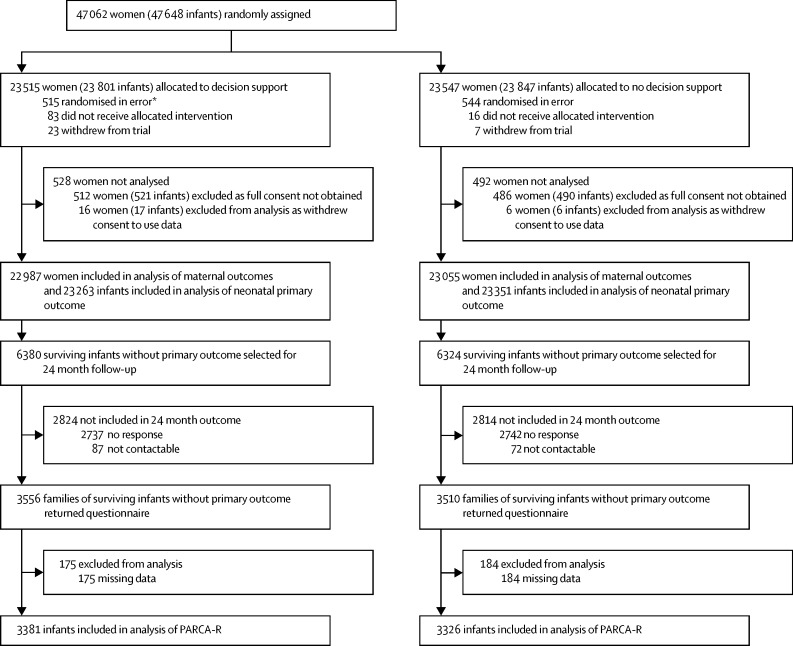
Trial profile The 46 042 women included in the analysis includes 448 women with two singleton birth episodes and six women with one singleton and one twin birth episode in the study period. The allocation received for the subsequent delivery was independent of the first allocation received. 30 of 21 509† infants in the decision-support group and 37 of 21 599† infants in the no-decision-support group died before 24 months. Parent Report of Children's Abilities—Revised (PARCA-R). *One woman who withdrew (with consent to use of data) was also randomly assigned in error. †Data from the Ireland not included in the numerator (n=1 decision support) or denominator (n=1754 in decision support and n=1752 in no decision support) because data for deaths after discharge were not available; deaths in the flow chart include stillbirths (n=1 in decision support and n=2 in no decision support).

**Table 1:** Maternal characteristics at trial entry

		Decision support (n=22 987)*	No decision support (n=23 055)*
Median age, years		29 (25–33)	29 (25–33)
Ethnic group†			
	White	17 234 (83·3%)	17 213 (83·0%)
	Indian	743 (3·6%)	724 (3·5%)
	Pakistani	736 (3·6%)	802 (3·9%)
	Bangladeshi	98 (0·5%)	113 (0·5%)
	Black Caribbean	116 (0·6%)	135 (0·7%)
	Black African	461 (2·2%)	505 (2·4%)
	Any other ethnic group	1296 (6·3%)	1249 (6·0%)
	Unknown	2303	2314
Twin pregnancy		276 (1·2%)	296 (1·3%)
Gestational age, completed weeks			
	Median	40 (39–41)	40 (39–41)
	<35 weeks	4 (<1%)	6 (<1%)
	35 weeks to 37 weeks, 6 days	2529 (11·0%)	2522 (10·9%)
	38 weeks to 39 weeks, 6 days	7322 (31·9%)	7266 (31·5%)
	40 weeks to 41 weeks, 6 days	11 688 (50·9%)	11 795 (51·2%)
	≥42 weeks	1437 (6·3%)	1457 (6·3%)
Body-mass index (at booking visit)			
	Median	25 (22–30)	25 (22–30)
	<18·5	379 (2·5%)	384 (2·6%)
	18·5– 24·9	6302 (42·1%)	6225 (41·6%)
	25·0–29·9	4531 (30·2%)	4560 (30·5%)
	30·0–34·9	2178 (14·5%)	2237 (14·9%)
	35·0–39·9	1024 (6·8%)	1025 (6·8%)
	≥40·0	565 (3·8%)	544 (3·6%)
	Unknown	8008	8080
Smoking (at booking visit)			
	Yes	2448 (14·3%)	2536 (14·7%)
	No	14 724 (85·7%)	14 722 (85·3%)
	Unknown	5815	5797
Parity			
	Nulliparous	13 736 (59·8%)	13 650 (59·2%)
	Parous	9247 (40·2%)	9390 (40·8%)
Obstetric history			
	Stillbirth	273 (1·2%)	223 (1·0%)
	Elective caesarean section	208 (0·9%)	253 (1·1%)
	Emergency caesarean section	1240 (5·4%)	1224 (5·3%)
	Neonatal death	80 (0·4%)	95 (0·4%)
Cervical dilatation at trial entry (cm)			
	Median	4 (2–6)	4 (2–5)
	Unknown	16 184	16 339
Fetal growth restriction suspected at labour onset		859 (3·7%)	914 (4·0%)
Labour induction			
	Induced	13 516 (59·2%)	13 568 (59·2%)
	Spontaneous	8955 (39·2%)	8967 (39·2%)
	No labour	376 (1·7%)	367 (1·6%)
Epidural analgesia‡			
	Yes	2682 (26·0%)	2766 (26·8%)
	No	7628 (74·0%)	7549 (73·2%)
	Unknown	12 677	12 740
Presence of meconium‡			
	Yes	449 (4·5%)	454 (4·5%)
	No	9454 (95·5%)	9535 (95·5)
	Unknown	13 084	13 066

Data are median (IQR), n, or n (%). Missing data are <1%, unless otherwise presented; there were no apparent differences in missing data between trial arms.

* Women with more than one birth episode in the study period are included more than once (n=454).

† As coded by the UK National Health Service.

‡ These data were collected only from 2013 at most centres.

**Table 2:** Primary and secondary outcomes

		**Decision support (n=23 263)**	**No decision support (n=23 351)**	**Adjusted risk ratio (CI)**
**Composite neonatal primary outcome**				
Composite primary outcome*		172 (0·7%)	171 (0·7%)	1·01 (95% CI 0·82–1·25)
	Intrapartum stillbirths†	1 (0)	2 (0)	0·50 (95% CI 0·05–5·53)
	Neonatal deaths up to 28 days after birth‡	6 (0)	4 (0)	1·51 (95% CI 0·42–5·33)
	Moderate or severe neonatal encephalopathy (requiring cooling)	18 (0·1%)	21 (0·1%)	0·86 (95% CI 0·46–1·61)
	Admission to neonatal unit within 48 h of birth for ≥48 h because of feeding difficulties, respiratory illness or symptoms, or encephalopathy and evidence of compromise at birth	147 (0·6%)	144 (0·6%)	1·02 (95% CI 0·81–1·29)
**Other neonatal outcomes**				
Admission to a higher level of care		1389 (6·0%)	1429 (6·1%)	0·98 (99% CI 0·89–1·08)
Apgar score <4 at 5 min		43 (0·2%)	65 (0·3%)	0·67 (99% CI 0·40–1·11)
Cord artery pH				
	<7·15	1625 (11·3%)	1695 (11·8%)	0·96 (99% CI 0·88–1·04)
	<7·05	268 (1·9%)	278 (1·9%)	0·95 (99% CI 0·77–1·19)
	Mean (SD)	7·24 (0·08)	7·24 (0·08)	··
	Unknown	8829	8981	··
Metabolic acidosis§				
	Yes	148 (1·1%)	131 (1·0%)	1·12 (99% CI 0·82–1·52)
	No	13 538 (98·9%)	13 533 (99·0%)	··
	Unknown	9577	9687	··
Resuscitation				
	None	18 457 (87·3%)	18 605 (87·6%)	··
	One intervention	2139 (10·1%)	2116 (10·0%)	1·03¶ (99% CI 0·96–1·09)
	Two or more interventions	554 (2·6%)	524 (2·5%)	··
	Unknown	2113	2106	··
Seizures in hospital		39 (0·2%)	41 (0·2%)	0·95 (99% 0·54–1·70)
Destination of baby immediately after birth				
	Postnatal ward	21 571 (93·6%)	21 664 (93·6%)	··
	Home	467 (2·0%)	485 (2·1%)	1·00|| (99% CI 0·99–1·00)
	Transitional care unit	277 (1·2%)	235 (1·0%)	··
	Neonatal unit	653 (2·8%)	690 (3·0%)	··
	Transferred hospital	4 (0)	7 (0)	··
	Stillbirth	1 (0)	2 (0)	··
	Other	69 (0·3%)	53 (0·2%)	··
Median length of hospital stay, days		2 (1–3)	2 (1–3)	0·99** (99% CI 0·97–1·01)
**Delivery outcomes**				
Mode of delivery				
	Spontaneous cephalic vaginal	11 823 (50·8%)	11 959 (51·2%)	0·99 (99% CI 0·97–1·01)
	Caesarean section	5669 (24·4%)	5555 (23·8%)	··
	Instrumental	5698 (24·5%)	5765 (24·7%)	··
	Vaginal breech	73 (0·3%)	72 (0·3%)	··
Indications for any operative intervention (caesarean section and instrumental delivery)				
	Fetal distress	4278 (18·4%)	4262 (18·3%)	1·04† † (99% CI 1·00–1·08)
	Failure to progress	5059 (21·8%)	5175 (22·2%)	1·01† † (99% CI 0·97–1·05)
	Fetal distress and failure to progress	1774 (7·6%)	1599 (6·9%)	··
	Other reason	229 (1·0%)	247 (1·1%)	··
Indication for instrumental vaginal deliveries				
	Fetal distress	2608 (11·2%)	2559 (11·0%)	1·03† † (99% CI 0·97–1·09)
	Failure to progress	2262 (9·7%)	2396 (10·3%)	0·97† † (99% CI 0·91–1·03)
	Fetal distress and failure to progress	700 (3·0%)	660 (2·8%)	··
	Other reason	117 (0·5%)	134 (0·6%)	··
Caesarean section				
	Grade 1 (immediate threat to life)	1138 (4·9%)	1121 (4·8%)	1·02‡ ‡ (99% CI 0·92–1·13)
	Grade 2 (some threat of compromise)	3754 (16·2%)	3605 (15·5%)	1·04‡ ‡ (99% CI 0·99–1·09)
	Grade 3 (no threat of compromise)	645 (2·8%)	689 (3·0%)	1·02‡ ‡ (99% CI 0·98–1·07)
	Grade 4 (elective)	12 (0·1%)	12 (0·1%)	··
Episiotomy§§				
	Yes	6396 (28·9%)	6498 (29·3%)	0·99 (99% CI 0·95–1·03)
	Unknown	826	840	··
Any episode of fetal blood sampling§§		2366 (10·3%)	2187 (9·5%)	1·08 (99% CI 1·01–1·16)
Destination of mother immediately after birth§§				
	Ward	21 554 (94·6%)	21 614 (94·5%)	··
	Home	429 (1·9%)	462 (2·0%)	1·00¶¶ (99% CI 0·99–1·00)
	Intensive-care unit	15 (0·1%)	19 (0·1%)	··
	High-dependency unit	793 (3·5%)	768 (3·4%)	··
	Theatre	0 (0)	0 (0)	··
	Other hospital	0 (0)	8 (0)	··
Admission to a higher level of care§§		1245 (5·4%)	1193 (5·2%)	1·05 (99% CI 0·95–1·16)
**Duration of labour**				
No labour		378	371	··
Seemingly randomised after delivery		92	120	··
Length of labour from trial entry (min)||||				
	Geometric mean and geometric mean ratio	379	381	0·99 (99% CI 0·98–1·01)
	Median	404 (234–638)	408 (236–640)	··
	Unknown	871	924	··
Length of first stage from trial entry (min)||||				
	Geometric mean and geometric mean ratio	169	168	1·01 (99% CI 0·98–1·04)
	Median	200 (100–351)	201 (96–354)	··
	Unknown	6422	6292	··
Length of second stage from trial entry (min)||||				
	Geometric mean and geometric mean ratio	39	39	0·99 (99% CI 0·96–1·03)
	Median	49 (15–113)	50 (16–114)	··
	Unknown	6036	5934	··

Data are n (%), n, or median (IQR), unless otherwise specified. Missing data are <1% unless otherwise presented. Risk ratios were adjusted for stratification factors used in the randomisation (centre and twin birth) and clustering because of twins and multiple birth episodes. Minimisation factors were not adjusted for in the analysis of intrapartum stillbirths, neonatal deaths, and neonatal encephalopathy because of the small number of events. Crude effect measures were not presented as identical to one decimal place (two decimal places for most outcomes). CI=confidence interval.

* The components of the primary outcome are mutually exclusive and outcomes listed higher take precedence over those listed below them—eg, if a baby with neonatal encephalopathy died within 28 days, the outcome would be recorded as neonatal death.

† Excludes stillbirths due to congenital anomalies.

‡ Excludes deaths due to congenital anomalies; deaths after hospital discharge not reported for Ireland.

§ Cord artery pH <7·05, base deficit ≥12mmol/L.

¶ Risk ratio of one or more interventions *vs* none.

|| Risk ratio of ward or home *vs* all other destinations (if known).

** This figure is a hazard ratio rather than a risk ratio.

†† Risk ratio for fetal distress and risk ratio for failure to progress include (in the numerator) deliveries in the third category for which both fetal distress and failure to progress were recorded.

‡‡ Risk ratios based on cumulative totals for grade—ie, grade 1 *vs* all other deliveries, grade 1–2 *vs* all other deliveries, and grade 1–3 *vs* all other deliveries.

§§ n=22 987 in the decision-support group and 23 055 in the no-decision-support group (ie, the number of women, rather than the number of infants) for all outcomes after this row.

¶¶ Risk ratio of ward or home *vs* all other destinations (if known).

|||| Denominators exclude women with no labour, seemingly randomised after delivery, and unknown length of labour.

**Table 3:** Process outcomes after trial entry

		**Decision support (n=22 517)**	No decision support*(n=22 564)	**Adjusted effect measure (99% CI)**
Epidural analgesia				
	Yes	2770 (27·3%)	2689 (26·5%)	Risk ratio 1·03 (99% CI 0·97–1·09)
	No	7383 (72·7%)	7453 (73·5%)	··
	Unknown†	12 364	12 422	··
Labour augmentation				
	Yes	2705 (30·9%)	2750 (31·3%)	Risk ratio 0·99 (99% CI 0·93–1·04)
	No	6047 (69·1%)	6042 (68·7%)	··
	Unknown†	13 765	13 772	··
Presence of meconium		··	··	
	Yes	440 (4·5%)	469 (4·8%)	Risk ratio 0·94 (99% CI 0·80–1·11)
	No	9316 (95·5%)	9346 (95·2%)	··
	Unknown†	12 761	12 749	··
At least one blue, yellow, or red level of concern		21 950 (97·5%)	22 021 (97·6%)	Risk ratio 1·00 (99% CI 1·00–1·00)
At least one blue level of concern (mild abnormality)		21 863 (97·1%)	21 913 (97·1%)	Risk ratio 1·00 (99% CI 1·00–1·00)
At least one yellow level of concern (moderate abnormality)		16 765 (74·5%)	16 722 (74·1%)	Risk ratio 1·00 (99% CI 0·99–1·02)
At least one red level of concern (severe abnormality)		2335 (10·8%)	2413 (11·1%)	Risk ratio 0·97 (99% CI 0·90–1·04)
	Unknown‡	822	833	··
Blue, yellow, or red levels of concern in women with at least one level of concern				
	Median	9 (5–15)	9 (5–15)	··
	Rate (per h)	1·37	1·40	Rate ratio 0·98 (99% CI 0·96–1·00)
	Unknown§	765	824	
Blue levels of concern in women with a blue level				
	Median	7 (4–11)	7 (4–11)	··
	Rate (per h)	1·06	1·05	Rate ratio 1·01 (99% CI 0·99–1·03)
	Unknown§	740	800	··
Yellow levels of concern in women with a yellow level				
	Median	2 (1–4)	2 (1–5)	··
	Rate (per h)	0·35	0·40	Rate ratio 0·87 (99% CI 0·84–0·89)
	Unknown§	354	421	··
Red levels of concern in women with a red level				
	Median	1 (1–1)	1 (1–1)	··
	Rate (per h)	0·14	0·14	Rate ratio 0·98 (99% CI 0·92–1·04)
	Unknown‡§	41	55	··
Interaction with Guardian system¶				
	Median	5 (0–16)	4 (0–15)	··
	Rate (per h)	4·22	4·21	Rate ratio 0·99 (99% CI 0·95–1·03)
	Unknown	1723	1603	··
Vaginal examinations				
	Median	2 (1–3)	2 (1–3)	··
	Rate (per h)	0·28	0·27	Rate ratio 1·03 (99% CI 1·00–1·05)
	Unknown	877	929	··
Time from last red level of concern to delivery (mins)				
	Median	58 (13–279)	58 (13–264)	HR 0·99 (99% CI 0·92–1·06)
	Unknown‡	822	823	··

Data are n (%), n, or median (IQR), unless otherwise specified. Women with no labour or seemingly randomised after delivery were not included in calculations in this table, which is why the denominators differ from those in the footnotes of table 2. Effect measures were adjusted for stratification factors used in the randomisation (centre and twin birth) and clustering as a result of twins and multiple birth episodes. Crude effect measures are not presented as identical to one decimal place (two decimal places for most outcomes). CI=confidence interval. HR=hazard ratio.

* For the control group with cardiotocographic monitoring only, decision-support software was used retrospectively to determine when an alert would have sounded.

† These data were only recorded and uploaded for analysis from 2013 for most centres.

‡ Data for timing of red level of concerns not available for two centres—Warwick (n=823) and Derby (n=832)·

§ Women with missing length of labour were not included in calculation of rates and rate ratios.

¶ Measured via number of thumbprint entries from time of trial entry to first yellow level of concern, or until cervix fully dilated if no abnormality detected.

**Table 4:** Health and development outcomes at 2 years in a sample of surviving infants without the primary outcome selected for follow-up

		**Decision support (n=3556)**	**No decision support (n=3510)**	**Adjusted effect measure (CI)**
PARCA-R composite score				
	Mean (SD)	98·0 (33·8)	97·2 (33·4)	Mean difference 0·63 (95% CI −0·98 to 2·25)
	Median (IQR)	98 (73–126)	97 (72–125)	··
	Unknown	175	184	··
PARCA-R non-verbal cognition scale				
	Mean (SD)	27·7 (3·7)	28·0 (3·6)	Mean difference −0·22 (99% CI −0·44 to 0·01)
	Median (IQR)	28 (26–30)	28 (26–31)	··
PARCA-R vocabulary sub-scale				
	Mean (SD)	57·4 (27·8)	56·5 (27·7)	Mean difference 0·82 (99% CI −0·91 to 2·54)
	Median (IQR)	58 (36–81)	56 (35–80)	··
PARCA-R sentence complexity sub-scale				
	Mean (SD)	12·4 (5·4)	12·3 (5·3)	Mean difference 0·07 (99% CI −0·26 to 0·41)
	Median (IQR)	12 (9–16)	12 (9–16)	··
Cerebral palsy				
	n (%)	4 (0·1%)	4 (0·1%)	RR 0·99 (99% CI 0·16 to 6·10)
	Unknown (n)	111	114	··
Non-major or major disability*		··	··	RR 1·08 (99% CI 0·98 to 1·18)
	n (%)	942 (40·4%)	840 (37·4%)	··
	Unknown (n)	1225	1266	··
Major disability*				
	n (%)	134 (5·7%)	135 (6·0%)	RR 0·95 (99% CI 0·70 to 1·29)
	Unknown (n)	1225	1266	··

Missing data are <3% unless otherwise presented; there were no apparent differences in missing data between trial arms. Effect measures were adjusted for stratification factors used in the randomisation (centre and twin birth) and clustering as a result of twins and multiple birth episodes. Stratification factors were not adjusted for in the analysis of infant deaths at 2 years and cerebral palsy because of the small number of events. Crude effect measures were not presented as identical to one decimal place (two decimal places for most outcomes). At 2 years, 29 (0·13%) of 21 508 infants had died in the decision-support group and 35 (0·16%) of 21 597 infants had died in the no-decision-support group (adjusted RR 0·83 [99% CI 0·44–1·59])—all deaths were reported to age 2 years excluding stillbirths (one in the decision-support group and two in the no-decision-support group). Data from Ireland were not included in the numerator (n=1 in the decision-support group) or denominator (n=1754 in decision-support group and n=1752 in the no-decision-support group) because data for deaths after discharge were not available. CI=confidence interval. PARCA-R=Parent Report of Children's Abilities—Revised. RR=risk ratio.

* Disability in any of neuromotor function, seizures, auditory function, communication, visual function, cognitive function, or other physical disability.
