# Supplementary material for: Computerised interpretation of fetal heart rate during labour (INFANT): a randomised controlled trial
Source: Lancet. 2017 Apr 29;389(10080):1719–29. doi: 10.1016/S0140-6736(17)30568-8 (PMC5413601; doi:10.1016/S0140-6736(17)30568-8)
Supplement: Supplementary appendix [file mmc1.pdf]

THE LANCET

Supplementary appendix

This appendix formed part of the original submission and has been peer reviewed. We post it as supplied by the authors.

Supplement to: The INFANT Collaborative Group. Computerised interpretation of fetal heart rate during labour (INFANT): a randomised controlled trial. *Lancet* 2017; published online March 21. http://dx.doi.org/10.1016/S0140-6736(17)30568-8.

Appendix

Contents

1 Further acknowledgements 3

2 The data collection system (Guardian) display 5

3 Review process for primary outcome 7

3.1 Process 7

3.2 Independent Review Panel 8

4 Abbreviated statistical analysis plan (finalised and agreed prior to unblinding and analysis) 8

4.1 Data collection schedule 8

4.2 Derivation of Variables 9

4.2.1 Primary Outcomes 9

4.2.2 Neonatal secondary outcomes 10

4.2.3 Maternal secondary outcomes 11

4.2.4 Quality of care outcome 12

4.2.5 Process outcomes 12

4.2.6 Health and development outcomes at 24 months 13

4.3 Protocol violation 16

4.4 Protocol deviation 16

4.4.1 Participants randomised in error 16

4.4.2 Participants who do not receive allocated intervention 16

4.4.3 Follow up completed outside set time window 17

4.5 Primary Analysis Strategy 17

4.5.1 Descriptive analysis population 17

4.5.2 Comparative analysis population 18

4.5.3 Interim analysis population 18

4.6 Representativeness of Trial Population and Participant Throughput 18

4.7 Baseline Comparability of Randomised Groups 18

4.8 Losses to Follow-up 19

4.9 Description of Available data 19

4.10 Description of Compliance with Intervention 19

4.11 Unblinding of Randomised Treatments 19

4.12 Statistical Methods Used for Analysis of Primary Outcomes 20

4.13 Significance Levels 20

4.14 Missing Data 20

4.15 Pre-specified Subgroup Analysis 21

4.16 Pre-specified Sensitivity Analysis 21

4.17 Statistical Software Employed 21

4.18 Statistical Methods Used for Analysis of Secondary Outcomes 22

4.19 Deviation from Analysis Described in Protocol 22

4.20 References 22
Annex 24
Additional tables and figures 26
Table S1: List of participating centres 26
Table S2: Reasons for withdrawal 26
Table S3: Reasons for randomised in error 27
Table S4: Sensitivity analysis of composite primary outcome (panel review score ≥7 versus ≥3) 27
Table S5: Quality of care outcomes 27
Table S6: Quality of care outcomes (all babies in denominator) 28
Table S7: Number of levels of concern after trial entry (all babies in denominator) 28
Table S8: Maternal characteristics at trial entry by follow-up status: responders versus non responders or not followed up at 2 years (mothers of surviving infants without the trial primary outcome only) 29
Table S9: Maternal characteristics at trial entry by follow-up status: responders versus non responders at 2 years (mothers of surviving infants without the trial primary outcome only) 30
Table S10: Components of non-major and major disability at 2 years 31
Subgroup analyses 32
Figure S1: Maternal and neonatal outcomes by twin pregnancy 32
Figure S2: Maternal and neonatal outcomes by suspected fetal growth restriction 33
Figure S3: Maternal and neonatal outcomes by body mass index at booking visit 34
Figure S4: Composite primary outcome by centre 35
Figure S5: Instrumental delivery or caesarean section by centre 36
Figure S6: Caesarean section by centre 37
Figure S7: Admission of infant to a higher level of care by centre 38
Figure S8: Apgar score by centre 39
Figure S9: Cord artery pH by centre 40
Figure S10: Metabolic acidosis by centre 41
Figure S11: Resuscitation by centre 42
Figure S12: Seizures by centre 43
Figure S13: Baby destination immediately after birth by centre 44
Figure S14: Length of hospital stay to discharge by centre 45
Figure S15: Epidural analgesia after trial entry by centre 46
Figure S16: Labour augmentation after trial entry by centre 47
Figure S17: Presence of meconium after trial by centre 48
Figure S18: Number of women with at least one blue, yellow or red level of concern by centre 49
Figure S19: Number of women with at least one blue level of concern by centre 50
Figure S20: Number of women with at least one yellow level of concern by centre 51
Figure S21: Number of women with at least one red level of concern by centre 52
Figure S22: Number of blue, yellow and red levels of concern by centre 53
Figure S23: Number of blue levels of concern by centre 54
Figure S24: Number of yellow levels of concern by centre 55
Figure S25: Number of red levels of concern by centre 56
Figure S26: Time from last red level of concern to delivery 57
Figure S27: Histograms of cord artery pH by trial allocation 58

Subgroup analyses 58
Table S5: PARCA-R Composite score by twin pregnancy 59
Table S6: PARCA-R Composite score by suspected fetal growth restriction (FGR) 59
Table S7: PARCA-R Composite score by body mass index 59
Table S8: PARCA-R Composite score by centre 60

1 Further acknowledgements

Writing committee

- Peter Brocklehurst, Professor of Women's Health, Director of Birmingham Clinical Trials Unit, University of Birmingham
- David John Field, Professor, Department of Health Sciences, University of Leicester
- Keith Richard Greene, Retired from 2011 from Plymouth Hospitals NHS Trust and University of Plymouth now research contract with University College London
- Ed Juszczak, Associate Professor, Director, National Perinatal Epidemiology Unit (NPEU) Clinical Trials Unit, Nuffield Department of Population Health, University of Oxford
- Sara Kenyon, Reader in Evidence Based Maternity Care, Institute of Applied Health Research, University of Birmingham
- Louise Linsell, Senior Medical Statistician, National Perinatal Epidemiology Unit (NPEU) Clinical Trials Unit, Nuffield Department of Population Health, University of Oxford
- Christopher Mabey, Engineer, K2 Medical Systems, Plymouth.
- Mary Newburn, Consultant, Health Researcher/Public and Parent Involvement Lead (Maternity theme), CLAHRC South London, Kings College London
- Rachel Plachcinski, Research Engagement Officer, National Childbirth Trust
- Maria Quigley, Professor of Statistical Epidemiology, National Perinatal Epidemiology Unit (NPEU), Nuffield Department of Population Health, University of Oxford
- Elizabeth Schroeder, Senior Research Fellow, Centre for Health Economy, Australian Hearing Hub, Macquire University, New South Wales, Sydney
- Philip Steer, Emeritus Professor, Imperial College London

Co-investigator Group

- Peter Brocklehurst, Professor of Women's Health, Director of Birmingham Clinical Trials Unit, University of Birmingham
- David John Field, Professor, Department of Health Sciences, University of Leicester
- Keith Richard Greene, Retired from 2011 from Plymouth Hospitals NHS Trust and University of Plymouth now research contract with University College London

- Ed Juszczak, Associate Professor, Director, National Perinatal Epidemiology Unit (NPEU) Clinical Trials Unit, Nuffield Department of Population Health, University of Oxford
- Robert Keith, Director, K2 Medical Systems, Plymouth
- Sara Kenyon, Reader in Evidence Based Maternity Care, Institute of Applied Health Research, University of Birmingham
- Louise Linsell, Senior Medical Statistician, National Perinatal Epidemiology Unit (NPEU) Clinical Trials Unit, Nuffield Department of Population Health, University of Oxford
- Christopher Mabey, Engineer, K2 Medical Systems, Plymouth.
- Mary Newburn, Consultant, Health Researcher/Public and Parent Involvement Lead (Maternity theme), CLAHRC South London, Kings College London
- Rachel Plachcinski, Research Engagement Officer, National Childbirth Trust
- Maria Quigley, Professor of Statistical Epidemiology, National Perinatal Epidemiology Unit (NPEU), Nuffield Department of Population Health, University of Oxford
- Elizabeth Schroeder, Senior Research Fellow, Centre for Health Economy, Australian Hearing Hub, Macquire University, New South Wales, Sydney
- Philip Steer, Emeritus Professor, Imperial College London

Clinical trials unit staff:

National Perinatal Epidemiology Unit, University of Oxford (from January 2009 to May 2012):

- Vicki Barber, Trial Director
- Emma Haines, Data and Administrative Coordinator
- Andy Kirk, Webmaster and Design Coordinator
- Louise Linsell, Senior Medical Statistician
- Katie Lean, Recruiting Midwife Coordinator
- Linda Mottram, Trial Coordinator
- Liz Schroeder, Perinatal Economist
- Clare Shakeshaft, Study Co-ordinator

Comprehensive Clinical Trials Unit, UCL (from 1 May 2012 to 1 June 2016):

- Julie Bakobaki, Clinical Project Manager
- Philip Bakobaki, IT Developer
- James Blackstone, Data Manager
- Mary Yip Braidley, Trial Manager
- Jackie Coleman, Trial Coordinator
- Jade Dyer, Data Manager
- Abigail Howarth, Data Coordinator
- Dawn Letchford, Data Entry Assistant
- Victoria McCudden, Clinical Project Manager
- Guy Schroeter, Clinical Project Manager
- Heather Short, Trial Manager
- Irene Simmonds, Trial Manager

2 The data collection system (Guardian) display

Example of a 'blue level of concern' screenshot:

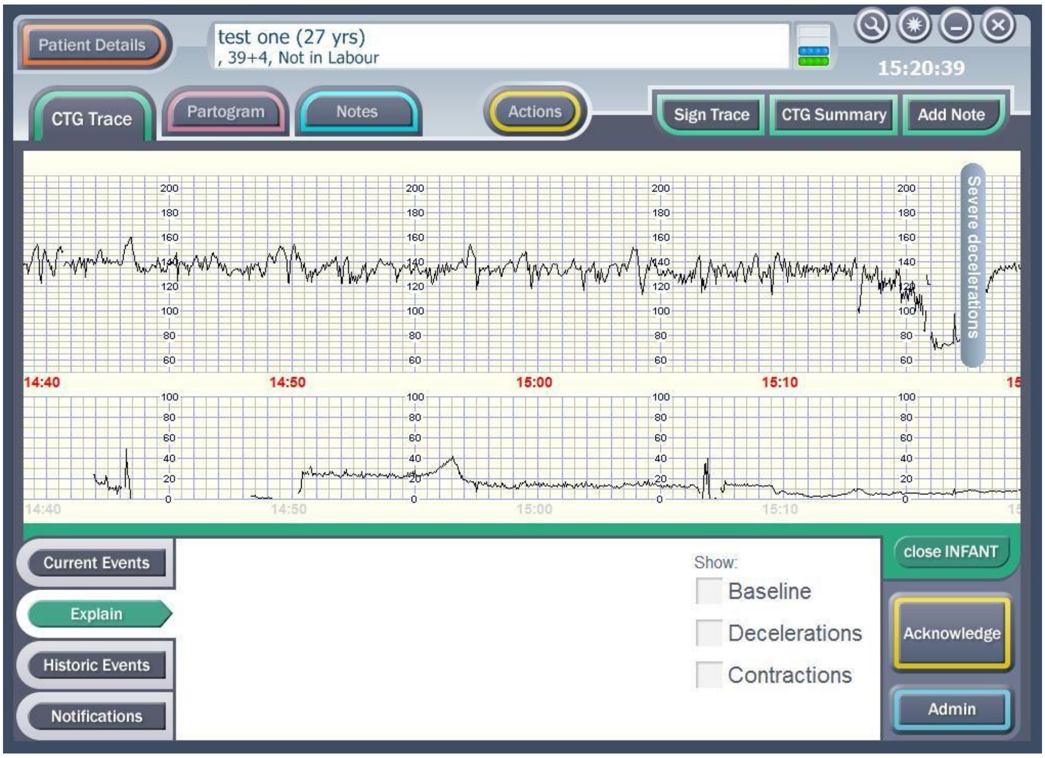

Example of a 'yellow level of concern' screenshot:

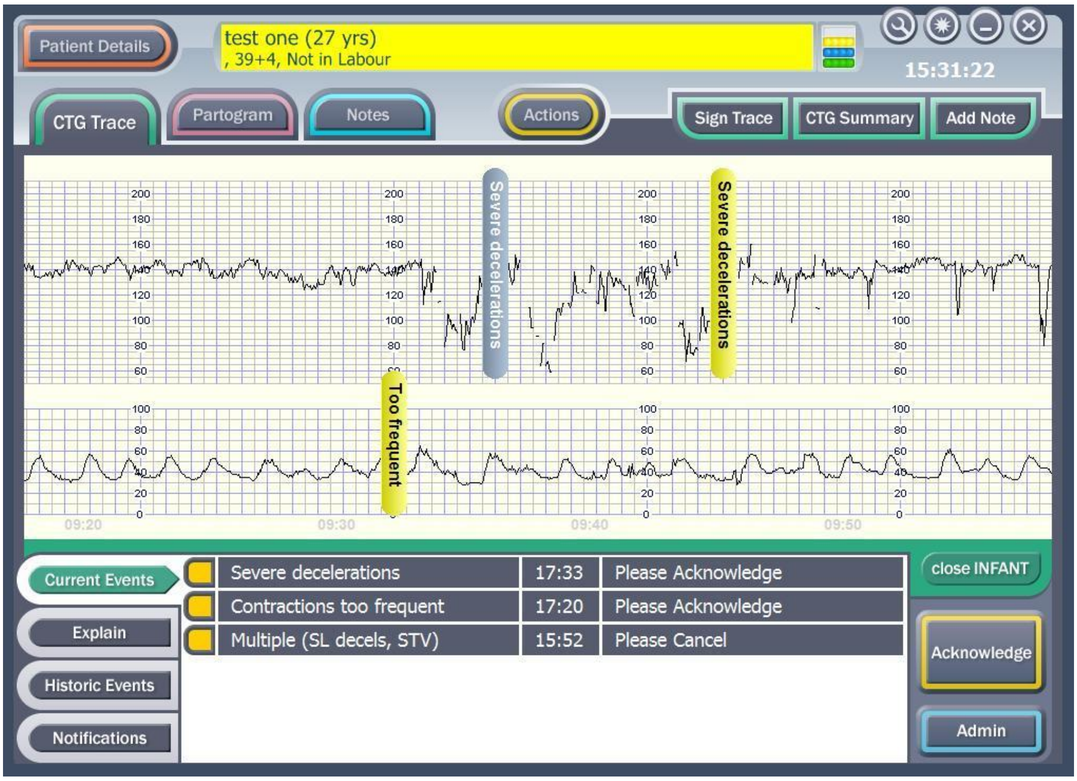

Example of a 'red level of concern' screenshot:

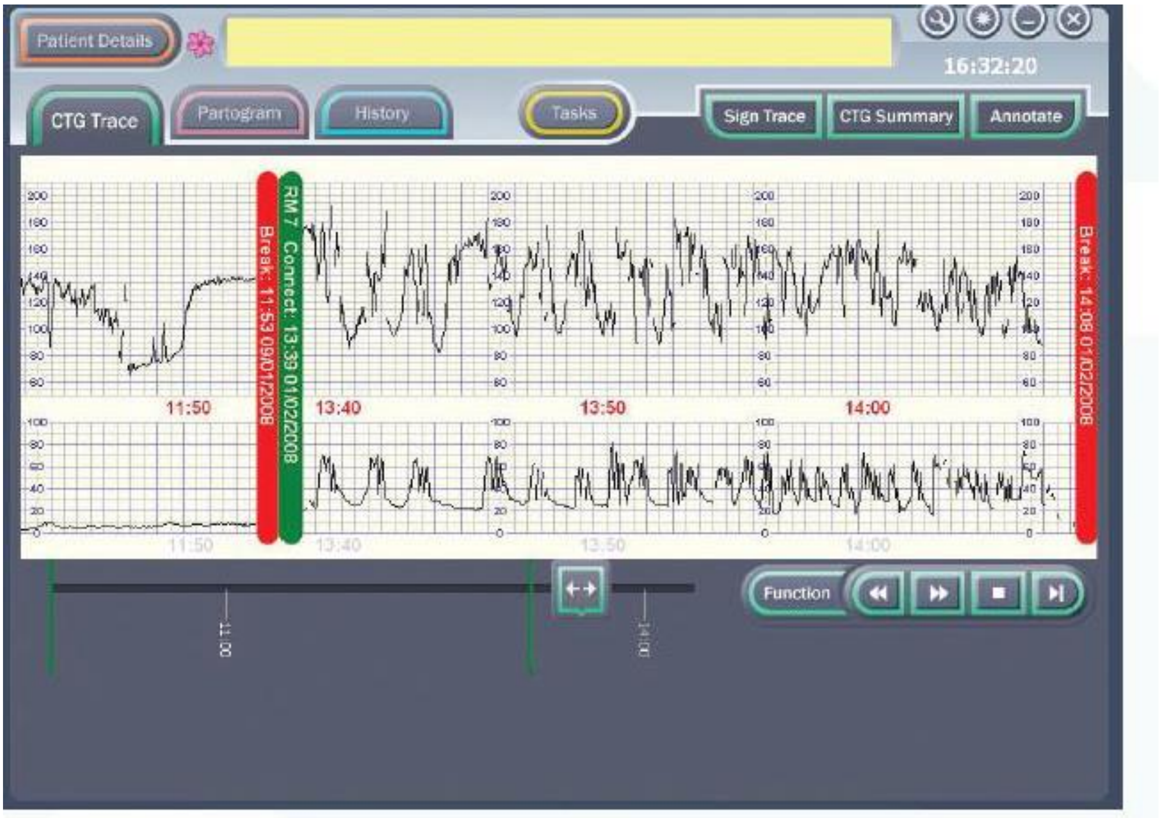

3 Review process for primary outcome

Following the Trial Steering Committee meeting on the 2nd October 2013, the INFANT Project Management Group agreed to undertake further work to clarify the classification of the primary outcome for the trial. This issue had arisen because the incidence of one of the components of the composite primary outcome (Admission to the neonatal unit within 48 hours of birth for at least 48 hours with evidence of respiratory or feeding problems or NNE), was substantially higher than had been anticipated, and an initial review of these cases (by Peter Brocklehurst and David Field, blinded to allocation), suggested a number of cases were included in this outcome which were unrelated to perinatal hypoxia. It was also recognised that for many of these uncertain cases, the detail provided by the INFANT data collection forms was insufficient (Annex).

It was agreed that neonatal unit discharge summaries would be collected for all babies who were admitted to the neonatal unit within 48 hours of birth for more than 48 hours. A more formal process of review of a proportion of these cases would then be undertaken by Peter Brocklehurst, David Field, Keith Greene and Nikki Robertson (a neonatologist unconnected with the trial who is an expert on perinatal hypoxia in term babies) to devise a more structured way of assessing the classification of these babies. This system would then be implemented by David Field and Keith Greene for the purposes of data monitoring. There was also agreement that this process would be repeated by an independent panel of neonatologists at the end of the trial.

Peter Brocklehurst, David Field, Keith Greene and Nikki Robertson met on the 16th January 2013 and reviewed approximately 40 sets of discharge summaries (blinded to trial allocation) and devised a data extraction form which included the key elements of the neonatal course which are most likely to be related to intrapartum hypoxia. These are included in the annex. This form is not an established or validated list of criteria and the numeric scoring was devised to give some quantification of the severity of elements of the clinical course. A score of 3 or greater was agreed to be evidence that the condition of the baby was likely to be associated with intrapartum hypoxia, acknowledging that there remained uncertainty about this, as there is no absolute measure of intrapartum hypoxia.

However, having reviewed the discharge summaries with the data extraction form, the criteria were felt to be a good reflection of the baby's clinical condition.

3.1 Process

David Field and Keith Greene then reviewed all the discharge forms independently to score the cases. A teleconference on the 22nd February 2012 resolved, by consensus with Peter Brocklehurst, the uncertain cases so that a decision was made to include each baby as having evidence of the primary outcome or not.

This produced a slightly revised definition of this component of the composite primary outcome:

Admission to the neonatal unit within 48 hours of birth for at least 48 hours with respiratory or feeding problems or NNE (where there is evidence of compromise at birth).

This process and revised definition were reviewed and accepted by the TSC on 18th March 2013 and the DMC on 21st March 2013.

Cases continued to be reviewed by David Field and Keith Greene on a monthly basis for the purposes of data monitoring. These data were entered on to the INFANT database accordingly.

3.2 Independent Review Panel

An independent panel of neonatologists, to review all cases of the primary outcome (all stillbirths, all early neonatal deaths, all cases of NNE and all babies admitted to the neonatal unit within 48 hours but for more than 48 hours with evidence of respiratory or feeding difficulties), was assembled. An advert was placed in the British Association of Perinatal Medicine newsletter for prospective panel members. In order to ensure independence of the panel, members could not be practising at any of the INFANT trial recruiting centres. Brief information of what will be expected as part of the review, how often they will be required to meet, and details of payments to be made were included. The role will be remunerated at £500 per day. Applicants were selected by the INFANT co-investigators group.

After an initial face-to-face meeting to establish methods of working, panel members were sent a proportion of the cases once trial recruitment had finished. There was overlap of cases so that every case was reviewed by at least two reviewers. It was estimated that there would be approximately 1000 cases to review. Where there was lack of agreement by two members of the panel, consensus was reached by discussion with the whole panel, at either a face-to-face meeting or teleconference.

4 Abbreviated statistical analysis plan (finalised and agreed prior to unblinding and analysis)

4.1 Data collection schedule

Information will be collected at the following times:

- Electronically during labour via the Guardian® system
  All women
- Post Birth Data Collection Form M (Mother)
  All women receiving a higher level of care, surgery or a procedure in theatre following delivery
- Post Birth Data Collection Form B (Baby)
  All babies receiving a higher level of care or surgery following birth
- Post Birth Data Collection Chart B (Baby) for Neonatal Encephalopathy

All babies receiving a higher level of care and classified as having Neonatal Encephalopathy

- Death of a Baby in the INFANT Study
All intrapartum stillbirths and neonatal deaths up to 28 days after birth
- Parent Questionnaire at 24 months (health and development outcomes)
All infants with the trial primary outcome and a further subset of 7000 participants consenting to follow up
- Parent Questionnaire at 12 and 24 months (health economic items)
All infants with the trial primary outcome and a subset of 700 participants within the follow up sample of 7000

4.2 Derivation of Variables

4.2.1 Primary Outcomes

- Composite of 'poor neonatal outcome', defined as:
  - Intrapartum stillbirths except deaths due to congenital anomalies

	Baby Outcome *= "Stillbirth" on Guardian®*
**MINUS**	*Any deaths due to congenital anomalies recorded on the Death of a Baby in the INFANT Study Form*

OR

  - Neonatal deaths up to 28 days after birth except deaths due to congenital anomalies

	Baby Outcome *= "Early neonatal death" on Guardian® and died within 28 days after birth*
**OR**	*A "Yes" response to* ***Q14*** *on the PBDC B (Baby) form and died within 28 days after birth*
**OR**	*A completed Death of a Baby in the INFANT Study Form and died within 28 days after birth*
**OR**	*Deaths notified by the NHS Information Centre (England) and NHS Greater Glasgow & Clyde Safe Haven (Scotland) up to 28 days after birth*
**MINUS**	*Any deaths due to congenital anomalies recorded on the Death of a Baby in the INFANT Study Form*

OR

  - Moderate or severe encephalopathy

	A "Yes" response to therapeutic hypothermia on the *PBDC B (Baby) Chart for NNE*
**AND**	*Confirmed by a blinded review committee*

OR

- Admission to neonatal unit within 48 hours of birth for ≥ 48 hours with evidence of feeding difficulties, respiratory illness or encephalopathy

	*A "Yes" response to **Q2** on the PBDC B (Baby) Form*
**AND**	*A "Yes" response to **Q5 OR Q6** on the PBDC B (Baby) Form*
**AND**	*A "Yes" response to **Q8** on the PBDC B (Baby) Form*
**AND**	*A "Yes" response to **Q9** on the PBDC B (Baby) Form*
**AND**	*Confirmed as compromised at birth by a blinded review committee (score≥3 on Primary Outcome Review Scoring Sheet).*

- Parent Report Composite Score at 24 months:
  Total sum of scores on the Parent Report of Children's Abilities (PARCA-R) in section A, B and C of the Parent Questionnaire at 24 months.

4.2.2 Neonatal secondary outcomes

- Intrapartum stillbirth except deaths due to congenital anomalies

- Neonatal deaths up to 28 days after birth except deaths due to congenital anomalies

- Moderate or severe encephalopathy

- Admission to neonatal unit within 48 hours of birth for ≥ 48 hours with evidence of feeding difficulties, respiratory illness or encephalopathy

  (For the above, see definition in 3.2.1)

- Admission to higher level of care within 48 hours of birth for ≥ 48 hours
  *A "Yes" response to **Q2** on the PBDC B (Baby) Form*

- Apgar score <4
  *From the variable **Apgars 5 mins** on Guardian®*

- Distribution of cord blood gas data for cord artery pH
  *Summarise the variable **Cord artery results** on Guardian®*

- Metabolic acidosis
  *Cord artery pH <7.05 and base excess/deficit ≤|12|mmol/l for the variable **Cord artery results** on Guardian®*

- Resuscitation interventions
  *Count of the number of interventions listed under **Resuscitation Type** on Guardian®*

- Seizures
  A "Yes" response to Q7 on the PBDC (Baby) Form

- Destination immediately after birth
  *Summarise the variable **infant transfer dest** on Guardian®*

- Length of hospital stay (baby)

	***Infant Discharge Date** on Guardian®*
**OR**	*Date of discharge recorded in **Q12** of the PBDC B (Baby) Form*
**MINUS**	***Delivery Date Time** on Guardian®*

4.2.3 Maternal secondary outcomes

- Mode of delivery
  *Recorded in Guardian® in field **Mode of Delivery***

- Operative intervention (caesarean section and instrumental delivery) for:
  i. fetal distress, or
  ii. failure to progress, or
  iii. combination of fetal distress and failure to progress, or
  iv. other reason
  *Indications on Guardian® recorded in the fields: **Forceps Indicators**, **Ventouse Indicators** and **CS Indicators** will be coded into these four categories according the rules listed in Annex. Where a woman has more than one indication recorded and at least one is fetal distress and at least one is failure to progress, she will be classified as category iii.*

- Grade of Caesarean section
  *Recorded in Guardian® in field **CS Priority***

- Episiotomy
  *Recorded in Guardian® in field **Episiotomy***

- Any episode of fetal blood sampling
  *Recorded in Guardian® in field **No of FBS***

- Length of first stage, second stage and total length of labour from trial entry
  *Recorded in Guardian®, will be calculated from fields **Labour start time**, **Second stage / fully dilated** and **Delivery Date Time***

- Destination immediately after birth
  *Recorded in Guardian® in field **Mother transfer dest***

4.2.4 Quality of care outcome

- Adverse outcome and suboptimal care

	Composite primary outcome (see definition in 3.2.1)
**AND**	*Metabolic acidosis* (see definition in 3.2.2)
**AND**	*Judged to have experienced suboptimal care in labour by a panel of experienced clinicians blinded to allocation*
**OR**	*Stillbirth or neonatal death not due to congenital anomaly*

4.2.5 Process outcomes

Process outcomes will be derived for the control group in the same way as the intervention group; the decision support software will be running in the background even though it is not displayed, so it is possible to review the pattern of alerts retrospectively across the trace.

- Number of CTG abnormalities (blue, yellow and red levels of concern) detected by the decision support software, after trial entry
  Recorded in Guardian® in fields ***Number of Blue after randomisation, Number of Yellow after randomisation*** *and* ***Number of Red after randomisation***

- Number of blue levels of concern on the decision support software, indicating a mild abnormality on the CTG, after trial entry
  Recorded in Guardian® in field ***Number of Blue after randomisation***

- Number of yellow levels of concern on the decision support software, indicating a moderate abnormality on the CTG, after trial entry
  Recorded in Guardian® in field ***Number of Yellow after randomisation***

- Number of red levels of concern on the decision support software, indicating a severe abnormality on the CTG, after trial entry
  Recorded in Guardian® in field ***Number of Red after randomisation***

- Number of women with at least one yellow level of concern on the decision support software, indicating an abnormality on the CTG, after trial entry
  Recorded in Guardian® in field ***Number of Yellow after randomisation***

- Number of women with at least one red level of concern on the decision support software, indicating a severe abnormality on the CTG, after trial entry
  Recorded in Guardian® in field ***Number of Red after randomisation***

- Time from first red level of concern after trial entry to birth
  Recorded in Guardian®, will be calculated from fields ***Date Time 1st Red after randomisation*** *and* ***Delivery Date Time***

- Number of thumb entries per hour from time of trial entry to first yellow level of concern or until fully dilated (10cm) if no abnormality detected or first yellow level of concern occurred prior to trial entry
  *Recorded in Guardian® in fields **Count of thumbprints between randomisation and first yellow** and **Count of thumbprints between randomisation and fully dilated**. The rate will be calculated using the fields **Date time 1st yellow after randomisation, Second stage / fully dilated** and **Delivery Date Time**.*

- Number of vaginal examinations after trial entry
  *Recorded in Guardian® in field **No of VEs after randomisation***

- Epidural analgesia after trial entry
  *Recorded in Guardian® in fields **PD did woman have an epidural** and **PD did woman have an epidural time.** Only count if first recorded after the time of trial entry.*

- Labour augmentation after trial entry
  *Recorded in Guardian® in fields, **Syntocinon in 1st or 2nd stage** and **Syntocinon in 1st or 2nd stage time.** Only count if first recorded after the time of trial entry.*

- Presence of meconium after trial entry
  *Recorded in Guardian® in field **PD any meconium recorded during labour** and **PD any meconium recorded during labour time.** Only count if first recorded after the time of trial entry.*

4.2.6 Health and development outcomes at 24 months

- Components of the PARCA-R
  - Non-Verbal Cognition Scale
    *Sum of scores in **Section A** of the Parent Questionnaire at 24 months: "Your child at play" **Q1-Q33***
  - Vocabulary Sub-scale
    *Sum of scores of the 100 items in **Section B** of the Parent Questionnaire at 24 months: "What your child can say"*
  - Sentence Complexity Sub-scale
    *Sum of scores in **Section C** of the Parent Questionnaire at 24 months: "Your child's understanding" **Q1-Q18***

- Infant deaths at 24 months

	***Baby Outcome** = "Early neonatal death" on Guardian®*
**OR**	*A "Yes" response to **Q14** on the PBDC B (Baby) form*
**OR**	*A completed Death of a Baby in the INFANT Study Form*

**MINUS**	*Any deaths due to congenital anomalies recorded on the Death of a Baby in the INFANT Study Form*
**AND**	*Deaths notified by the NHS Information Centre (England) and NHS Greater Glasgow & Clyde Safe Haven (Scotland) up to 2 years after birth*

- Disability status at 2 years. Classify as non-major disability or major disability at 2 years if the infant meets any one of the following criteria from the *Parent Questionnaire at 24 months* (criteria for major disability in bold italics):[1,2]

**Sections A-C: Cognitive function**	*PARCA-R composite score 2 to 3 standard deviations below mean for age.*<br>***PARCA-R composite score more than 3 standard deviations below mean for age.***
**Sections B-C: Communication**	*Less than 10/100 words from word list*<br>*At least 10/100 words from word list and Qu C6="Not yet" to the question, "Has your child begun to put words together yet".*<br>***Does not use any recognisable words (including signed words)***<br>***Does not use any sounds that can be understood***<br>***Does not show understanding of any words or signs***
**Section D: Physical ability**	**Walking:**<br>*Has an unsteady walk but doesn't need help*<br>***Unable to walk without help***<br>***Unable to walk even with help***<br>**Sitting:**<br>*Sits unsupported but unstable*<br>*Sits only with support*<br>***Unable to sit***<br>**Hand use:**<br>*Picks up by other means (right or left)*<br>***Unable to pick up object (right or left)***<br>*Has difficulty using one hand*<br>***Unable to use both hands***<br>**Head control:**<br>*Poor control but does not need support*<br>***Can control head only with support***
**Section E: Vision**	*Some difficulty but sees well enough for everyday activities*<br>*Has considerable difficulty but can see objects if near*<br>***Is able to see light only or has no vision***

**Section F: Hearing**	*Has some hearing problems but does not need a hearing aid*<br>*Hears well or with only a little difficulty with a hearing aid*<br>*(Do not count if due to recurrent ear infections or glue ear and no aid)*<br>***Has severe hearing difficulty even with a hearing aid or hearing is not helped with an aid***
**Section G: Growth**	*Height or weight 2 to 3 standard deviations below mean for age*<br>***Height or weight more than 3 standard deviations below mean for age***
**Section G: Seizures**	*Fits, seizures or convulsions (not due to fever) but no treatment required*<br>*On treatment now and has no seizures*<br>*Has up to 1 seizure every month on treatment*<br>***Has more than 1 seizure every month on treatment***
**Section G: Feeding**	***Is fed with tube passed directly into the stomach (gastrostomy)***<br>***Is fed with tube passed from nose to stomach***
**Section G: Respiratory**	*Wheezing more than once a week and taking any medication for chest symptoms when needed*<br>*Currently on any medications for chest symptoms and taking relievers, preventers or steroids every day*<br>***Requires continuous oxygen therapy or mechanical ventilation***
**Section G: Other disability**	*Other long term problem that has some limitation on everyday activities but able to function independently (review case-by-case)*<br>*Needs assistance or aids for some activities*<br>***Is completely dependent on carer***

Missing data mean that the overall classification may result in several categories:

- Major
  At least one component in ***Sections A-G*** *is classified as major*

- At least non-major
  One component in ***Sections A-G*** *is classified as non-major, but where one item is missing and therefore it is not possible to conclude that the overall classification is not major*

- Non-major
  The most severe item in ***Sections A-G*** *is classified as non-major and no other missing data*

- At most non-major
  Where any missing items in ***Sections A-G*** *could only be classified as non-major or no disability and all other items are classified as no disability*

- None
  No data missing and all items in ***Sections A-G*** *is classified as no disability*

- Not known
  *No disability reported but where some data items in **Sections A-G** are missing*

In order to derive the classification **Major** or **Non-major** in the final report, all infants with "At least non-major" disability will be classified as **Non-major**, all infants with "At most non-major" disability will be classified as **None**.

- Diagnosed with cerebral palsy
  *A "Yes'' response to **Q9** in **Section G** of the Parent Questionnaire at 24 months*

- Ever been given breast milk or put to the breast
  *A "Yes'' response in **Section 1** of the Health Economic Parent Questionnaire at 24 months*

- Age when last breast fed or put to the breast (days)
  *Convert response categories to days in **Section 1** of the Health Economic Parent Questionnaire at 24 months*

4.3 Protocol violation

A protocol violation is the intended failure to comply with the final study protocol as approved by the Ethics Committee and Research Department, for example, a serious non-compliance with the protocol resulting from fraud or misconduct that affects participant rights, safety and/or the integrity of the resultant data. Any violations will be reported to the Sponsor and Ethics Committee as soon as possible.

4.4 Protocol deviation

A protocol deviation is an unintended failure to adhere to the final study protocol.
In this trial, the following will be defined as protocol deviations:

4.4.1 Participants randomised in error

These include women:

- who do not receive continuous EFM
- have triplets or a higher order pregnancy
- who are < 35 weeks gestation
- whose infant has a known gross fetal abnormality
- are less than 16 years of age
- are not able to give consent to participate as judged by the attending clinicians
- who received an elective caesarean prior to the onset of labour

4.4.2 Participants who do not receive allocated intervention

These include women:

- in the “CTG with no decision support” arm who receive partial or full decision support
- in the “CTG with decision support” arm who do not receive decision support

4.4.3 Follow up completed outside set time window

These include infants who are aged 2 years ± 3 months when the 2 year follow up questionnaire are completed.

4.5 Primary Analysis Strategy

For the primary analysis, participants will be analysed in the groups into which they were randomly allocated, i.e. comparing the outcomes of all women and babies allocated “CTG with no decision support” with “CTG with decision support”, regardless of allocation received.

The two groups will be compared by calculating the treatment difference adjusted for the stratification factors used in the randomisation (centre and singleton/twin pregnancy). The adjusted analysis will take account of the correlation between treatment groups introduced by stratifying the randomisation (which forces outcomes between treatment arms to be similar apart from any treatment effect)[3]. Both adjusted and unadjusted estimates will be presented for all outcomes, but the primary inference will be based on the adjusted analysis.

The unit of randomisation is birth episode, which raises the issue of non-independence of observations. Some women may have more than one delivery over the study period and may be randomised into the trial more than once if they are eligible, but this is likely to be minimal. Based on national statistics and average interpregnancy intervals in the UK, we anticipate that around 10% of women in this cohort will have a subsequent delivery within the study period, but a smaller proportion will have 2 consecutive births monitored by CTG[4, 5]. Also, around 1.5% of women have twin deliveries[6], but this proportion is likely to be lower in this cohort as some twin births will occur before 35 weeks gestation and a large proportion of twin term births will be by elective caesarean.

We anticipate the proportion of non-independent observations within and between pregnancies to be much less than 10%, however some outcomes may have a large intracluster correlation coefficient (ICC) - in particular the 2 year outcomes collected via parent questionnaire – so clustering will be taken into account in the analysis[7, 8].

4.5.1 Descriptive analysis population

Baseline demographic and clinical characteristics will be reported for each delivery for all women randomised for whom we have data available, excluding protocol violations and women randomised in error who did not give consent or who are under 16 years of age.

4.5.2 Comparative analysis population

- Maternal outcomes
  All women randomised for whom we have data available, excluding protocol violations and women randomised in error who did not give consent or who were under 16 years of age. For women with more than one birth episode during the study period, baseline characteristics and maternal outcomes will be reported on each occasion. For twin births the mode of delivery of the first twin delivered will be reported.

- Short term neonatal outcomes
  All babies including both twins, excluding protocol violations and women randomised in error who did not give consent or who were under 16 years of age.

- 24 month health and development outcomes
  All infants with the trial primary outcome and a sample of 7000 infants recruited during the first two years of the trial, excluding protocol violations, women randomised in error who did not give consent or who were under 16 years of age, and adopted children.

4.5.3 Interim analysis population

Different denominators will be used in the annual interim analysis:

- the total number of trial participants, excluding protocol violations and women randomised in error who did not give consent or who were under 16 years of age
- the number of women with post birth data
- the number of babies with post birth and/or 24 month follow up data.

4.6 Representativeness of Trial Population and Participant Throughput

The flow of participants through each stage of the trial will be summarised using a CONSORT diagram[9]. We will report the numbers of participants:

- randomly assigned
- received intended intervention
- withdrew before or during CTG monitoring
- included in the primary analysis at discharge
- lost to follow up
- included in the analysis at 24 months

4.7 Baseline Comparability of Randomised Groups

Participants in the original two randomised groups will be described separately with respect to:

- maternal age

- ethnic group
- singleton or twin pregnancy
- gestational age at trial entry
- BMI at booking visit (if recorded)
- smoking history at booking visit (if recorded)
- parity
- obstetric history
- cervical dilation at trial entry (if recorded)
- intrauterine growth restriction (IUGR) suspected at labour onset
- labour induction

Numbers (with percentages) for binary and categorical variables and means (and standard deviations), or medians (with lower and upper quartiles) for continuous variables will be presented; there will be no tests of statistical significance performed nor confidence intervals calculated for differences between randomised groups on any baseline variable.

4.8 Losses to Follow-up

The number (with percentages) of losses to follow up among women selected for the 24 month assessment will be reported and compared between the 2 trial arms, and the reasons will be recorded. Any deaths (and their causes) will be reported separately.

4.9 Description of Available data

The pattern of missing data for primary and secondary outcomes, from baseline to the end of follow-up, will be summarised for the two treatment groups with differentiation between fully and partially completed forms/Guardian® fields and those that are missing completely.

Not all data may be routinely collected by all hospitals, e.g. BMI, smoking history, cervical dilation, cord artery pH and base deficit. These data will be reported separately for the subset of hospitals that do collect it routinely.

4.10 Description of Compliance with Intervention

A summary of the intervention received will be provided; this will include intermittent use of the decision support software and/or withdrawal of consent during labour.

4.11 Unblinding of Randomised Treatments

In order to accurately reflect any potential impact of the decision-support software in contemporary NHS practice, such as changes in midwifery presence during labour consequent upon knowledge of the allocation, the clinicians will not be masked to allocation. The local community midwives and participants will also be aware of the allocation. All other persons involved in the trial (except for the Trial Statistician and Trial Programmer), including the UCL trial co-ordinating centre, will not have access to the aggregate list of randomisation codes. K2 Medical Systems will remain blinded until the data are frozen at the end of the trial.

4.12 Statistical Methods Used for Analysis of Primary Outcomes

The number (percentage) of babies with poor neonatal outcome will be presented for each group, and the risk ratio plus 95% confidence interval (CI) will be calculated. Risk ratios will be estimated using generalised estimating equations (GEE), or a similar method, adjusting for the stratification factors used in the randomisation (centre and singleton/twin pregnancy). This method of analysis will account for the correlation in outcomes between twins and siblings delivered in a subsequent pregnancy during the trial period. A log binomial model will be used in the first instance, but if convergence is not achieved then a log Poisson model will be used with a robust variance estimator[10]. The mean (SD) PARCA-R Composite score will be presented for each group, and the mean difference between groups plus 95% CI will be calculated and compared using GEE (Gaussian model with identity link), adjusting for stratification factors.

4.13 Significance Levels

For the analysis of the primary outcomes, a p value of 0.05 (5% significance level) will be used to indicate statistical significance. Comparisons of all other outcomes including the components of the primary outcome will be reported in full for completeness and transparency. For all other analyses, a p value of 0.01 (1% significance level) will be used to indicate statistical significance, to take into account the number of comparisons. Two-sided statistical tests and corresponding p values will be presented throughout.

4.14 Missing Data

Missing data for the short term primary outcome are likely to be negligible, as most of the data are collected electronically on the Guardian® system before the woman leaves the delivery room. If any data items are missing on the PBDC forms, completed for babies and women admitted to a higher level of care, every effort will be made to extract these data from the hospital involved.

For any partially completed PARCA-R scales in the 24 month parent questionnaires, the following strategies for estimation of total and subscale scores will be employed where items are missing:

- Non-Verbal Cognition Scale (PARCA-R: "Your child at play" Q1-Q33)
  Pro rata estimation if less than 10% (up to 3 items) are missing.
  *If **Q6** is "No", and **Q6a** is missing, code "No" for **Q6a**.*
  *If **Q6** is "Don't know", and **Q6a** is missing, code "Don't know" for **Q6a**.*

- Vocabulary Sub-scale (PARCA-R: "What your child can say")
  Non-ticked items will be coded to zero

- Sentence Complexity Sub-Scale (PARCA-R: "Your child's understanding Q1-Q18)
  *If **Q1-Q6** are missing, code to zero and analyse as "Not Yet".*
  *If **Q6** is "Often" or "Sometimes" and any of **Q7-Q18** are not completed, code to zero.*

4.15 Pre-specified Subgroup Analysis

To examine whether the effect of decision-support is consistent across specific subgroups of women and babies, the following subgroup analyses will be undertaken:

- Singletons versus twins
- Suspected IUGR at labour onset versus no growth restriction
- BMI group: underweight (<18.5); normal (18.5-24.9); overweight (25-29.9); obese (>30); unrecorded
- Centre

For the trial composite primary outcome, results will be presented on forest plots showing the risk ratio plus 95% CI for each subgroup, by treatment group, with the p value for the statistical test of interaction[11]. For the PARCA-R score, the difference between the mean treatment effects will be reported, within each subgroup, with a 95% confidence interval and the corresponding p value[12].

Using these statistical methods, we will perform subgroup analyses for all pre-specified neonatal outcomes and instrumental vaginal deliveries. In addition, we will analyse process outcomes by centre.

4.16 Pre-specified Sensitivity Analysis

Following early DMC meetings, it was reported that the number of babies admitted to a neonatal unit within 48 hours for at least 48 exceeded the number anticipated in the sample size calculation by an order of magnitude. Following a review of cases by the blinded review panel at the end of the trial, the number still exceeded that anticipated in the sample size calculation, though to a lesser degree. Hence this component far outweighs the other rarer components; stillbirth, neonatal death and moderate or severe neonatal encephalopathy, and will strongly influence the composite primary outcome. To explore the impact of this on the main findings, a sensitivity analysis of the primary composite outcome will be performed; including only the most severely affected babies admitted to a neonatal unit and allocated score of ≥7 on the Primary Outcome Review Scoring Sheet by the blinded review panel.

In addition, a sensitivity analysis will be undertaken related to the timing of the 2 year follow-up forms if a significant number are completed outside the proposed time window of 2 years ± 3 months of age. These forms will be excluded in a repeat analysis to investigate the robustness of the main findings to this eventuality.

4.17 Statistical Software Employed

Stata/SE version 13 for Windows for analysis.

4.18 Statistical Methods Used for Analysis of Secondary Outcomes

Generalised estimating equations (GEE), or a similar method, will be used for the analysis of secondary outcomes, adjusting for the stratification factors used in the randomisation procedure (centre and singleton/twin pregnancy). For Normally distributed continuous outcomes, we will present the mean and standard deviation for each group, calculating the mean difference plus 99% confidence interval using a Gaussian model with the identity link. For the length of labour, we will present the geometric mean ratio (GMR). For binary and categorical outcomes we will present counts and percentages for each group and calculate the relative risk with corresponding 99% confidence interval using a binomial or Poisson model with the log link. For the number of thumb entries per hour from trial entry to first yellow level of concern or full dilation, the rate ratio plus 99% confidence interval will be calculated using a Poisson model with the log link. For skewed continuous outcomes, we will present the median and interquartile range (or entire range, whichever appropriate) for each group, and compare the difference in medians between groups using quantile regression. We will not be able to adjust for the correlation among twins and siblings using this method.

4.19 Deviation from Analysis Described in Protocol

For the count variables, median differences were all zero with zero confidence intervals, though some were statistically significant and adjusted quantile regression models were not performing well (lack of convergence), so rate ratios are presented as the effect measure instead of median differences. Median {IQR} still presented for each variable as planned. Also, the hazard ratio is presented for the time from first red alert to birth instead of median difference, in keeping with the comparison of rates for the rest of the process outcomes. This was agreed at the INFANT Project Management Group meeting 23/10/2014.

The outcome time from first red level of concern to delivery was changed to time from last red level of concern to delivery. This was agreed at the INFANT Co-Investigator Group meeting 15/07/2015 following a review by Professor Steer. It was found that the first red level of concern was frequently an artefact and therefore did not accurately capture information relating to prompt action at or around delivery following a red alert.

4.20 References

1. Johnson A. Follow up studies: a case for a standard minimum data set. Arch Dis Child 1997; 76:F61-F63.
2. Disability and perinatal care: a report of two working groups convened by the National Perinatal Epidemiology Unit and the former Oxford Regional Health Authority (1994). Oxford, England: NPEU and ORHA.
3. Kahan BC, Morris TP. Reporting and analysis of trials using stratified randomisation in leading medical journals: review and reanalysis. British Medical Journal 2012; 345.
4. ONS. Birth Statistics Statistical Bulletin 2008. 2009.
5. Smith GCS, Pell JP, Dobbie R. Interpregnancy interval and risk of preterm birth and neonatal death: retrospective cohort study. British Medical Journal 2003; 327: 313-9.

6. ONS. Birth Statistics: Review of the National Statistician on births and patterns of family building in England and Wales, 2008. Series FM1 No 372009.
7. Yelland LN, Salter AB, Ryan P, Makrides M. Analysis of binary outcomes from randomised trials including multiple births: when should clustering be taken into account? Paediatric and Perinatal Epidemiology 2011; 25: 283-97.
8. Marston L, Peacock JL, Keming Y, et al. Comparing methods of analysing datasets with small clusters: case studies using four paediatric datasets. Paediatric and Perinatal Epidemiology 2009; 23: 380-92.
9. Schulz KF, Altman DG, Moher D, Group ftC. CONSORT 2010 Statement: updated guidelines for reporting parallel group randomised trials. British Medical Journal 2010; 340: 698-702.
10. Yelland LN, Salter AB, Ryan P. Relative risk estimation in randomized controlled trials: a comparison of methods for independent observations. The international journal of biostatistics 2011; 7:5.
11. Altman DG, Bland JM. Statistics notes: Interaction revisited: the difference between two estimates. British Medical Journal 2003; 326: 219.
12. Matthews JNS, Altman DG. Statistics notes: Interaction 3: How to examine heterogeneity. British Medical Journal 1996; 313: 862.

Annex

Classification of indications for instrumental vaginal delivery and caesarean section

Indications for instrumental vaginal delivery and caesarean section	Fetal distress	Failure to progress	Other
Abnormal CTG	Yes		
Abnormal FBS ph	Yes		
Abnormal presentation or lie		Yes	
Ante Partum haemorrhage	Yes		
APH Intrapartum haemorrhage	Yes		
Breech presentation		Yes	
Cephalopelvic disproportion		Yes	
Chorioamnionitis	Yes		
Cord prolapse or presentation	Yes		
Delay in 1st stage		Yes	
Delay in 2nd stage		Yes	
Eclampsia			Yes
Failed induction		Yes	
Failed forceps/ventouse		Yes	
Failed trial of forceps		Yes	
Failed trial of ventouse		Yes	
Failure to progress		Yes	
Fetal compromise - abnormal FCTG	Yes		
Fetal compromise - meconium stained liquor	Yes		
Fetal compromise suspected or indicated	Yes		
Fetal reason	Yes		
HELLP			Yes
Intrapartum haemorrhage	Yes		
IUGR	Yes		
Low scalp Ph	Yes		
Malpresentation		Yes	
Malpresentation/unstable lie		Yes	
Maternal condition			Yes
Maternal distress/exhaustion		Yes	
Maternal effort contraindicated			Yes
Maternal hypertension			Yes
Maternal medical condition			Yes
Maternal medical disease			Yes
Maternal request			Yes
Multiple pregnancy			Yes
Non-reassuring CTG	Yes		
Non-reassuring FBS	Yes		
Obstructed twin/triplet		Yes	
Other			Yes
Other (fetal)	Yes		

Indications for instrumental vaginal delivery and caesarean section	Fetal distress	Failure to progress	Other
Other (maternal)		Yes	
Other maternal medical history			Yes
Placenta abruption	Yes		
Placenta praevia			Yes
Planned as elective			Yes
Pre-eclampsia			Yes
Presumed fetal compromise	Yes		
Previous caesarean section			Yes
Previous infertility			Yes
Previous lower segment CS			Yes
Previous poor obstetric outcome			Yes
Previous obstetric history			Yes
Previous traumatic vaginal delivery			Yes
Previous uterine surgery			Yes
Prolonged 2nd stage			
Pyrexia in labour	Yes		
Ruptured uterus	Yes		
Slow progress in 1st stage		Yes	
Slow progress in 2nd stage		Yes	
Suspected fetal distress	Yes		
Unstable lie		Yes	

Additional tables and figures

Table S1: List of participating centres

	Decision support (n=22,987) n (%)	No decision support n=(23,055) n (%)
Birmingham Women's	1,131 (4.9)	1,131 (4.9)
Burnley General	2,058 (9.0)	2,062 (8.9)
Chelsea and Westminster, London	441 (1.9)	449 (2.0)
Coventry	600 (2.6)	607 (2.6)
Derby	421 (1.8)	425 (1.8)
Derriford, Plymouth	1,641 (7.1)	1,626 (7.1)
Homerton, London	944 (4.1)	940 (4.1)
Liverpool Women's	1,524 (6.6)	1,559 (6.8)
North Staffordshire	1,670 (7.3)	1,679 (7.3)
Northwick Park, London	926 (4.0)	936 (4.1)
Nottingham City & Queens Medical Centre	902 (3.9)	903 (3.9)
Princess Anne, Southampton	585 (2.5)	581 (2.5)
Princess Royal & Southern General, Glasgow	2,041 (8.9)	2,033 (8.8)
Queen Alexandra, Portsmouth	1,370 (6.0)	1,372 (6.0)
Rotunda, Dublin	1,735 (7.6)	1,728 (7.5)
Royal Bolton	499 (2.2)	510 (2.2)
St Mary's, Manchester	935 (4.1)	945 (4.1)
Stoke Mandeville	1,214 (5.3)	1,216 (5.3)
UCLH	196 (0.9)	192 (0.8)
Warrington	1,730 (7.5)	1,732 (7.5)
Warwick	424 (1.8)	429 (1.9)

Table S2: Reasons for withdrawal

	Decision support (n=23)	No decision support (n=7)
No longer wishes data to be collected/used	2	2
Did not like alerts	4	0
Twin baby died	1	0
No longer wishes to be followed up	1	0
Not happy with care/approach	1	2
Too distressed	1	0
Not happy with allocation	4	0
Not known/other	9	3

Table S3: Reasons for randomised in error

	Decision support (n=515)	No decision support (n=544)
No labour/elective caesarean	378	371
Date of randomisation after date of delivery	92	120
Fetal abnormality	18	19
Gestational age at entry <35 weeks or not known	10	15
Not CTG monitored	9	9
Not known/other	8	10

Table S4: Sensitivity analysis of composite primary outcome (panel review score ≥7 versus ≥3)

	**Decision support (n=23,263)**	**No decision support (n=23,351)**	**Adjusted[a] relative risk (95% CI)**
Composite primary outcome (1)-(4), n (%):[b]<br>*Unknown*	28 (0.1)<br>*1*	29 (0.1)<br>*2*	0.97 (0.58 to 1.63)
(1) Intrapartum stillbirths, n (%):[c]	1 (-)	2 (-)	0.50 (0.05 to 5.53)
(2) Neonatal deaths up to 28 days after birth, n (%):[d]	6 (-)	4 (-)	1.51 (0.42 to 5.33)
(3) Neonatal encephalopathy (requiring cooling), n (%):	18 (0.1)	21 (0.1)	0.86 (0.46 to 1.61)
(4) Admission to neonatal unit within 48 hours of birth for ≥ 48 hours due to feeding difficulties, respiratory illness/symptoms or encephalopathy and evidence of compromise at birth with panel review score ≥7, n (%):	3 (-)	2 (-)	1.51 (0.25 to 9.01)

[a] Adjusted for twin birth and clustering due to twins and multiple birth episodes. Minimisation factors not adjusted for due to small number of events.

[b] The components of the primary outcome are mutually exclusive and outcomes listed higher take precedence over those listed lower down, e.g. if a baby with neonatal encephalopathy died within 28 days, the outcome would be recorded as neonatal death.

[c] Excluding stillbirths due to congenital anomalies.

[d] Excluding deaths due to congenital anomalies. Deaths after hospital discharge not reported for the Republic of Ireland.

Table S5: Quality of care outcomes

**Levels of care[a]**	**Decision support**	**No decision support**	**Adjusted[b] Risk ratio (99% CI)**
**Babies with an adverse outcome[c]**	**(n=35)**	**(n= 36)**	
Babies with an adverse outcome where care has been judged not to be suboptimal (level 0):	11 (31.4)	15 (41.7)	
Babies with an adverse outcome where care has been judged to be suboptimal (levels 1,2 and 3):	24 (68.6)	21 (58.3)	1.18 (0.82 to 1.68)
Level 1	7 (20.)	2 (5.6)	
Level 2	3 (8.6)	6 (16.7)	
Level 3	14 (40.0)	13 (36.1)	

[a] Level 0 - No suboptimal care; Level 1 - suboptimal care, but different management would have made no difference to outcome; Level 2 - suboptimal care, and different management might have made a difference to outcome; Level 3 - suboptimal care, and different management would reasonably be expected to have made a difference to the outcome.
[b] Adjusted for twin birth and clustering due to twins and multiple birth episodes. Minimisation factors not adjusted for due to small number of events.
[c] Neonatal death, stillbirth or trial primary outcome with metabolic acidosis (cord artery pH <7.05 with base deficit ≥ 12 mmol/l).

Table S6: Quality of care outcomes (all babies in denominator)

Levels of care[a]	Decision support	No decision support	Adjusted[b] Relative risk (99% CI)
**All babies**	**(n=23,263)**	**(n=23,351)**	
Babies with an adverse outcome[c] where care has been judged not to be suboptimal (level 0):	11 (0.05)	15 (0.06)	
Babies with an adverse outcome where care has been judged to be suboptimal (levels 1,2 and 3):	24 (0.1)	21 (0.09)	1.15 (0.64 to 2.06)
Level 1	7 (0.03)	2 (0.01)	
Level 2	3 (0.01)	6 (0.03)	
Level 3	14 (0.06)	13 (0.06)	

[a] Level 0 - No suboptimal care; Level 1 - suboptimal care, but different management would have made no difference to outcome; Level 2 - suboptimal care, and different management might have made a difference to outcome; Level 3 - suboptimal care, and different management would reasonably be expected to have made a difference to the outcome.

[b] Adjusted for twin birth and clustering due to twins and multiple birth episodes. Minimisation factors not adjusted for due to small number of events.

[c] Neonatal death, stillbirth or trial primary outcome with metabolic acidosis (cord artery pH <7.05 with base deficit ≥ 12 mmol/l).

Table S7: Number of levels of concern after trial entry (all babies in denominator)

	Decision support (n=22,987)	No decision support[a] (n=23,055)	Adjusted[b] effect measure (99% CI)
No labour	*378*	*371*	
Date and time of randomisation after date and time of delivery	*92*	*120*	
	**(n=22,517)**	**(n=22,564)**	**Risk ratios:**
Number of blue, yellow or red levels of concern:			**Rate ratios:**
Median {IQR}	9 {5, 14}	9 {5, 15}	
Rate per hour	1.35	1.38	0.98 (0.96 to 1.00)
Unknown[c]	*871*	*924*	
Number of blue levels of concern:			
Median {IQR}	7 {4, 11}	7 {4, 11}	
Rate per hour	1.04	1.03	1.01 (1.00 to 1.03)
Unknown[c]	*871*	*924*	
Number of yellow levels of concern:			
Median {IQR}	1 {0, 3}	2 {0, 4}	
Rate per hour	0.29	0.33	0.87 (0.85 to 0.90)
Unknown[c]	*871*	*924*	
Number of red levels of concern:			
Median {IQR}	0 {0, 0}	0 {0, 0}	
Rate per hour	0.02	0.02	0.96 (0.88 to 1.04)
Unknown[c,d]	*1,693*	*1,757*	

[a] Level 0 - No suboptimal care; Level 1 - suboptimal care, but different management would have made no difference to outcome; Level 2 - suboptimal care, and different management might have made a difference to outcome; Level 3 - suboptimal care, and different management would reasonably be expected to have made a difference to the outcome.

[b] Adjusted for twin birth and clustering due to twins and multiple birth episodes. Minimisation factors not adjusted for due to small number of events.

[c] Neonatal death, stillbirth or trial primary outcome with metabolic acidosis (cord artery pH <7.05 with base deficit ≥ 12 mmol/l).

Table S8: Maternal characteristics at trial entry by follow-up status: responders versus non responders or not followed up at 2 years (mothers of surviving infants without the trial primary outcome only)

	Non responders or not followed up at 2 years (n=38,669)[a]		Responders at 2 years (n=6,986)[a]		P-value[b]
Maternal age (years):					
Median {IQR}	29	{24, 33}	30	{26, 34}	<0.001
Ethnic group, n (%):[c]					
White	28,714	(81.9)	5,461	(90.9)	
Indian	1,318	(3.8)	130	(2.2)	
Pakistani	1,356	(3.9)	166	(2.8)	
Bangladeshi	190	(0.5)	19	(0.3)	<0.001
Black Caribbean	231	(0.7)	19	(0.3)	
Black African	917	(2.6)	37	(0.6)	
Any other ethnic group	2,354	(6.7)	176	(2.9)	
Unknown	*3,589*		*978*		
Twin pregnancy, n(%):	486	(1.3)	80	(1.2)	0.44
Gestational age at entry (completed weeks):					
Median {IQR}	40	{38, 41}	40	{39, 41}	<0.001
$<35^{+0}$	9	(-)	1	(-)	
35^{+0} to 37^{+6}	4,314	(11.2)	682	(9.8)	
38^{+0} to 39^{+6}	12,467	(32.3)	2,035	(29.1)	
40^{+0} to 41^{+6}	19,584	(50.7)	3,702	(53.0)	
$\geq 42^{+0}$	2,282	(5.9)	566	(8.1)	
BMI (at booking visit):					
Median {IQR}	25	{22, 30}	25	{22, 29}	0.64
<18.5	644	(2.6)	110	(2.2)	
18.5 to 24.9	10,321	(41.8)	2,125	(42.3)	
25 to 29.9	7,467	(30.2)	1,563	(31.1)	
30 to 34.9	3,638	(14.7)	735	(14.6)	
35 to 39.9	1,703	(6.9)	319	(6.4)	
≥40	936	(3.8)	169	(3.4)	
Unknown	*13,960*		*1,965*		
Smoking (at booking visit), n (%):					
Yes	4,198	(15.0)	747	(11.9)	
No	23,681	(85.0)	5,518	(88.1)	<0.001
Unknown	*10,795*		*721*		
Parity, n (%):					
Nulliparous	22,792	(59.0)	4,317	(61.8)	
Parous	15,858	(41.0)	2,669	(38.2)	<0.001
Obstetric history, n (%):					
Previous stillbirth	425	(1.1)	70	(1.0)	0.47
Previous elective Caesarean section	359	(0.9)	100	(1.4)	<0.001
Previous emergency Caesarean section	2,028	(5.2)	413	(5.9)	0.02
Previous neonatal death	153	(0.4)	19	(0.3)	0.12
Cervical dilatation at time of trial entry (cm):					
Median {IQR}	4 {2, 6}		4 {2, 5}		0.09
Unknown	*27,528*		*4,750*		
Fetal growth restriction suspected at labour onset, n (%):	1,506	(3.9)	247	(3.5)	0.08
Labour induction, n (%):					
Induced	22,848	(59.5)	4,022	(57.9)	
Spontaneous	14,932	(38.9)	2,829	(40.7)	0.01
No labour	632	(1.7)	101	(1.5)	
Epidural analgesia, n (%):					
Yes	4,966	(28.0)	425	(15.9)	
No	12,798	(72.0)	2,257	(84.2)	<0.001
Unknown[d]	*20,905*		*4,304*		
Presence of meconium, n (%):					
Yes	771	(4.5)	113	(4.1)	
No	16,202	(95.5)	2,642	(95.9)	0.30
Unknown[d]	*21,696*		*4,231*		

Missing data are <1% unless otherwise presented; there were no apparent differences in missing data between trial arms.

[a] Women with more than one birth episode in the study period are included more than once.

[b] P-value from chi-square test for categorical variables and Wilcoxon rank-sum test for continuous variables.

[c] As coded by the NHS.

[d] Timing of epidural and presence of meconium in relation to trial entry only collected from 2013 for most centres.

Table S9: Maternal characteristics at trial entry by follow-up status: responders versus non responders at 2 years (mothers of surviving infants without the trial primary outcome only)

	Non responders at 2 years (n=5,560)[a]		Responders at 2 years (n=6,986)[a]		P-value[b]
Maternal age (years):					
Median {IQR}	27	{23, 31}	30	{26, 34}	<0.001
Ethnic group, n (%):[c]					
White	4,094	(86.8)	5,461	(90.9)	
Indian	140	(3.0)	130	(2.2)	
Pakistani	230	(4.9)	166	(2.8)	<0.001
Bangladeshi	18	(0.4)	19	(0.3)	
Black Caribbean	15	(0.3)	19	(0.3)	
Black African	54	(1.1)	37	(0.6)	
Any other ethnic group	167	(3.5)	176	(2.9)	
Unknown	*842*		*978*		
Twin pregnancy, n(%):	78	(1.4)	80	(1.2)	0.20
Gestational age at entry (completed weeks):					
Median {IQR}	40	{38, 41}	40	{39, 41}	<0.001
$<35^{+0}$	2	(-)	1	(-)	
35^{+0} to 37^{+6}	665	(12.0)	682	(9.8)	
38^{+0} to 39^{+6}	1,728	(31.1)	2,035	(29.1)	
40^{+0} to 41^{+6}	2,787	(50.1)	3,702	(53.0)	
$\geq 42^{+0}$	377	(6.8)	566	(8.1)	
BMI (at booking visit):					
Median {IQR}	26	{22, 30}	25	{22, 29}	0.01
<18.5	130	(3.3)	110	(2.2)	
18.5 to 24.9	1,559	(39.1)	2,125	(42.3)	
25 to 29.9	1,187	(29.8)	1,563	(31.1)	
30 to 34.9	607	(15.2)	735	(14.6)	
35 to 39.9	313	(7.9)	319	(6.4)	
≥40	192	(4.8)	169	(3.4)	
Unknown	*1,572*		*1,965*		
Smoking (at booking visit), n (%):					
Yes	994	(20.7)	747	(11.9)	<0.001
No	3,807	(79.3)	5,518	(88.1)	
Unknown	*759*		*721*		
Parity, n (%):					
Nulliparous	3,043	(54.7)	4,317	(61.8)	<0.001
Parous	2,517	(45.3)	2,669	(38.2)	
Obstetric history, n (%):					
Previous stillbirth	77	(1.4)	70	(1.0)	0.05
Previous elective Caesarean section	61	(1.1)	100	(1.4)	0.10
Previous emergency Caesarean section	345	(6.2)	413	(5.9)	0.49
Previous neonatal death	22	(0.4)	19	(0.3)	0.23
Cervical dilatation at time of trial entry (cm):					
Median {IQR}	4 {2, 5}		4 {2, 5}		0.53
Unknown	*3,730*		*4,750*		
Fetal growth restriction suspected at labour onset, n (%):	266	(4.8)	247	(3.5)	<0.001
Labour induction, n (%):					
Induced	3,108	(56.2)	4,022	(57.9)	
Spontaneous	2,336	(42.2)	2,829	(40.7)	0.18
No labour	86	(1.6)	101	(1.5)	
Epidural analgesia, n (%):					
Yes	266	(13.8)	425	(15.9)	0.05
No	1,665	(86.2)	2,257	(84.2)	
Unknown[d]	*3,629*		*4,304*		
Presence of meconium, n (%):					
Yes	66	(3.1)	113	(4.1)	0.06
No	2,085	(96.9)	2,642	(95.9)	
Unknown[d]	*3,409*		*4,231*		

Missing data are <1% unless otherwise presented; there were no apparent differences in missing data between trial arms.

[a] Women with more than one birth episode in the study period are included more than once.

[b] P-value from chi-square for categorical variables and Wilcoxon rank-sum test for continuous variables.

[c] As coded by NHS.

[d] Timing of epidural and presence of meconium in relation to trial entry only collected from 2013 for most centres.

Table S10: Components of non-major and major disability at 2 years

Disability component	Decision support (n=3,556)		No Decision support (n=3,510)	
Cognition, n (%):				
None	3,159	(93.4)	3,134	(94.2)
Non-major	180	(5.3)	143	(4.3)
Major	42	(1.2)	49	(1.5)
Unknown	*175*		*184*	
Communication, n (%):				
None	3,132	(88.6)	3,096	(88.7)
Non-major	394	(11.2)	392	(11.2)
Major	8	(0.2)	4	(0.1)
Unknown	*22*		*18*	
Physical ability, n (%):				
None	3,161	(92.3)	3,166	(93.7)
Non-major	245	(7.2)	199	(5.9)
Major	19	(0.6)	15	(0.4)
Unknown	*131*		*130*	
Vision, n (%):				
None	3,409	(99.6)	3,373	(99.7)
Non-major	14	(0.4)	8	(0.2)
Major	1	(-)	1	(-)
Unknown	*132*		*128*	
Hearing, n (%):				
None	3,307	(99.0)	3,293	(99.2)
Non-major	30	(0.9)	25	(0.8)
Major	5	(0.2)	2	(0.1)
Unknown	*214*		*190*	
Growth, n (%):				
None	1,899	(90.5)	1,882	(92.2)
Non-major	137	(6.5)	102	(5.0)
Major	63	(3.0)	58	(2.8)
Unknown	*1,457*		*1,468*	
Seizures, n (%):				
None	3,302	(99.0)	3,259	(98.9)
Non-major	31	(0.9)	30	(0.9)
Major	4	(0.1)	5	(0.2)
Unknown	*219*		*216*	
Feeding, n (%):				
None	3,385	(99.9)	3,326	(99.9)
Non-major	0	(0.0)	0	(0.0)
Major	3	(0.1)	5	(0.2)
Unknown	*168*		*179*	
Respiratory, n (%):				
None	3,284	(97.0)	3,266	(97.4)
Non-major	100	(3.0)	86	(2.6)
Major	0	(0.0)	0	(0.0)
Unknown	*172*		*158*	
Other disabiliy, n (%):				
None	3,550	(99.9)	3,501	(99.8)
Non-major	0	(0.0)	0	(0.0)
Major	3	(0.1)	6	(0.2)
Unknown	*3*		*3*	

Subgroup analyses

NOTE: Subgroup analyses are not adjusted for the stratification factors used at randomisation due to the small number of events in some subgroup categories.

Figure S1: Maternal and neonatal outcomes by twin pregnancy

Subgroup analysis: Twins

Outcome	Decision support (No of events/total no)	(%)	No decision support (No of events/total no)	(%)	Risk ratio (99% CI) unless otherwise specified	P Value
**Composite primary outcome (95% CI)**						
Singleton	166/22710	(0.7)	162/22758	(0.7)	1.03 (0.83, 1.27)	0.51
Twin	6/552	(1.1)	9/591	(1.5)	0.71 (0.26, 1.99)	
**Instrumental delivery or caesarean section**						
Singleton	11096/22711	(48.9)	11001/22759	(48.3)	1.01 (0.99, 1.04)	0.17
Twin	271/552	(49.1)	319/592	(53.9)	0.91 (0.75, 1.10)	
**Caesarean section**						
Singleton	5517/22711	(24.3)	5382/22759	(23.6)	1.03 (0.98, 1.07)	0.51
Twin	152/552	(27.5)	173/592	(29.2)	0.94 (0.67, 1.32)	
**Admission to a higher level of care**						
Singleton	1310/22697	(5.8)	1326/22747	(5.8)	0.99 (0.90, 1.09)	0.31
Twin	78/550	(14.2)	101/591	(17.1)	0.83 (0.54, 1.29)	
**Apgar score <4 at 5 mins**						
Singleton	42/22705	(0.2)	59/22749	(0.3)	0.71 (0.42, 1.20)	0.22
Twin	1/550	(0.2)	6/592	(1.0)	0.18 (0.01, 3.08)	
**Cord artery pH <7.15**						
Singleton	1593/14075	(11.3)	1634/13980	(11.7)	0.97 (0.89, 1.05)	0.02
Twin	32/359	(8.9)	61/390	(15.6)	0.57 (0.32, 1.00)	
**Metabolic acidosis**						
Singleton	144/13347	(1.1)	122/13289	(0.9)	1.18 (0.86, 1.61)	0.15
Twin	4/339	(1.2)	9/375	(2.4)	0.49 (0.11, 2.27)	
**Resuscitation**						
Singleton	2604/20660	(12.6)	2526/20712	(12.2)	1.03 (0.97, 1.11)	0.15
Twin	89/490	(18.2)	114/533	(21.4)	0.85 (0.60, 1.20)	
**Seizures whilst in hospital**						
Singleton	39/22632	(0.2)	39/22672	(0.2)	1.00 (0.56, 1.79)	
Twin	0/547	(0.0)	2/586	(0.3)	Not estimable	
**Baby destination not ward or home**						
Singleton	930/22499	(4.1)	905/22551	(4.0)	1.03 (0.92, 1.16)	0.77
Twin	74/543	(13.6)	82/585	(14.0)	0.97 (0.59, 1.60)	
**Median length of hospital stay to discharge (days)[a]**						
Singleton					0.99 (0.97, 1.01)	0.22
Twin					1.06 (0.89, 1.26)	

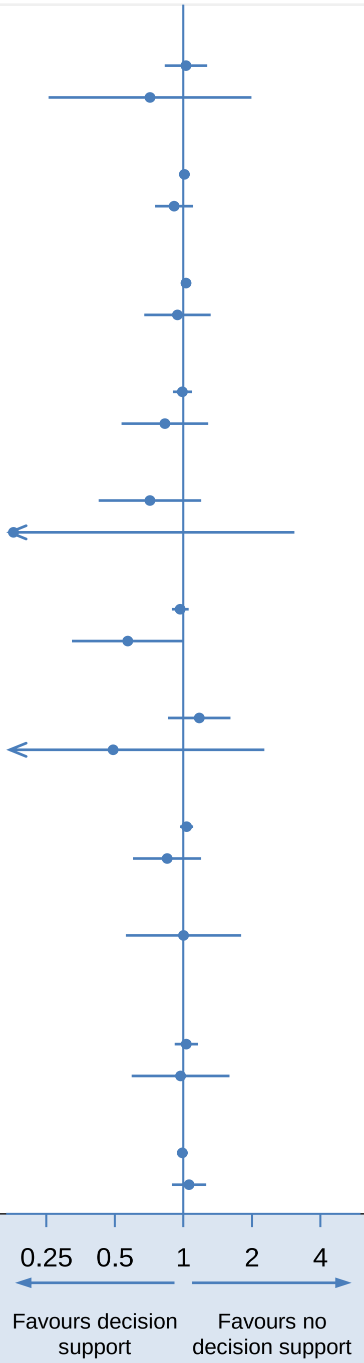

[a] Hazard ratios reported

Figure S2: Maternal and neonatal outcomes by suspected fetal growth restriction

Subgroup analysis: Suspected fetal growth restriction

Outcome	Decision support		No decision support		Risk ratio (99% CI) unless otherwise specified	P Value
	No of events/total no (%)					
**Composite primary outcome (95% CI)**						
No FGR	166/22384	(0.7)	164/22407	(0.7)	1.01 (0.82, 1.26)	0.87
FGR	6/878	(0.7)	7/942	(0.7)	0.92 (0.31, 2.73)	
**Instrumental delivery or caesarean section**						
No FGR	11085/22385	(49.5)	11051/22409	(49.3)	1.00 (0.98, 1.03)	0.12
FGR	282/878	(32.1)	269/942	(28.6)	1.12 (0.93, 1.36)	
**Caesarean section**						
No FGR	5530/22385	(24.7)	5432/22409	(24.2)	1.02 (0.98, 1.06)	0.15
FGR	139/878	(15.8)	123/942	(13.1)	1.21 (0.89, 1.65)	
**Admission to a higher level of care**						
No FGR	1307/22370	(5.8)	1330/22401	(5.9)	0.98 (0.89, 1.09)	0.52
FGR	81/877	(9.2)	97/937	(10.4)	0.89 (0.61, 1.31)	
**Apgar score <4 at 5 mins**						
No FGR	42/22377	(0.2)	62/22399	(0.3)	0.68 (0.40, 1.14)	0.58
FGR	1/878	(0.1)	3/942	(0.3)	0.36 (0.02, 6.99)	
**Cord artery pH <7.15**						
No FGR	1586/13938	(11.4)	1651/13882	(11.9)	0.96 (0.88, 1.04)	0.66
FGR	39/496	(7.9)	44/488	(9.0)	0.87 (0.51, 1.50)	
**Metabolic acidosis**						
No FGR	144/13205	(1.1)	129/13188	(1.0)	1.11 (0.82, 1.52)	0.51
FGR	4/481	(0.8)	2/476	(0.4)	1.98 (0.21, 18.33)	
**Resuscitation**						
No FGR	2603/20299	(12.8)	2546/20336	(12.5)	1.02 (0.96, 1.10)	0.99
FGR	90/851	(10.6)	94/909	(10.3)	1.02 (0.71, 1.48)	
**Seizures whilst in hospital**						
No FGR	36/22306	(0.2)	39/22320	(0.2)	0.92 (0.51, 1.67)	0.55
FGR	3/873	(0.3)	2/938	(0.2)	1.61 (0.15, 16.88)	
**Baby destination not ward or home**						
No FGR	946/22164	(4.3)	918/22196	(4.1)	1.03 (0.92, 1.16)	0.46
FGR	58/878	(6.6)	69/940	(7.3)	0.90 (0.57, 1.42)	
**Median length of hospital stay to discharge (days)[a]**						
No FGR					0.99 (0.97, 1.01)	0.79
FGR					0.98 (0.88, 1.09)	

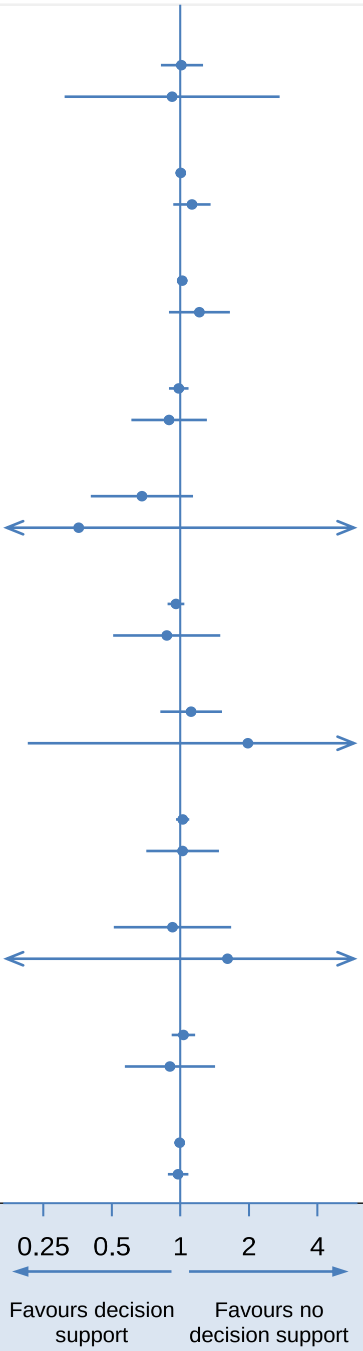

[a] Hazard ratios reported

Figure S3: Maternal and neonatal outcomes by body mass index at booking visit

Subgroup analysis: BMI at booking visit

Outcome	Decision support (No of events/total no)	(%)	No decision support (No of events/total no)	(%)	Risk ratio (99% CI) unless otherwise specified	P Value
**Composite primary outcome (95% CI)**						
12 to 18.5	4/381	(1.0)	3/389	(0.8)	1.36 (0.31, 6.04)	
18.5 to 24.9	34/6389	(0.5)	36/6319	(0.6)	0.93 (0.59, 1.49)	
25 to 29.9	22/4581	(0.5)	29/4622	(0.6)	0.77 (0.44, 1.33)	0.81
30 to 70	34/3803	(0.9)	30/3844	(0.8)	1.15 (0.70, 1.87)	
Unrecorded	78/8108	(1.0)	73/8175	(0.9)	1.08 (0.78, 1.48)	
**Instrumental delivery or caesarean section**						
12 to 18.5	155/381	(40.7)	164/389	(42.2)	0.96 (0.77, 1.21)	
18.5 to 24.9	3268/6389	(51.2)	3171/6320	(50.2)	1.02 (0.97, 1.07)	
25 to 29.9	2293/4581	(50.1)	2355/4623	(50.9)	0.98 (0.93, 1.04)	0.20
30 to 70	1750/3804	(46.0)	1816/3844	(47.2)	0.97 (0.91, 1.04)	
Unrecorded	3901/8108	(48.1)	3814/8175	(46.7)	1.03 (0.99, 1.08)	
**Caesarean section**						
12 to 18.5	50/381	(13.1)	53/389	(13.6)	0.96 (0.60, 1.55)	
18.5 to 24.9	1443/6389	(22.6)	1347/6320	(21.3)	1.06 (0.97, 1.16)	
25 to 29.9	1242/4581	(27.1)	1217/4623	(26.3)	1.03 (0.94, 1.13)	0.74
30 to 70	1101/3804	(28.9)	1122/3844	(29.2)	0.99 (0.90, 1.09)	
Unrecorded	1833/8108	(22.6)	1816/8175	(22.2)	1.02 (0.94, 1.10)	
**Admission to a higher level of care**						
12 to 18.5	22/381	(5.8)	26/389	(6.7)	0.86 (0.41, 1.80)	
18.5 to 24.9	345/6385	(5.4)	338/6317	(5.4)	1.01 (0.83, 1.23)	
25 to 29.9	251/4579	(5.5)	284/4619	(6.1)	0.89 (0.71, 1.11)	0.48
30 to 70	222/3798	(5.8)	249/3839	(6.5)	0.90 (0.71, 1.14)	
Unrecorded	548/8104	(6.8)	530/8174	(6.5)	1.04 (0.89, 1.22)	
**Apgar score <4 at 5 mins**						
12 to 18.5	4/381	(1.0)	1/389	(0.3)	4.08 (0.23, 72.45)	
18.5 to 24.9	13/6387	(0.2)	20/6319	(0.3)	0.64 (0.25, 1.64)	
25 to 29.9	6/4581	(0.1)	10/4623	(0.2)	0.61 (0.16, 2.29)	0.56
30 to 70	6/3804	(0.2)	12/3841	(0.3)	0.50 (0.14, 1.83)	
Unrecorded	14/8102	(0.2)	22/8169	(0.3)	0.64 (0.27, 1.55)	
**Cord artery pH <7.15**						
12 to 18.5	19/197	(9.6)	33/236	(14.0)	0.69 (0.34, 1.39)	
18.5 to 24.9	401/3823	(10.5)	426/3697	(11.5)	0.91 (0.77, 1.08)	
25 to 29.9	300/2730	(11.0)	324/2810	(11.5)	0.95 (0.79, 1.16)	0.39
30 to 70	243/2358	(10.3)	266/2324	(11.4)	0.90 (0.73, 1.12)	
Unrecorded	662/5326	(12.4)	646/5303	(12.2)	1.02 (0.89, 1.17)	
**Metabolic acidosis**						
12 to 18.5	0/189	(0.0)	1/231	(0.4)	Not estimable	
18.5 to 24.9	32/3676	(0.9)	24/3548	(0.7)	1.29 (0.64, 2.57)	
25 to 29.9	32/2593	(1.2)	36/2692	(1.3)	0.92 (0.50, 1.72)	0.79
30 to 70	25/2245	(1.1)	21/2207	(1.0)	1.17 (0.55, 2.50)	
Unrecorded	59/4983	(1.2)	49/4986	(1.0)	1.20 (0.73, 1.98)	
**Resuscitation**						
12 to 18.5	54/369	(14.6)	38/383	(9.9)	1.47 (0.89, 2.46)	
18.5 to 24.9	721/6200	(11.6)	695/6149	(11.3)	1.03 (0.90, 1.17)	
25 to 29.9	562/4410	(12.7)	552/4443	(12.4)	1.03 (0.89, 1.19)	0.26
30 to 70	501/3623	(13.8)	532/3660	(14.5)	0.95 (0.82, 1.10)	
Unrecorded	855/6548	(13.1)	823/6610	(12.5)	1.05 (0.93, 1.18)	
**Seizures whilst in hospital**						
12 to 18.5	0/378	(0.0)	1/386	(0.3)	Not estimable	
18.5 to 24.9	9/6369	(0.1)	11/6305	(0.2)	0.81 (0.25, 2.58)	
25 to 29.9	9/4566	(0.2)	7/4598	(0.2)	1.29 (0.35, 4.74)	0.64
30 to 70	8/3795	(0.2)	5/3830	(0.1)	1.61 (0.37, 7.00)	
Unrecorded	13/8071	(0.2)	17/8139	(0.2)	0.77 (0.30, 1.99)	
**Baby destination not ward or home**						
12 to 18.5	15/378	(4.0)	13/386	(3.4)	1.18 (0.45, 3.08)	
18.5 to 24.9	201/6343	(3.2)	195/6289	(3.1)	1.02 (0.79, 1.33)	
25 to 29.9	128/4542	(2.8)	148/4585	(3.2)	0.87 (0.64, 1.19)	0.47
30 to 70	129/3775	(3.4)	140/3801	(3.7)	0.93 (0.68, 1.27)	
Unrecorded	531/8004	(6.6)	491/8075	(6.1)	1.09 (0.93, 1.28)	
**Median length of hospital stay to discharge (days)[a]**						
12 to 18.5					1.06 (0.91, 1.24)	
18.5 to 24.9					0.98 (0.95, 1.02)	
25 to 29.9					0.99 (0.95, 1.04)	0.28
30 to 70					1.03 (0.98, 1.08)	
Unrecorded					0.98 (0.95, 1.02)	

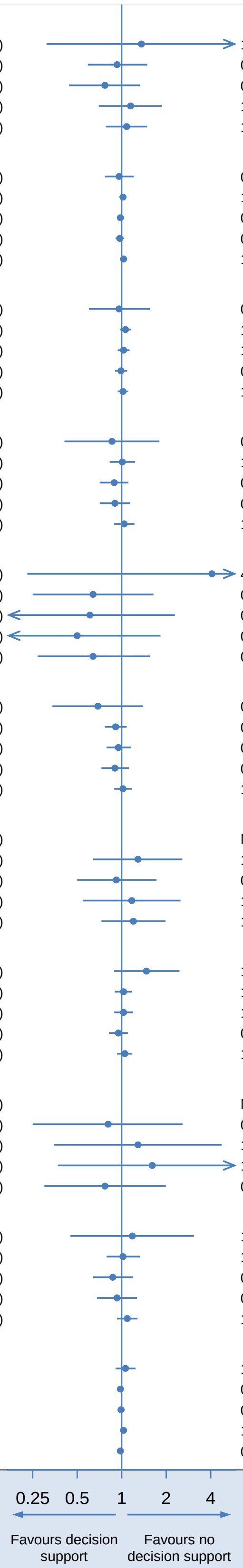

[a] Hazard ratios reported

Figure S4: Composite primary outcome by centre

Subgroup analysis: Centre

	Decision support		No decision support		Risk ratio (95% CI)	P Value
	No of events/total no (%)					
**Composite primary outcome**						
Birmingham Women's	15/1148	(1.3)	8/1149	(0.7)	1.87 (0.80, 4.41)	
Burnley General	17/2091	(0.8)	20/2097	(1.0)	0.85 (0.45, 1.62)	
Chelsea & Westminster, London	3/443	(0.7)	3/450	(0.7)	1.02 (0.21, 5.01)	
Coventry	3/606	(0.5)	3/612	(0.5)	1.01 (0.20, 4.98)	
Derby	2/427	(0.5)	1/433	(0.2)	2.03 (0.18, 22.28)	
Derriford, Plymouth	29/1665	(1.7)	30/1650	(1.8)	0.96 (0.58, 1.59)	
Homerton, London	10/957	(1.0)	10/951	(1.1)	0.99 (0.42, 2.38)	
Liverpool Women's	15/1549	(1.0)	11/1586	(0.7)	1.40 (0.64, 3.03)	
North Staffordshire	8/1682	(0.5)	5/1695	(0.3)	1.61 (0.53, 4.92)	
Northwick Park, London	8/935	(0.9)	12/944	(1.3)	0.67 (0.28, 1.64)	
Nottingham City & Queen's Medical Centre	4/918	(0.4)	1/920	(0.1)	4.01 (0.45, 35.80)	0.19
Princess Anne, Southampton	3/590	(0.5)	9/586	(1.5)	0.33 (0.09, 1.22)	
Princess Royal & Southern General, Glasgow	10/2054	(0.5)	4/2046	(0.2)	2.49 (0.78, 7.93)	
Queen Alexandra, Portsmouth	13/1393	(0.9)	10/1395	(0.7)	1.30 (0.57, 2.96)	
Rotunda, Dublin	3/1754	(0.2)	8/1751	(0.5)	0.37 (0.10, 1.41)	
Royal Bolton	8/502	(1.6)	2/514	(0.4)	4.09 (0.87, 19.19)	
St. Mary's, Manchester	7/953	(0.7)	5/963	(0.5)	1.41 (0.45, 4.44)	
Stoke Mandeville	5/1224	(0.4)	5/1230	(0.4)	1.00 (0.29, 3.46)	
UCLH	4/197	(2.0)	4/194	(2.1)	0.98 (0.25, 3.88)	
Warrington	2/1746	(0.1)	16/1750	(0.9)	0.13 (0.03, 0.54)	
Warwick	3/428	(0.7)	4/433	(0.9)	0.76 (0.17, 3.37)	
**Total** (unadjusted)	172/23262	(0.7)	171/23349	(0.7)	1.01 (0.82, 1.25)	

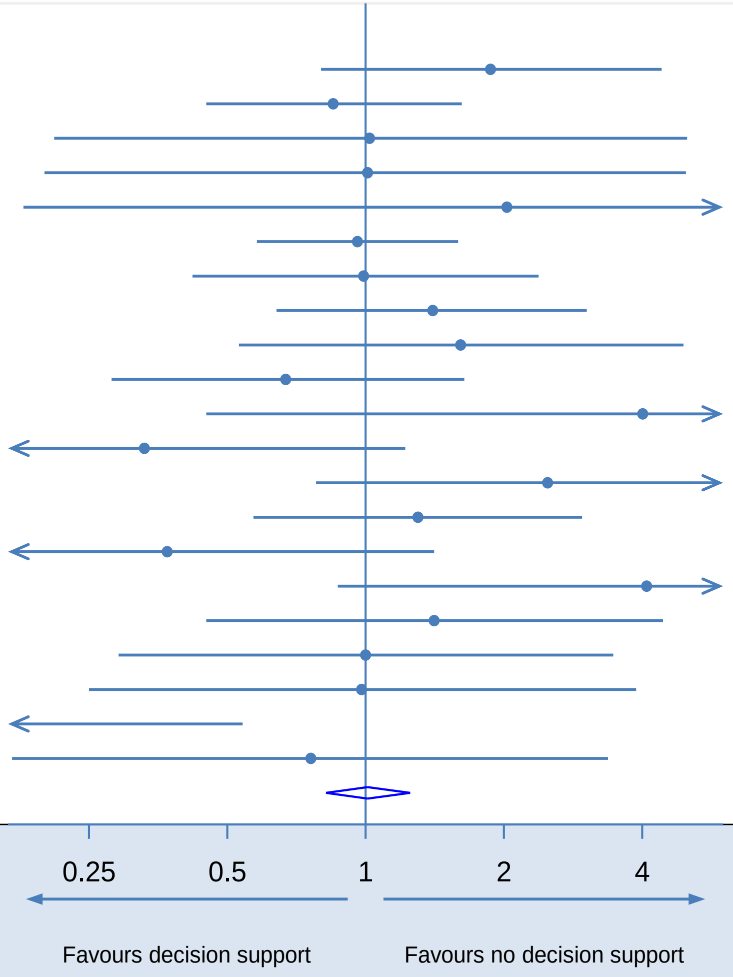

Figure S5: Instrumental delivery or caesarean section by centre

Subgroup analysis: Centre

	Decision support *No of events/total no (%)*		No decision support		Risk ratio (99% CI)	P Value
**Instrumental delivery or caesarean section**						
Birmingham Women's	630/1148	(54.9)	610/1149	(53.1)	1.03 (0.94, 1.14)	
Burnley General	1018/2091	(48.7)	1064/2097	(50.7)	0.96 (0.89, 1.04)	
Chelsea & Westminster, London	276/443	(62.3)	292/450	(64.9)	0.96 (0.84, 1.09)	
Coventry	311/606	(51.3)	328/612	(53.6)	0.96 (0.83, 1.10)	
Derby	201/427	(47.1)	206/433	(47.6)	0.99 (0.82, 1.19)	
Derriford, Plymouth	703/1665	(42.2)	664/1650	(40.2)	1.05 (0.94, 1.17)	
Homerton, London	542/957	(56.6)	522/951	(54.9)	1.03 (0.93, 1.15)	
Liverpool Women's	696/1549	(44.9)	703/1586	(44.3)	1.01 (0.91, 1.12)	
North Staffordshire	660/1682	(39.2)	688/1695	(40.6)	0.97 (0.87, 1.08)	
Northwick Park, London	578/935	(61.8)	569/944	(60.3)	1.03 (0.93, 1.13)	
Nottingham City & Queen's Medical Centre	419/918	(45.6)	437/920	(47.5)	0.96 (0.84, 1.09)	0.69
Princess Anne, Southampton	378/590	(64.1)	383/586	(65.4)	0.98 (0.88, 1.10)	
Princess Royal & Southern General, Glasgow	1015/2054	(49.4)	1001/2047	(48.9)	1.01 (0.93, 1.10)	
Queen Alexandra, Portsmouth	715/1393	(51.3)	684/1395	(49.0)	1.05 (0.95, 1.16)	
Rotunda, Dublin	902/1754	(51.4)	864/1752	(49.3)	1.04 (0.96, 1.14)	
Royal Bolton	258/502	(51.4)	249/514	(48.4)	1.06 (0.90, 1.25)	
St. Mary's, Manchester	496/953	(52.0)	461/963	(47.9)	1.09 (0.97, 1.22)	
Stoke Mandeville	677/1224	(55.3)	699/1230	(56.8)	0.97 (0.89, 1.07)	
UCLH	119/198	(60.1)	106/194	(54.6)	1.10 (0.88, 1.38)	
Warrington	552/1746	(31.6)	556/1750	(31.8)	1.00 (0.87, 1.13)	
Warwick	221/428	(51.6)	234/433	(54.0)	0.96 (0.81, 1.13)	
**Total** (unadjusted)	11367/23263	(48.9)	11320/23351	(48.5)	1.01 (0.98, 1.03)	

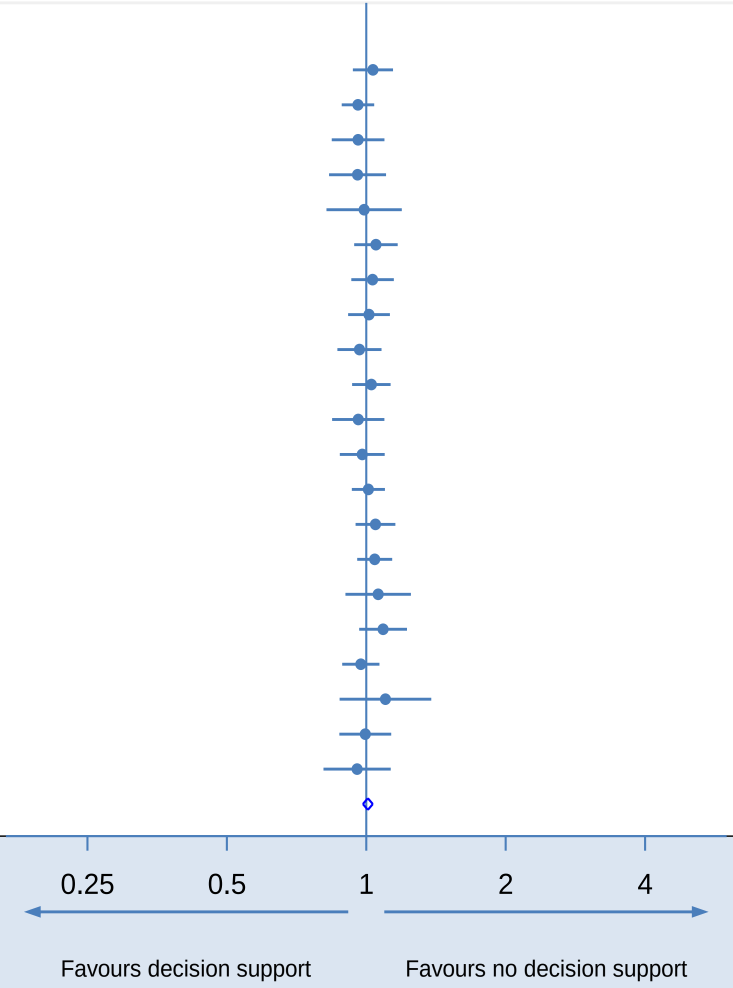

Figure S6: Caesarean section by centre

Subgroup analysis: Centre

	Decision support		No decision support		Risk ratio (99% CI)	P Value
	No of events/total no (%)					
**Caesarean section**						
Birmingham Women's	269/1148	(23.4)	268/1149	(23.3)	1.00 (0.82, 1.22)	
Burnley General	558/2091	(26.7)	551/2097	(26.3)	1.02 (0.89, 1.16)	
Chelsea & Westminster, London	125/443	(28.2)	132/450	(29.3)	0.96 (0.73, 1.26)	
Coventry	165/606	(27.2)	200/612	(32.7)	0.83 (0.66, 1.05)	
Derby	92/427	(21.5)	110/433	(25.4)	0.85 (0.61, 1.17)	
Derriford, Plymouth	301/1665	(18.1)	311/1650	(18.8)	0.96 (0.79, 1.16)	
Homerton, London	292/957	(30.5)	290/951	(30.5)	1.00 (0.84, 1.20)	
Liverpool Women's	324/1549	(20.9)	302/1586	(19.0)	1.10 (0.91, 1.33)	
North Staffordshire	355/1682	(21.1)	367/1695	(21.7)	0.97 (0.82, 1.16)	
Northwick Park, London	339/935	(36.3)	319/944	(33.8)	1.07 (0.91, 1.26)	
Nottingham City & Queen's Medical Centre	185/918	(20.2)	181/920	(19.7)	1.02 (0.80, 1.31)	0.26
Princess Anne, Southampton	190/590	(32.2)	208/586	(35.5)	0.91 (0.73, 1.12)	
Princess Royal & Southern General, Glasgow	568/2054	(27.7)	532/2047	(26.0)	1.06 (0.93, 1.22)	
Queen Alexandra, Portsmouth	453/1393	(32.5)	411/1395	(29.5)	1.10 (0.95, 1.28)	
Rotunda, Dublin	330/1754	(18.8)	286/1752	(16.3)	1.15 (0.95, 1.40)	
Royal Bolton	117/502	(23.3)	119/514	(23.2)	1.01 (0.75, 1.35)	
St. Mary's, Manchester	233/953	(24.4)	201/963	(20.9)	1.17 (0.94, 1.46)	
Stoke Mandeville	293/1224	(23.9)	292/1230	(23.7)	1.01 (0.84, 1.22)	
UCLH	57/198	(28.8)	41/194	(21.1)	1.36 (0.86, 2.16)	
Warrington	309/1746	(17.7)	313/1750	(17.9)	0.99 (0.82, 1.20)	
Warwick	114/428	(26.6)	121/433	(27.9)	0.95 (0.71, 1.27)	
**Total** (unadjusted)	5669/23263	(24.4)	5555/23351	(23.8)	1.02 (0.98, 1.07)	

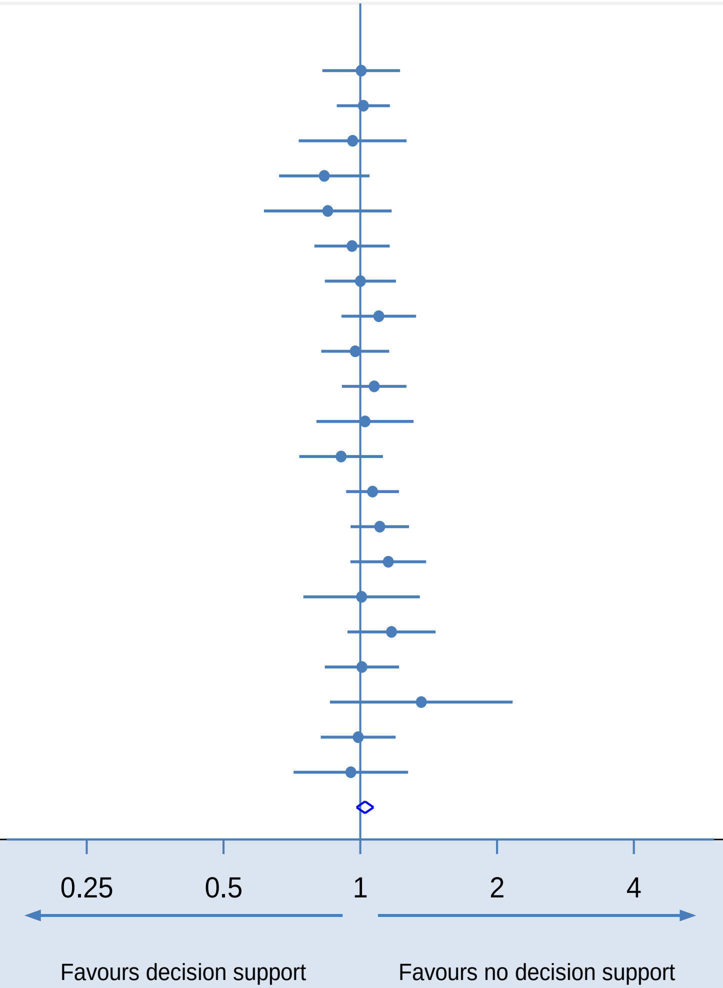

Figure S7: Admission of infant to a higher level of care by centre

Subgroup analysis: Centre

	Decision support		No decision support		Risk ratio (99% CI)	P Value
	No of events/total no (%)					
**Admission of infant to a higher level of care**						
Birmingham Women's	158/1148	(13.8)	128/1148	(11.1)	1.23 (0.92, 1.65)	
Burnley General	114/2086	(5.5)	118/2095	(5.6)	0.97 (0.69, 1.36)	
Chelsea & Westminster, London	24/443	(5.4)	26/450	(5.8)	0.94 (0.46, 1.93)	
Coventry	12/602	(2.0)	12/605	(2.0)	1.00 (0.34, 2.97)	
Derby	12/427	(2.8)	13/433	(3.0)	0.94 (0.34, 2.59)	
Derriford, Plymouth	89/1663	(5.4)	101/1650	(6.1)	0.87 (0.61, 1.26)	
Homerton, London	73/956	(7.6)	63/951	(6.6)	1.15 (0.75, 1.77)	
Liverpool Women's	108/1548	(7.0)	115/1585	(7.3)	0.96 (0.68, 1.35)	
North Staffordshire	67/1682	(4.0)	74/1695	(4.4)	0.91 (0.59, 1.41)	
Northwick Park, London	71/935	(7.6)	86/943	(9.1)	0.83 (0.56, 1.24)	
Nottingham City & Queen's Medical Centre	73/918	(8.0)	72/920	(7.8)	1.02 (0.67, 1.54)	0.45
Princess Anne, Southampton	32/588	(5.4)	51/586	(8.7)	0.63 (0.36, 1.10)	
Princess Royal & Southern General, Glasgow	105/2054	(5.1)	124/2047	(6.1)	0.84 (0.60, 1.18)	
Queen Alexandra, Portsmouth	67/1393	(4.8)	52/1395	(3.7)	1.29 (0.81, 2.06)	
Rotunda, Dublin	76/1753	(4.3)	71/1752	(4.1)	1.07 (0.70, 1.64)	
Royal Bolton	52/502	(10.4)	38/514	(7.4)	1.40 (0.82, 2.40)	
St. Mary's, Manchester	44/953	(4.6)	48/963	(5.0)	0.93 (0.54, 1.60)	
Stoke Mandeville	35/1224	(2.9)	35/1229	(2.8)	1.00 (0.54, 1.87)	
UCLH	35/198	(17.7)	33/194	(17.0)	1.04 (0.58, 1.86)	
Warrington	110/1746	(6.3)	133/1750	(7.6)	0.83 (0.60, 1.15)	
Warwick	31/428	(7.2)	34/433	(7.9)	0.92 (0.49, 1.72)	
**Total** (unadjusted)	1388/23247	(6.0)	1427/23338	(6.1)	0.98 (0.89, 1.07)	

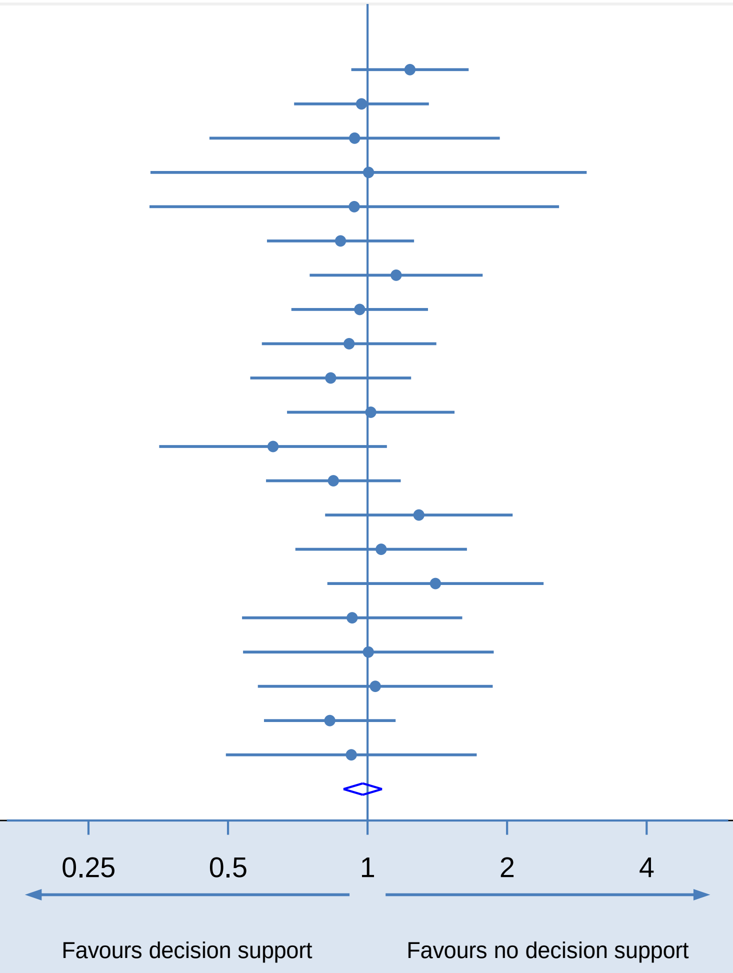

Figure S8: Apgar score by centre

Subgroup analysis: Centre

	Decision support No of events/total no	(%)	No decision support No of events/total no	(%)	Risk ratio (99% CI)	P Value[a]
**Apgar score <4 at 5 mins**						
Birmingham Women's	2/1147	(0.2)	1/1147	(0.1)	2.00 (0.09, 46.84)	
Burnley General	2/2091	(0.1)	6/2097	(0.3)	0.33 (0.04, 2.74)	
Chelsea & Westminster, London	0/443	(0.0)	1/450	(0.2)	Not estimable	
Coventry	1/602	(0.2)	1/609	(0.2)	1.01 (0.03, 38.59)	
Derby	0/427	(0.0)	1/433	(0.2)	Not estimable	
Derriford, Plymouth	3/1665	(0.2)	10/1650	(0.6)	0.30 (0.05, 1.62)	
Homerton, London	0/957	(0.0)	1/950	(0.1)	Not estimable	
Liverpool Women's	7/1549	(0.5)	4/1586	(0.3)	1.79 (0.36, 8.98)	
North Staffordshire	5/1682	(0.3)	4/1695	(0.2)	1.26 (0.22, 7.08)	
Northwick Park, London	1/935	(0.1)	3/944	(0.3)	0.34 (0.02, 6.58)	
Nottingham City & Queen's Medical Centre	0/917	(0.0)	2/920	(0.2)	Not estimable	0.80
Princess Anne, Southampton	1/590	(0.2)	3/586	(0.5)	0.33 (0.02, 6.46)	
Princess Royal & Southern General, Glasgow	4/2054	(0.2)	2/2047	(0.1)	1.99 (0.21, 18.53)	
Queen Alexandra, Portsmouth	5/1393	(0.4)	6/1395	(0.4)	0.83 (0.18, 3.96)	
Rotunda, Dublin	3/1754	(0.2)	3/1752	(0.2)	1.00 (0.12, 8.17)	
Royal Bolton	2/502	(0.4)	3/514	(0.6)	0.68 (0.07, 7.14)	
St. Mary's, Manchester	0/951	(0.0)	0/959	(0.0)	Not estimable	
Stoke Mandeville	2/1224	(0.2)	2/1230	(0.2)	1.00 (0.08, 13.17)	
UCLH	1/198	(0.5)	2/194	(1.0)	0.49 (0.02, 11.35)	
Warrington	4/1746	(0.2)	9/1750	(0.5)	0.45 (0.09, 2.20)	
Warwick	0/428	(0.0)	1/433	(0.2)	Not estimable	
**Total** (unadjusted)	43/23255	(0.2)	65/23341	(0.3)	0.66 (0.40, 1.10)	

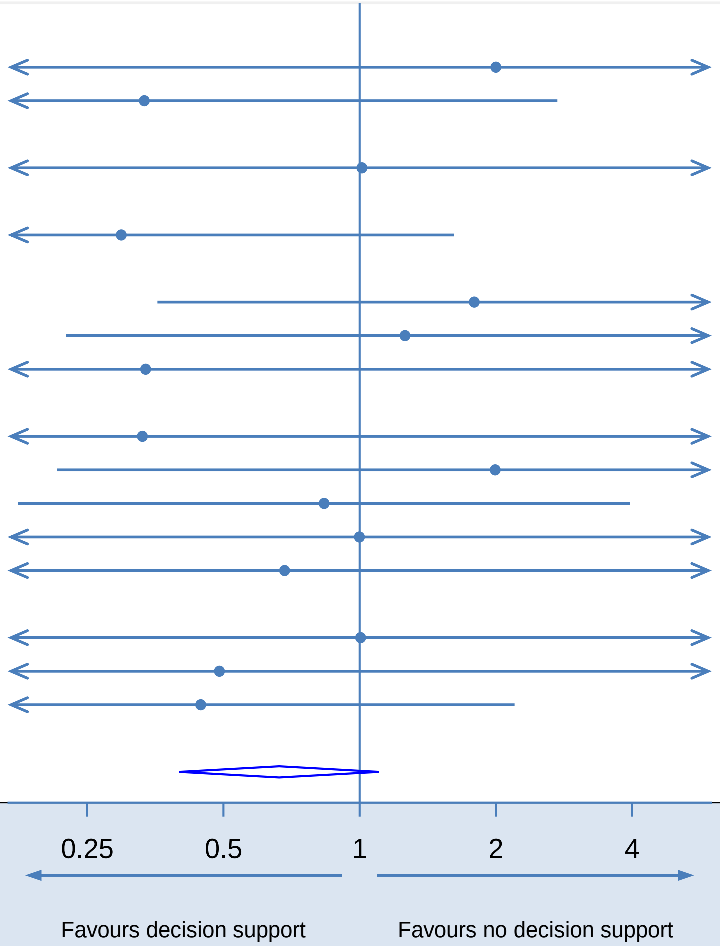

[a] P value calculated using centres with estimable risk ratios

Figure S9: Cord artery pH by centre

Subgroup analysis: Centre

	Decision support		No decision support		Risk ratio (99% CI)	P Value
	No of events/total no (%)					
**Cord artery pH <7.15**						
Birmingham Women's	53/701	(7.6)	67/705	(9.5)	0.80 (0.50, 1.25)	
Burnley General	108/1001	(10.8)	105/1017	(10.3)	1.05 (0.75, 1.46)	
Chelsea & Westminster, London	34/290	(11.7)	42/312	(13.5)	0.87 (0.50, 1.52)	
Coventry	23/379	(6.1)	38/370	(10.3)	0.59 (0.31, 1.14)	
Derby	17/230	(7.4)	22/233	(9.4)	0.78 (0.35, 1.74)	
Derriford, Plymouth	143/1476	(9.7)	147/1479	(9.9)	0.97 (0.73, 1.30)	
Homerton, London	101/853	(11.8)	92/832	(11.1)	1.07 (0.75, 1.52)	
Liverpool Women's	137/1322	(10.4)	148/1339	(11.1)	0.94 (0.70, 1.25)	
North Staffordshire	83/1174	(7.1)	71/1148	(6.2)	1.14 (0.76, 1.71)	
Northwick Park, London	46/544	(8.5)	51/540	(9.4)	0.90 (0.54, 1.48)	
Nottingham City & Queen's Medical Centre	46/498	(9.2)	48/491	(9.8)	0.94 (0.57, 1.57)	0.78
Princess Anne, Southampton	30/393	(7.6)	30/403	(7.4)	1.03 (0.54, 1.96)	
Princess Royal & Southern General, Glasgow	187/782	(23.9)	176/719	(24.5)	0.98 (0.77, 1.24)	
Queen Alexandra, Portsmouth	78/784	(9.9)	75/763	(9.8)	1.01 (0.68, 1.50)	
Rotunda, Dublin	69/830	(8.3)	79/801	(9.9)	0.84 (0.56, 1.26)	
Royal Bolton	60/299	(20.1)	57/305	(18.7)	1.07 (0.70, 1.65)	
St. Mary's, Manchester	72/613	(11.7)	77/625	(12.3)	0.95 (0.64, 1.42)	
Stoke Mandeville	145/723	(20.1)	159/752	(21.1)	0.95 (0.73, 1.23)	
UCLH	11/112	(9.8)	18/131	(13.7)	0.71 (0.27, 1.87)	
Warrington	111/1198	(9.3)	136/1175	(11.6)	0.80 (0.59, 1.09)	
Warwick	71/232	(30.6)	57/230	(24.8)	1.23 (0.83, 1.83)	
**Total** (unadjusted)	1625/14434	(11.3)	1695/14370	(11.8)	0.95 (0.88, 1.04)	

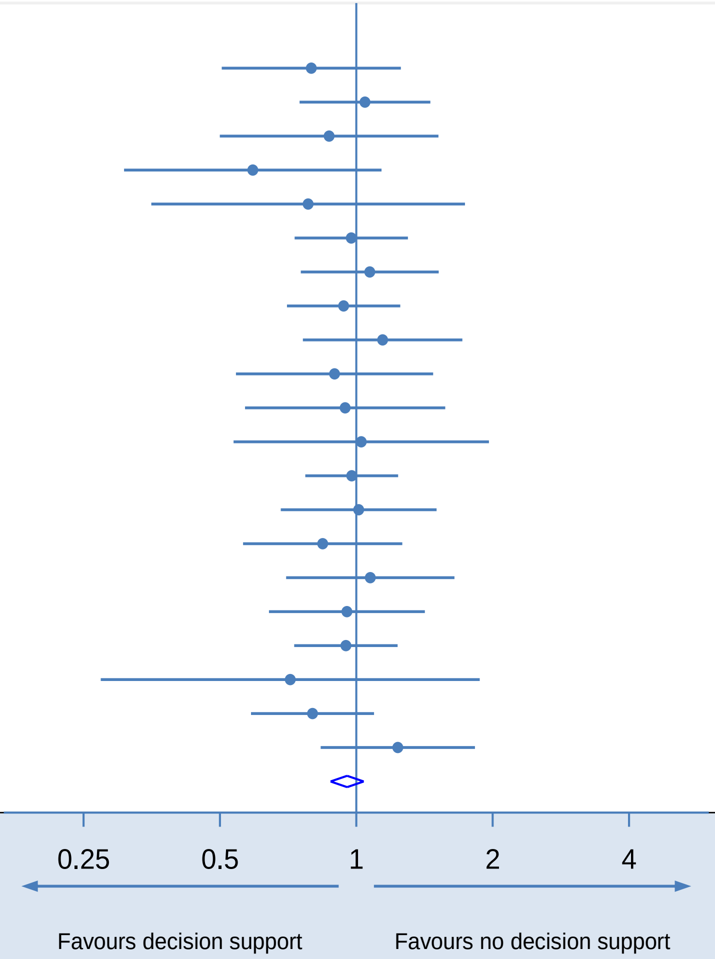

Figure S10: Metabolic acidosis by centre

Subgroup analysis: Centre

	Decision support<br>No of events/total no (%)		No decision support		Risk ratio (99% CI)	P Value[a]
**Metabolic acidosis**						
Birmingham Women's	2/319	(0.6)	2/328	(0.6)	1.03 (0.08, 13.38)	
Burnley General	13/984	(1.3)	8/1003	(0.8)	1.66 (0.52, 5.24)	
Chelsea & Westminster, London	2/287	(0.7)	3/307	(1.0)	0.71 (0.07, 7.43)	
Coventry	0/370	(0.0)	0/361	(0.0)	Not estimable	
Derby	3/226	(1.3)	2/232	(0.9)	1.54 (0.15, 16.02)	
Derriford, Plymouth	6/1426	(0.4)	2/1435	(0.1)	3.02 (0.37, 24.66)	
Homerton, London	13/853	(1.5)	10/832	(1.2)	1.27 (0.43, 3.72)	
Liverpool Women's	16/1296	(1.2)	9/1322	(0.7)	1.81 (0.62, 5.27)	
North Staffordshire	1/1140	(0.1)	1/1105	(0.1)	0.97 (0.03, 37.00)	
Northwick Park, London	6/518	(1.2)	12/513	(2.3)	0.50 (0.14, 1.78)	
Nottingham City & Queen's Medical Centre	4/489	(0.8)	1/484	(0.2)	3.96 (0.22, 69.97)	0.94
Princess Anne, Southampton	5/391	(1.3)	3/401	(0.7)	1.71 (0.26, 11.10)	
Princess Royal & Southern General, Glasgow	28/719	(3.9)	26/670	(3.9)	1.00 (0.50, 1.99)	
Queen Alexandra, Portsmouth	6/732	(0.8)	4/721	(0.6)	1.48 (0.28, 7.75)	
Rotunda, Dublin	2/827	(0.2)	4/795	(0.5)	0.48 (0.05, 4.46)	
Royal Bolton	6/299	(2.0)	6/304	(2.0)	1.02 (0.23, 4.44)	
St. Mary's, Manchester	2/591	(0.3)	2/607	(0.3)	1.03 (0.08, 13.46)	
Stoke Mandeville	9/711	(1.3)	11/744	(1.5)	0.86 (0.27, 2.70)	
UCLH	1/110	(0.9)	1/129	(0.8)	1.17 (0.03, 44.47)	
Warrington	10/1170	(0.9)	11/1144	(1.0)	0.89 (0.29, 2.73)	
Warwick	13/228	(5.7)	13/227	(5.7)	1.00 (0.37, 2.66)	
**Total** (unadjusted)	148/13686	(1.1)	131/13664	(1.0)	1.13 (0.83, 1.53)	

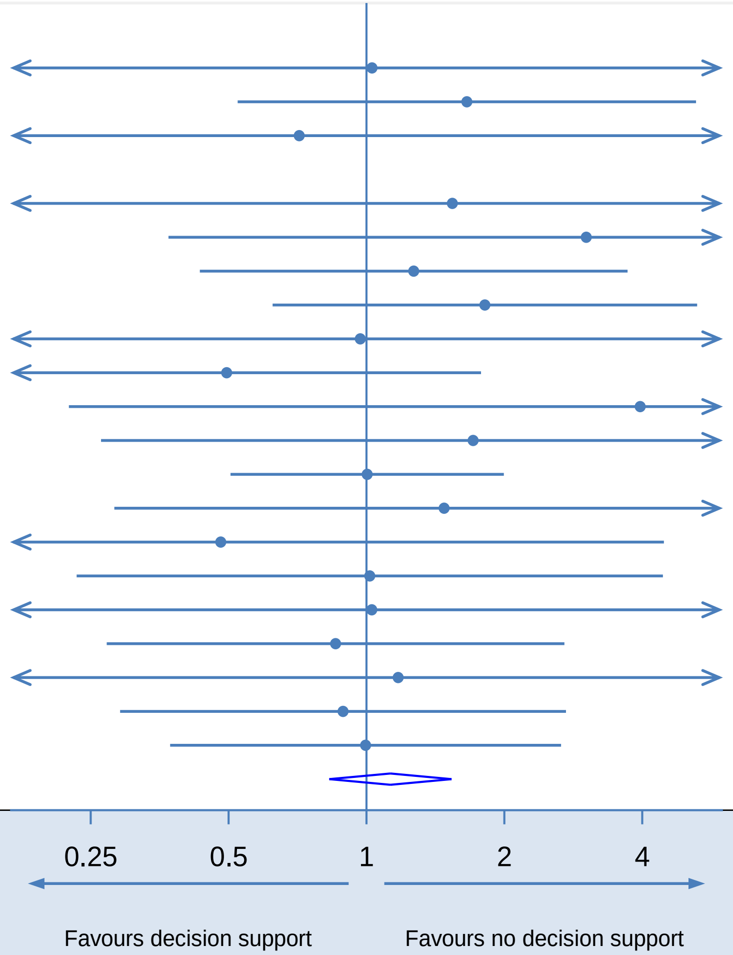

[a] P value calculated using centres with estimable risk ratios

Figure S11: Resuscitation by centre

Subgroup analysis: Centre

	Decision support		No decision support		Risk ratio (99% CI)	P Value[a]
	No of events/total no (%)					
**Resuscitation**						
Birmingham Women's[b]	0/0		0/0		Not estimable	
Burnley General	303/2091	(14.5)	321/2097	(15.3)	0.95 (0.78, 1.14)	
Chelsea & Westminster, London	30/443	(6.8)	30/449	(6.7)	1.01 (0.53, 1.93)	
Coventry	47/602	(7.8)	45/609	(7.4)	1.06 (0.63, 1.77)	
Derby	42/427	(9.8)	45/433	(10.4)	0.95 (0.56, 1.60)	
Derriford, Plymouth	292/1665	(17.5)	279/1650	(16.9)	1.04 (0.85, 1.26)	
Homerton, London[b]	0/0		0/0		Not estimable	
Liverpool Women's	224/1549	(14.5)	208/1586	(13.1)	1.10 (0.88, 1.39)	
North Staffordshire	241/1682	(14.3)	229/1695	(13.5)	1.06 (0.85, 1.32)	
Northwick Park, London	70/935	(7.5)	63/943	(6.7)	1.12 (0.73, 1.72)	
Nottingham City & Queen's Medical Centre	107/917	(11.7)	128/920	(13.9)	0.84 (0.61, 1.15)	0.96
Princess Anne, Southampton	118/590	(20.0)	98/586	(16.7)	1.20 (0.87, 1.64)	
Princess Royal & Southern General, Glasgow	214/2054	(10.4)	205/2047	(10.0)	1.04 (0.82, 1.32)	
Queen Alexandra, Portsmouth	163/1393	(11.7)	159/1395	(11.4)	1.03 (0.78, 1.34)	
Rotunda, Dublin	282/1754	(16.1)	262/1752	(15.0)	1.08 (0.87, 1.32)	
Royal Bolton	71/502	(14.1)	81/514	(15.8)	0.90 (0.61, 1.32)	
St. Mary's, Manchester	99/951	(10.4)	109/962	(11.3)	0.92 (0.66, 1.29)	
Stoke Mandeville	134/1224	(10.9)	131/1230	(10.7)	1.03 (0.76, 1.39)	
UCLH	11/197	(5.6)	14/194	(7.2)	0.77 (0.27, 2.18)	
Warrington	215/1746	(12.3)	204/1750	(11.7)	1.06 (0.83, 1.34)	
Warwick	30/428	(7.0)	29/433	(6.7)	1.05 (0.55, 2.00)	
**Total** (unadjusted)	2693/21150	(12.7)	2640/21245	(12.4)	1.02 (0.96, 1.09)	

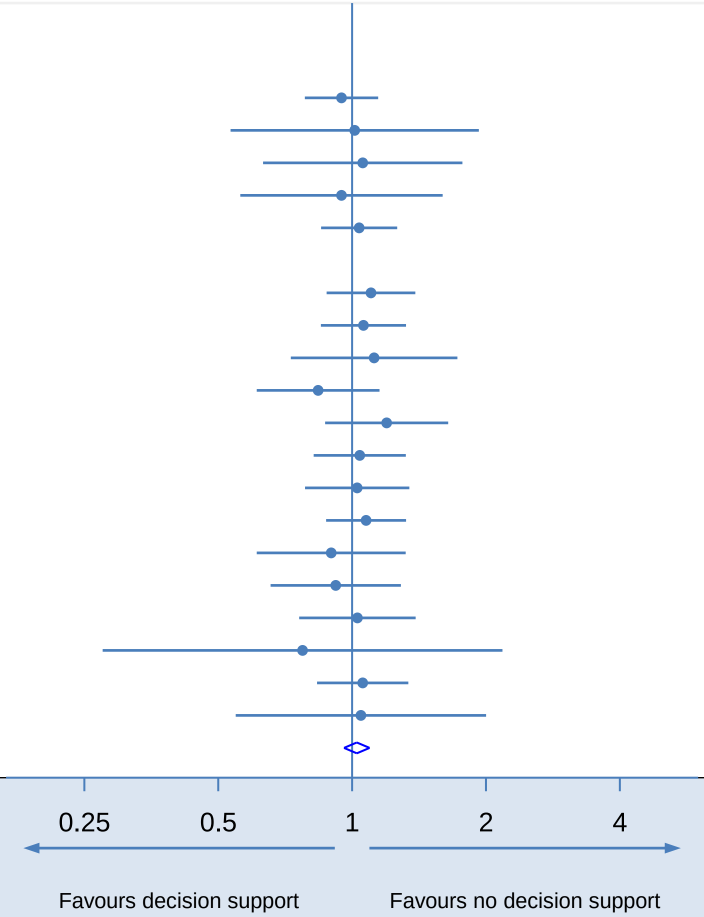

[a] P value calculated using centres with estimable risk ratios
[b] Resuscitation data not recorded in the Guardian system at Birmingham and Homerton

Figure S12: Seizures by centre

Subgroup analysis: Centre

	Decision support *No of events/total no (%)*		No decision support		Risk ratio (99% CI)	P Value[a]
**Seizures whilst at hospital**						
Birmingham Women's	1/1138	(0.1)	4/1136	(0.4)	0.25 (0.01, 4.44)	
Burnley General	1/2086	(0.0)	2/2090	(0.1)	0.50 (0.02, 11.74)	
Chelsea & Westminster, London	1/442	(0.2)	0/448	(0.0)	Not estimable	
Coventry	2/605	(0.3)	1/611	(0.2)	2.02 (0.09, 47.26)	
Derby	0/426	(0.0)	1/430	(0.2)	Not estimable	
Derriford, Plymouth	1/1656	(0.1)	6/1645	(0.4)	0.17 (0.01, 2.67)	
Homerton, London	3/956	(0.3)	2/949	(0.2)	1.49 (0.14, 15.60)	
Liverpool Women's	5/1542	(0.3)	3/1578	(0.2)	1.71 (0.26, 11.16)	
North Staffordshire	6/1679	(0.4)	1/1690	(0.1)	6.04 (0.37, 97.47)	
Northwick Park, London	5/933	(0.5)	2/940	(0.2)	2.52 (0.29, 21.68)	
Nottingham City & Queen's Medical Centre	2/916	(0.2)	1/916	(0.1)	2.00 (0.09, 46.83)	0.43
Princess Anne, Southampton	0/590	(0.0)	3/586	(0.5)	Not estimable	
Princess Royal & Southern General, Glasgow	5/2044	(0.2)	3/2038	(0.1)	1.66 (0.25, 10.89)	
Queen Alexandra, Portsmouth	1/1390	(0.1)	1/1391	(0.1)	1.00 (0.03, 38.20)	
Rotunda, Dublin	2/1749	(0.1)	5/1748	(0.3)	0.40 (0.05, 3.44)	
Royal Bolton	0/502	(0.0)	0/511	(0.0)	Not estimable	
St. Mary's, Manchester	2/947	(0.2)	0/957	(0.0)	Not estimable	
Stoke Mandeville	0/1223	(0.0)	1/1227	(0.1)	Not estimable	
UCLH	1/195	(0.5)	1/192	(0.5)	0.98 (0.03, 37.44)	
Warrington	1/1736	(0.1)	4/1743	(0.2)	0.25 (0.01, 4.47)	
Warwick	0/424	(0.0)	0/432	(0.0)	Not estimable	
**Total** (unadjusted)	39/23179	(0.2)	41/23258	(0.2)	0.95 (0.54, 1.70)	

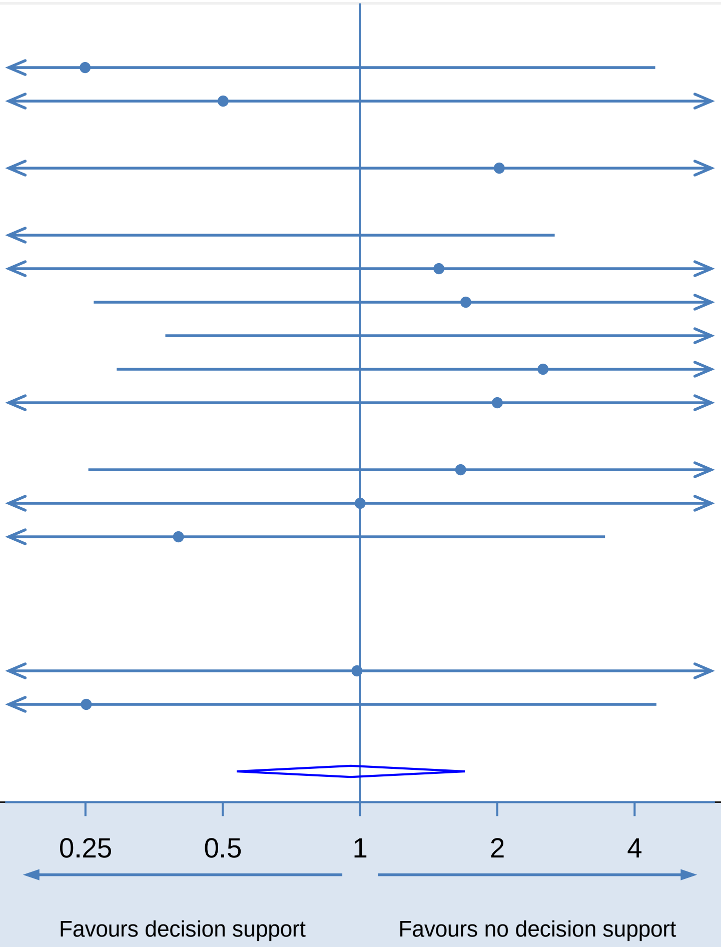

[a] P value calculated using centres with estimable risk ratios

Figure S13: Baby destination immediately after birth by centre

Subgroup analysis: Centre

	Decision support		No decision support		Risk ratio (99% CI)	P Value
	No of events/total no (%)					
**Baby destination ward or home**						
Birmingham Women's	908/986	(92.1)	920/988	(93.1)	0.99 (0.96, 1.02)	
Burnley General	2019/2089	(96.6)	2028/2092	(96.9)	1.00 (0.98, 1.01)	
Chelsea & Westminster, London	426/442	(96.4)	440/450	(97.8)	0.99 (0.96, 1.02)	
Coventry	583/599	(97.3)	588/605	(97.2)	1.00 (0.98, 1.03)	
Derby	417/427	(97.7)	425/433	(98.2)	0.99 (0.97, 1.02)	
Derriford, Plymouth	1382/1664	(83.1)	1398/1650	(84.7)	0.98 (0.94, 1.02)	
Homerton, London	913/951	(96.0)	912/948	(96.2)	1.00 (0.97, 1.02)	
Liverpool Women's	1505/1548	(97.2)	1525/1581	(96.5)	1.01 (0.99, 1.02)	
North Staffordshire	1648/1682	(98.0)	1662/1695	(98.1)	1.00 (0.99, 1.01)	
Northwick Park, London	886/929	(95.4)	882/939	(93.9)	1.02 (0.99, 1.04)	
Nottingham City & Queen's Medical Centre	891/916	(97.3)	895/916	(97.7)	1.00 (0.98, 1.01)	0.12
Princess Anne, Southampton	564/588	(95.9)	550/586	(93.9)	1.02 (0.99, 1.06)	
Princess Royal & Southern General, Glasgow	2007/2048	(98.0)	1990/2040	(97.5)	1.00 (0.99, 1.02)	
Queen Alexandra, Portsmouth	1351/1392	(97.1)	1369/1395	(98.1)	0.99 (0.97, 1.00)	
Rotunda, Dublin	1700/1746	(97.4)	1699/1747	(97.3)	1.00 (0.99, 1.02)	
Royal Bolton	434/502	(86.5)	453/513	(88.3)	0.98 (0.92, 1.04)	
St. Mary's, Manchester	916/943	(97.1)	932/956	(97.5)	1.00 (0.98, 1.02)	
Stoke Mandeville	1187/1221	(97.2)	1191/1227	(97.1)	1.00 (0.98, 1.02)	
UCLH	189/197	(95.9)	192/194	(99.0)	0.97 (0.93, 1.01)	
Warrington	1707/1745	(97.8)	1682/1748	(96.2)	1.02 (1.00, 1.03)	
Warwick	405/427	(94.8)	416/433	(96.1)	0.99 (0.95, 1.03)	
**Total** (unadjusted)	22038/23042	(95.6)	22149/23136	(95.7)	1.00 (0.99, 1.00)	

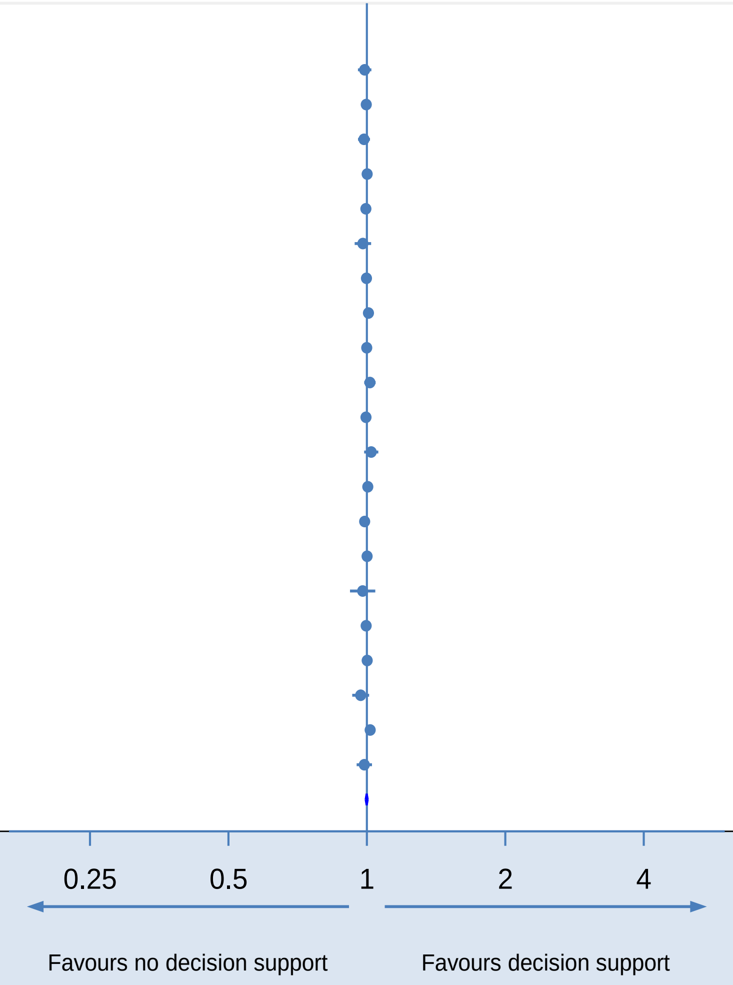

Figure S14: Length of hospital stay to discharge by centre

Subgroup analysis: Centre

	Decision support	No decision support	Hazard ratio (99% CI)	P Value
	Median time to event (days)			
**Length of hospital stay to discharge**				
Birmingham Women's	2	2	0.94 (0.87, 1.03)	
Burnley General	2	2	1.03 (0.96, 1.10)	
Chelsea & Westminster, London	2	2	1.03 (0.90, 1.18)	
Coventry	2	2	0.98 (0.88, 1.10)	
Derby	2	2	1.10 (0.96, 1.27)	
Derriford, Plymouth	1	1	0.99 (0.92, 1.07)	
Homerton, London	2	2	0.98 (0.89, 1.07)	
Liverpool Women's	2	2	0.98 (0.91, 1.05)	
North Staffordshire	2	2	1.01 (0.94, 1.08)	
Northwick Park, London	2	2	1.03 (0.93, 1.13)	
Nottingham City & Queen's Medical Centre	2	2	0.99 (0.90, 1.09)	0.50
Princess Anne, Southampton	2	2	1.03 (0.91, 1.16)	
Princess Royal & Southern General, Glasgow	2	2	1.00 (0.94, 1.06)	
Queen Alexandra, Portsmouth	1	1	0.99 (0.92, 1.07)	
Rotunda, Dublin	2	2	0.97 (0.91, 1.04)	
Royal Bolton	2	2	0.91 (0.80, 1.05)	
St. Mary's, Manchester	2	2	0.94 (0.85, 1.04)	
Stoke Mandeville	2	2	0.98 (0.91, 1.06)	
UCLH	1	1	1.04 (0.86, 1.27)	
Warrington	1	2	1.02 (0.96, 1.09)	
Warwick	2	2	0.92 (0.80, 1.05)	
**Total** (unadjusted)	2	2	0.99 (0.97, 1.01)	

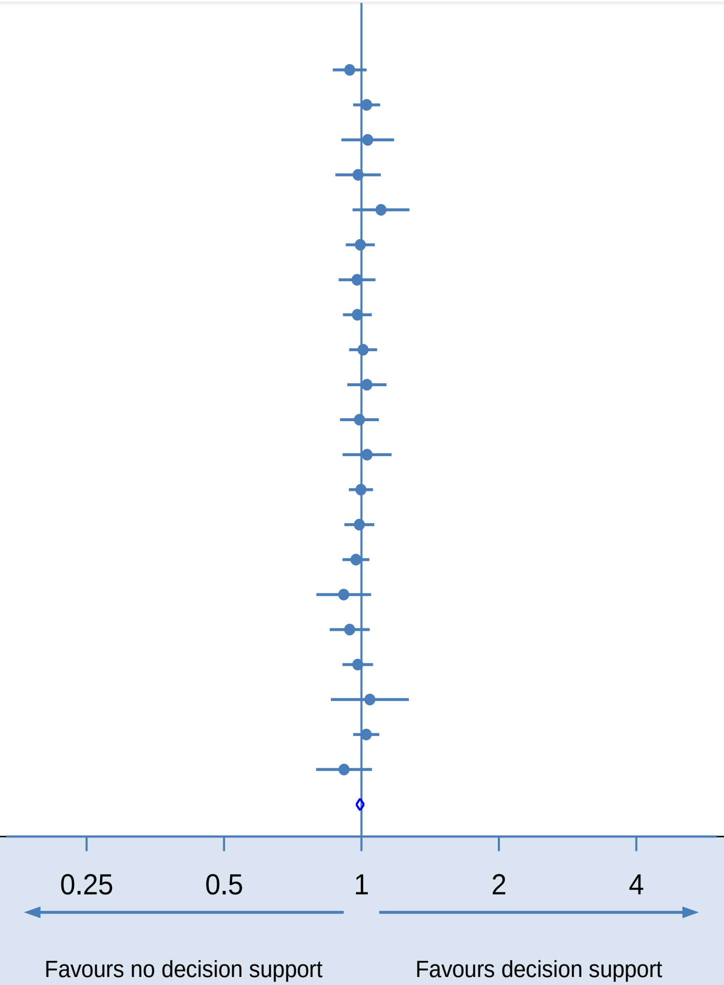

Figure S15: Epidural analgesia after trial entry by centre

Subgroup analysis: Centre

	Decision support No of events/total no	(%)	No decision support No of events/total no	(%)	Risk ratio (99% CI)	P Value[a]
**Epidural analgesia after trial entry[b]**						
Birmingham Women's	126/386	(32.6)	124/384	(32.3)	1.01 (0.77, 1.32)	
Burnley General	149/541	(27.5)	154/533	(28.9)	0.95 (0.74, 1.23)	
Chelsea & Westminster, London	151/418	(36.1)	169/417	(40.5)	0.89 (0.71, 1.12)	
Coventry	1/25	(4.0)	0/17	(0.0)	Not estimable	
Derby	130/417	(31.2)	124/412	(30.1)	1.04 (0.79, 1.36)	
Derriford, Plymouth	382/1048	(36.5)	399/1030	(38.7)	0.94 (0.81, 1.09)	
Homerton, London	82/242	(33.9)	107/250	(42.8)	0.79 (0.59, 1.07)	
Liverpool Women's	102/984	(10.4)	105/1000	(10.5)	0.99 (0.70, 1.39)	
North Staffordshire	73/286	(25.5)	61/289	(21.1)	1.21 (0.82, 1.79)	
Northwick Park, London	28/128	(21.9)	30/130	(23.1)	0.95 (0.52, 1.72)	
Nottingham City & Queen's Medical Centre	2/3	(66.7)	2/4	(50.0)	1.33 (0.22, 8.03)	0.37
Princess Anne, Southampton	5/46	(10.9)	6/46	(13.0)	0.83 (0.19, 3.63)	
Princess Royal & Southern General, Glasgow	109/253	(43.1)	97/256	(37.9)	1.14 (0.86, 1.50)	
Queen Alexandra, Portsmouth	254/1010	(25.1)	233/1011	(23.0)	1.09 (0.89, 1.34)	
Rotunda, Dublin	380/1674	(22.7)	361/1665	(21.7)	1.05 (0.89, 1.24)	
Royal Bolton	142/491	(28.9)	124/501	(24.8)	1.17 (0.89, 1.53)	
St. Mary's, Manchester	96/226	(42.5)	90/236	(38.1)	1.11 (0.83, 1.49)	
Stoke Mandeville	228/779	(29.3)	226/777	(29.1)	1.01 (0.82, 1.23)	
UCLH	72/182	(39.6)	58/179	(32.4)	1.22 (0.85, 1.76)	
Warrington	172/689	(25.0)	151/679	(22.2)	1.12 (0.87, 1.44)	
Warwick	86/325	(26.5)	68/326	(20.9)	1.27 (0.88, 1.83)	
**Total** (unadjusted)	2770/10153	(27.3)	2689/10142	(26.5)	1.03 (0.97, 1.09)	

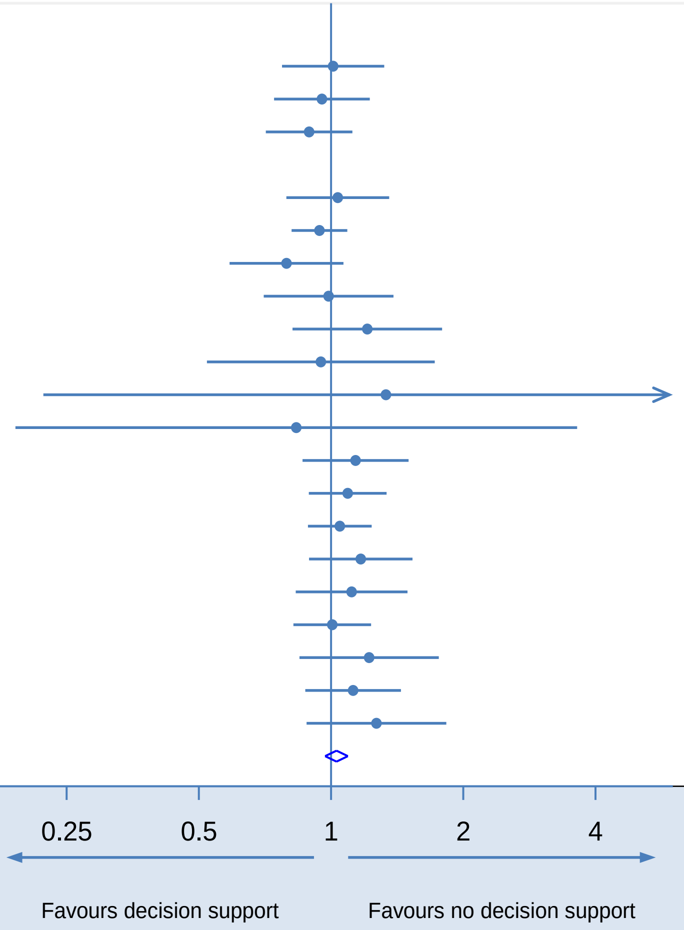

[a] P value calculated using centres with estimable risk ratios
[b] Timing of epidural in relation to trial entry only collected from 2013 onwards for each centre

Figure S16: Labour augmentation after trial entry by centre

Subgroup analysis: Centre

	Decision support		No decision support		Risk ratio (99% CI)	P Value[a]
	No of events/total no (%)					
**Labour augmentation after trial entry[b]**						
Birmingham Women's	197/385	(51.2)	194/383	(50.7)	1.01 (0.84, 1.21)	
Burnley General	196/534	(36.7)	230/527	(43.6)	0.84 (0.69, 1.02)	
Chelsea & Westminster, London	119/375	(31.7)	123/383	(32.1)	0.99 (0.75, 1.30)	
Coventry	7/29	(24.1)	6/27	(22.2)	1.09 (0.31, 3.86)	
Derby	175/413	(42.4)	176/410	(42.9)	0.99 (0.80, 1.22)	
Derriford, Plymouth	449/1036	(43.3)	439/1019	(43.1)	1.01 (0.88, 1.15)	
Homerton, London	103/242	(42.6)	99/245	(40.4)	1.05 (0.80, 1.39)	
Liverpool Women's	95/702	(13.5)	107/743	(14.4)	0.94 (0.67, 1.32)	
North Staffordshire	68/271	(25.1)	79/274	(28.8)	0.87 (0.60, 1.25)	
Northwick Park, London	24/152	(15.8)	30/156	(19.2)	0.82 (0.43, 1.56)	
Nottingham City & Queen's Medical Centre	1/5	(20.0)	0/5	(0.0)	Not estimable	0.92
Princess Anne, Southampton	12/48	(25.0)	14/47	(29.8)	0.84 (0.35, 2.00)	
Princess Royal & Southern General, Glasgow	123/253	(48.6)	114/254	(44.9)	1.08 (0.85, 1.38)	
Queen Alexandra, Portsmouth	378/1246	(30.3)	362/1250	(29.0)	1.05 (0.89, 1.23)	
Rotunda, Dublin	75/836	(9.0)	74/804	(9.2)	0.97 (0.65, 1.46)	
Royal Bolton	189/492	(38.4)	201/498	(40.4)	0.95 (0.78, 1.17)	
St. Mary's, Manchester	111/221	(50.2)	127/236	(53.8)	0.93 (0.74, 1.18)	
Stoke Mandeville	71/528	(13.4)	73/526	(13.9)	0.97 (0.65, 1.44)	
UCLH	88/184	(47.8)	75/180	(41.7)	1.15 (0.85, 1.55)	
Warrington	124/479	(25.9)	128/502	(25.5)	1.02 (0.77, 1.34)	
Warwick	100/321	(31.2)	99/323	(30.7)	1.02 (0.75, 1.38)	
**Total** (unadjusted)	2705/8752	(30.9)	2750/8792	(31.3)	0.99 (0.93, 1.05)	

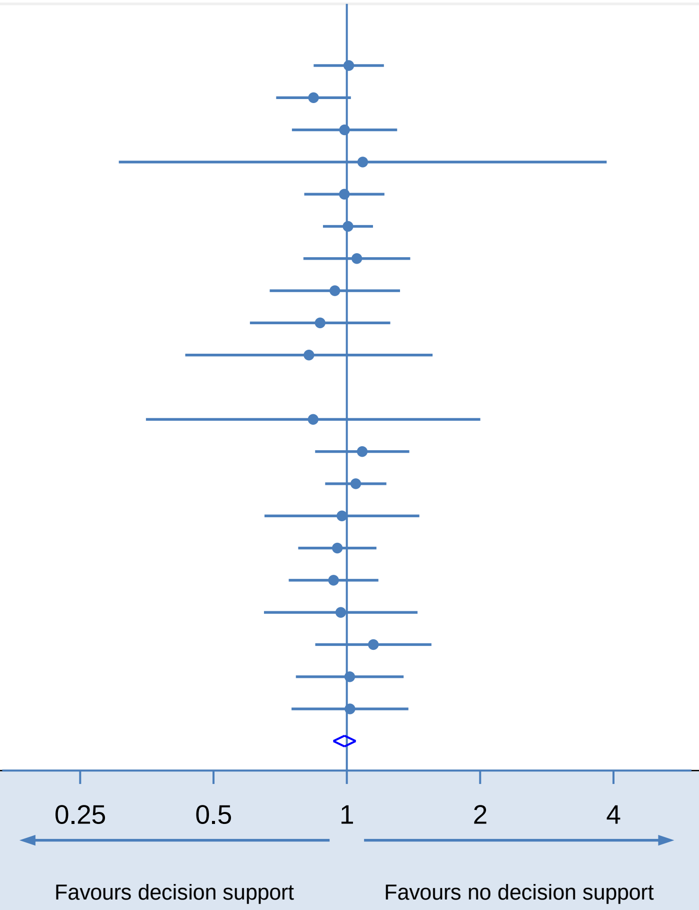

[a] P value calculated using centres with estimable risk ratios
[b] Timing of labour augmentation in relation to trial entry only collected from 2013 onwards for each centre

Figure S17: Presence of meconium after trial entry by centre

Subgroup analysis: Centre

	Decision support		No decision support		Risk ratio (99% CI)	P Value[a]
	No of events/total no (%)					
**Presence of meconium after trial entry[b]**						
Birmingham Women's	26/392	(6.6)	14/387	(3.6)	1.83 (0.80, 4.22)	
Burnley General	33/531	(6.2)	46/533	(8.6)	0.72 (0.41, 1.27)	
Chelsea & Westminster, London	8/363	(2.2)	9/367	(2.5)	0.90 (0.26, 3.10)	
Coventry	0/38	(0.0)	0/44	(0.0)	Not estimable	
Derby	20/408	(4.9)	11/411	(2.7)	1.83 (0.71, 4.74)	
Derriford, Plymouth	70/1004	(7.0)	69/998	(6.9)	1.01 (0.66, 1.54)	
Homerton, London	16/240	(6.7)	25/246	(10.2)	0.66 (0.30, 1.45)	
Liverpool Women's	15/1381	(1.1)	13/1403	(0.9)	1.17 (0.44, 3.10)	
North Staffordshire	6/317	(1.9)	4/320	(1.3)	1.51 (0.29, 7.90)	
Northwick Park, London	3/116	(2.6)	7/130	(5.4)	0.48 (0.08, 2.76)	
Nottingham City & Queen's Medical Centre	0/0		0/0		Not estimable	0.22
Princess Anne, Southampton	4/40	(10.0)	1/47	(2.1)	4.70 (0.27, 80.64)	
Princess Royal & Southern General, Glasgow	23/242	(9.5)	15/250	(6.0)	1.58 (0.70, 3.61)	
Queen Alexandra, Portsmouth	54/1151	(4.7)	60/1164	(5.2)	0.91 (0.57, 1.46)	
Rotunda, Dublin	21/1431	(1.5)	25/1409	(1.8)	0.83 (0.39, 1.76)	
Royal Bolton	30/480	(6.3)	42/482	(8.7)	0.72 (0.40, 1.30)	
St. Mary's, Manchester	19/229	(8.3)	16/231	(6.9)	1.20 (0.52, 2.78)	
Stoke Mandeville	17/204	(8.3)	14/200	(7.0)	1.19 (0.49, 2.91)	
UCLH	10/182	(5.5)	16/179	(8.9)	0.61 (0.23, 1.68)	
Warrington	44/691	(6.4)	58/689	(8.4)	0.76 (0.46, 1.24)	
Warwick	21/316	(6.6)	24/325	(7.4)	0.90 (0.43, 1.89)	
**Total** (unadjusted)	440/9756	(4.5)	469/9815	(4.8)	0.94 (0.80, 1.12)	

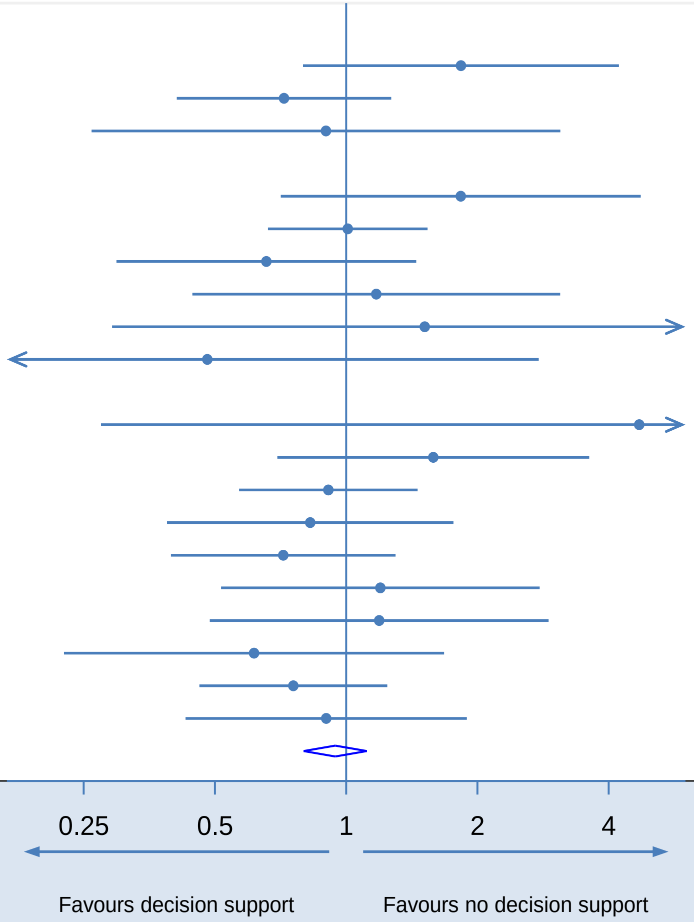

[a] P value calculated using centres with estimable risk ratios
[b] Timing of meconium in relation to trial entry only collected from 2013 onwards for each centre

Figure S18: Number of women with at least one blue, yellow or red level of concern by centre

Subgroup analysis: Centre

	Decision support<br>*No of events/total no (%)*		No decision support		Risk ratio (99% CI)	P Value
**Number of women with at least one blue, yellow or red level of concern**						
Birmingham Women's	1082/1114	(97.1)	1098/1117	(98.3)	0.99 (0.97, 1.00)	
Burnley General	1990/2020	(98.5)	1985/2020	(98.3)	1.00 (0.99, 1.01)	
Chelsea & Westminster, London	410/426	(96.2)	416/427	(97.4)	0.99 (0.96, 1.02)	
Coventry	519/569	(91.2)	531/584	(90.9)	1.00 (0.96, 1.05)	
Derby	400/417	(95.9)	401/415	(96.6)	0.99 (0.96, 1.03)	
Derriford, Plymouth	1627/1638	(99.3)	1599/1616	(98.9)	1.00 (1.00, 1.01)	
Homerton, London	887/905	(98.0)	879/898	(97.9)	1.00 (0.98, 1.02)	
Liverpool Women's	1500/1511	(99.3)	1541/1550	(99.4)	1.00 (0.99, 1.01)	
North Staffordshire	1485/1579	(94.0)	1478/1585	(93.2)	1.01 (0.98, 1.03)	
Northwick Park, London	848/890	(95.3)	860/889	(96.7)	0.98 (0.96, 1.01)	
Nottingham City & Queen's Medical Centre	873/895	(97.5)	885/901	(98.2)	0.99 (0.98, 1.01)	0.62
Princess Anne, Southampton	563/573	(98.3)	560/568	(98.6)	1.00 (0.98, 1.02)	
Princess Royal & Southern General, Glasgow	1992/2003	(99.5)	1986/1999	(99.3)	1.00 (0.99, 1.01)	
Queen Alexandra, Portsmouth	1321/1365	(96.8)	1316/1363	(96.6)	1.00 (0.98, 1.02)	
Rotunda, Dublin	1686/1726	(97.7)	1676/1719	(97.5)	1.00 (0.99, 1.02)	
Royal Bolton	483/496	(97.4)	496/504	(98.4)	0.99 (0.97, 1.01)	
St. Mary's, Manchester	899/924	(97.3)	925/938	(98.6)	0.99 (0.97, 1.00)	
Stoke Mandeville	1180/1208	(97.7)	1183/1212	(97.6)	1.00 (0.98, 1.02)	
UCLH	180/190	(94.7)	172/184	(93.5)	1.01 (0.95, 1.08)	
Warrington	1633/1663	(98.2)	1626/1657	(98.1)	1.00 (0.99, 1.01)	
Warwick	392/405	(96.8)	408/418	(97.6)	0.99 (0.96, 1.02)	
**Total** (unadjusted)	21950/22517	(97.5)	22021/22564	(97.6)	1.00 (1.00, 1.00)	

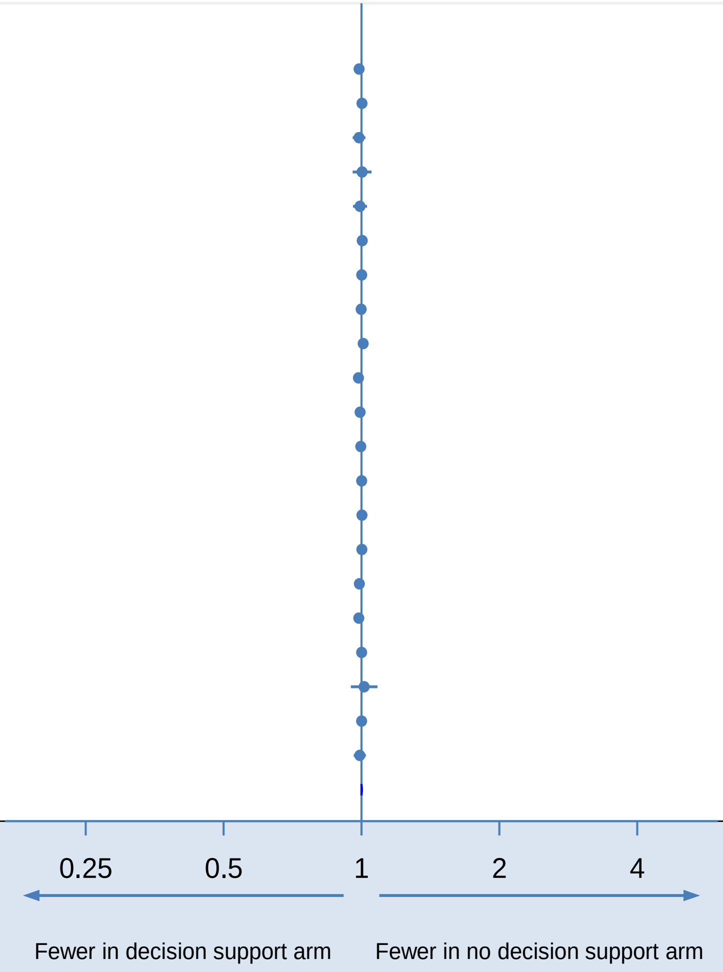

Figure S19: Number of women with at least one blue level of concern by centre

Subgroup analysis: Centre

	Decision support		No decision support		Risk ratio (99% CI)	P Value
	No of events/total no (%)					
**Number of women with at least one blue level of concern (mild abnormality)**						
Birmingham Women's	1079/1114	(96.9)	1096/1117	(98.1)	0.99 (0.97, 1.00)	
Burnley General	1987/2020	(98.4)	1982/2020	(98.1)	1.00 (0.99, 1.01)	
Chelsea & Westminster, London	410/426	(96.2)	414/427	(97.0)	0.99 (0.96, 1.03)	
Coventry	510/569	(89.6)	526/584	(90.1)	1.00 (0.95, 1.05)	
Derby	394/417	(94.5)	395/415	(95.2)	0.99 (0.95, 1.03)	
Derriford, Plymouth	1626/1638	(99.3)	1592/1616	(98.5)	1.01 (1.00, 1.02)	
Homerton, London	882/905	(97.5)	874/898	(97.3)	1.00 (0.98, 1.02)	
Liverpool Women's	1500/1511	(99.3)	1541/1550	(99.4)	1.00 (0.99, 1.01)	
North Staffordshire	1462/1579	(92.6)	1455/1585	(91.8)	1.01 (0.98, 1.04)	
Northwick Park, London	842/890	(94.6)	851/889	(95.7)	0.99 (0.96, 1.02)	
Nottingham City & Queen's Medical Centre	873/895	(97.5)	885/901	(98.2)	0.99 (0.98, 1.01)	0.39
Princess Anne, Southampton	563/573	(98.3)	559/568	(98.4)	1.00 (0.98, 1.02)	
Princess Royal & Southern General, Glasgow	1988/2003	(99.3)	1984/1999	(99.2)	1.00 (0.99, 1.01)	
Queen Alexandra, Portsmouth	1318/1365	(96.6)	1307/1363	(95.9)	1.01 (0.99, 1.03)	
Rotunda, Dublin	1676/1726	(97.1)	1658/1719	(96.5)	1.01 (0.99, 1.02)	
Royal Bolton	482/496	(97.2)	496/504	(98.4)	0.99 (0.96, 1.01)	
St. Mary's, Manchester	895/924	(96.9)	922/938	(98.3)	0.99 (0.97, 1.00)	
Stoke Mandeville	1178/1208	(97.5)	1180/1212	(97.4)	1.00 (0.98, 1.02)	
UCLH	180/190	(94.7)	170/184	(92.4)	1.03 (0.96, 1.10)	
Warrington	1629/1663	(98.0)	1621/1657	(97.8)	1.00 (0.99, 1.01)	
Warwick	389/405	(96.0)	405/418	(96.9)	0.99 (0.96, 1.03)	
**Total** (unadjusted)	21863/22517	(97.1)	21913/22564	(97.1)	1.00 (1.00, 1.00)	

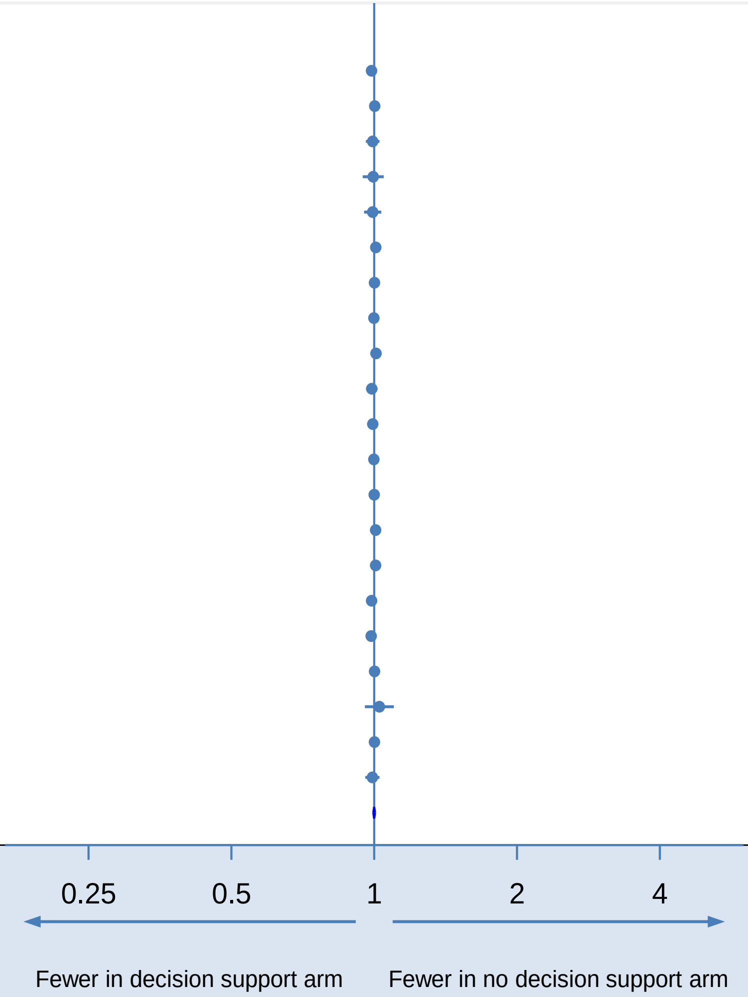

Figure S20: Number of women with at least one yellow level of concern by centre

Subgroup analysis: Centre

	Decision support		No decision support		Risk ratio (99% CI)	P Value
	No of events/total no (%)					
**Number of women with at least one yellow level of concern (moderate abnormality)**						
Birmingham Women's	880/1114	(79.0)	912/1117	(81.6)	0.97 (0.92, 1.02)	
Burnley General	1423/2020	(70.4)	1452/2020	(71.9)	0.98 (0.93, 1.03)	
Chelsea & Westminster, London	324/426	(76.1)	326/427	(76.3)	1.00 (0.90, 1.10)	
Coventry	390/569	(68.5)	401/584	(68.7)	1.00 (0.90, 1.11)	
Derby	303/417	(72.7)	302/415	(72.8)	1.00 (0.89, 1.11)	
Derriford, Plymouth	1228/1638	(75.0)	1221/1616	(75.6)	0.99 (0.94, 1.04)	
Homerton, London	752/905	(83.1)	761/898	(84.7)	0.98 (0.93, 1.03)	
Liverpool Women's	1149/1511	(76.0)	1157/1550	(74.6)	1.02 (0.97, 1.07)	
North Staffordshire	1018/1579	(64.5)	1035/1585	(65.3)	0.99 (0.92, 1.06)	
Northwick Park, London	649/890	(72.9)	636/889	(71.5)	1.02 (0.94, 1.10)	
Nottingham City & Queen's Medical Centre	661/895	(73.9)	655/901	(72.7)	1.02 (0.94, 1.09)	0.73
Princess Anne, Southampton	433/573	(75.6)	431/568	(75.9)	1.00 (0.91, 1.09)	
Princess Royal & Southern General, Glasgow	1418/2003	(70.8)	1363/1999	(68.2)	1.04 (0.98, 1.10)	
Queen Alexandra, Portsmouth	939/1365	(68.8)	895/1363	(65.7)	1.05 (0.98, 1.12)	
Rotunda, Dublin	1295/1726	(75.0)	1277/1719	(74.3)	1.01 (0.96, 1.06)	
Royal Bolton	411/496	(82.9)	405/504	(80.4)	1.03 (0.95, 1.11)	
St. Mary's, Manchester	706/924	(76.4)	735/938	(78.4)	0.98 (0.91, 1.04)	
Stoke Mandeville	894/1208	(74.0)	883/1212	(72.9)	1.02 (0.95, 1.08)	
UCLH	157/190	(82.6)	148/184	(80.4)	1.03 (0.90, 1.17)	
Warrington	1446/1663	(87.0)	1425/1657	(86.0)	1.01 (0.98, 1.04)	
Warwick	289/405	(71.4)	302/418	(72.2)	0.99 (0.88, 1.11)	
**Total** (unadjusted)	16765/22517	(74.5)	16722/22564	(74.1)	1.00 (0.99, 1.02)	

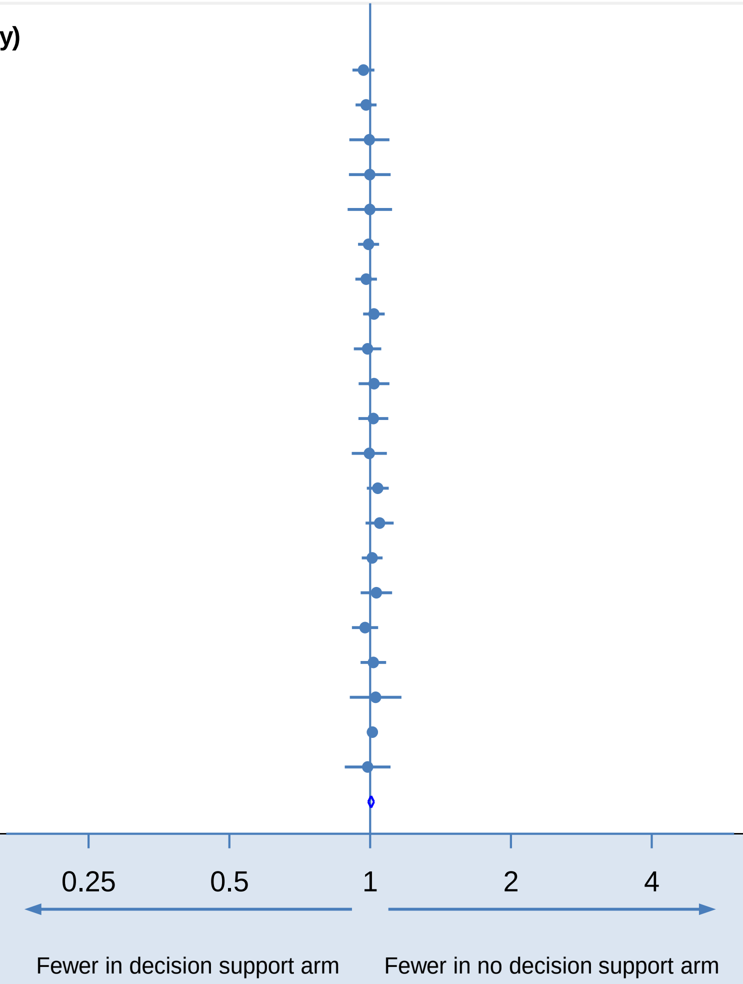

Figure S21: Number of women with at least one red level of concern by centre

Subgroup analysis: Centre

	Decision support		No decision support		Risk ratio (99% CI)	P Value
	No of events/total no (%)					
**Number of women with at least one red level of concern (severe abnormality)**						
Birmingham Women's	85/1114	(7.6)	109/1117	(9.8)	0.78 (0.55, 1.12)	
Burnley General	169/2020	(8.4)	219/2020	(10.8)	0.77 (0.60, 0.99)	
Chelsea & Westminster, London	54/426	(12.7)	67/427	(15.7)	0.81 (0.52, 1.25)	
Coventry	68/569	(12.0)	65/584	(11.1)	1.07 (0.71, 1.63)	
Derby[a]						
Derriford, Plymouth	200/1638	(12.2)	228/1616	(14.1)	0.87 (0.69, 1.09)	
Homerton, London	110/905	(12.2)	97/898	(10.8)	1.13 (0.80, 1.58)	
Liverpool Women's	128/1511	(8.5)	176/1550	(11.4)	0.75 (0.56, 0.99)	
North Staffordshire	124/1579	(7.9)	103/1585	(6.5)	1.21 (0.87, 1.68)	
Northwick Park, London	86/890	(9.7)	93/889	(10.5)	0.92 (0.64, 1.33)	
Nottingham City & Queen's Medical Centre	106/895	(11.8)	90/901	(10.0)	1.19 (0.84, 1.68)	0.01
Princess Anne, Southampton	75/573	(13.1)	77/568	(13.6)	0.97 (0.65, 1.42)	
Princess Royal & Southern General, Glasgow	227/2003	(11.3)	220/1999	(11.0)	1.03 (0.82, 1.30)	
Queen Alexandra, Portsmouth	79/1365	(5.8)	71/1363	(5.2)	1.11 (0.74, 1.67)	
Rotunda, Dublin	166/1726	(9.6)	177/1719	(10.3)	0.93 (0.72, 1.22)	
Royal Bolton	37/496	(7.5)	38/504	(7.5)	0.99 (0.56, 1.75)	
St. Mary's, Manchester	144/924	(15.6)	124/938	(13.2)	1.18 (0.88, 1.58)	
Stoke Mandeville	152/1208	(12.6)	119/1212	(9.8)	1.28 (0.95, 1.73)	
UCLH	31/190	(16.3)	24/184	(13.0)	1.25 (0.65, 2.39)	
Warrington	294/1663	(17.7)	316/1657	(19.1)	0.93 (0.77, 1.12)	
Warwick[a]						
**Total** (unadjusted)	2335/21695	(10.8)	2413/21731	(11.1)	0.97 (0.90, 1.04)	

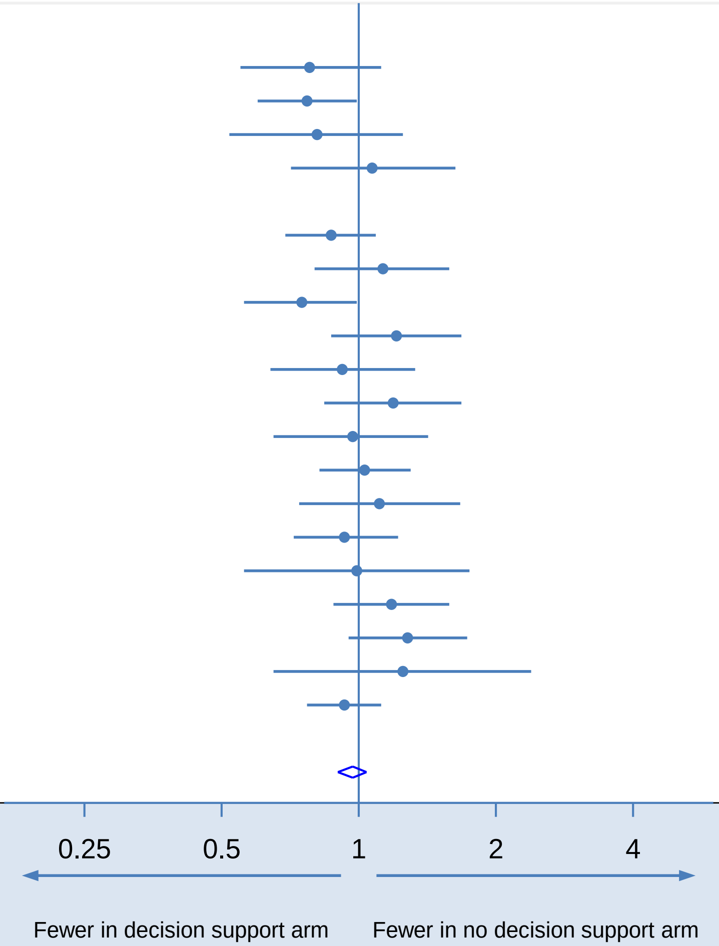

[a] Data on timing of red level of concerns not available for 2 centres: Warwick (n=823) and Derby (n=832)

Figure S22: Number of blue, yellow and red levels of concern by centre

Subgroup analysis: Centre

	Decision support	No decision support	Rate ratio (99% CI)	P Value
	Rate per hour			
**Number of blue, yellow and red levels of concern**				
Birmingham Women's	1.40	1.41	0.99 (0.96, 1.02)	
Burnley General	1.37	1.42	0.97 (0.94, 0.99)	
Chelsea & Westminster, London	1.20	1.28	0.93 (0.88, 0.99)	
Coventry	0.85	0.83	1.03 (0.97, 1.09)	
Derby	1.02	0.86	1.18 (1.11, 1.26)	
Derriford, Plymouth	1.47	1.50	0.98 (0.96, 1.01)	
Homerton, London	1.52	1.51	1.01 (0.98, 1.04)	
Liverpool Women's	1.52	1.62	0.94 (0.92, 0.96)	
North Staffordshire	1.11	1.15	0.96 (0.93, 0.99)	
Northwick Park, London	1.18	1.28	0.92 (0.88, 0.95)	
Nottingham City & Queen's Medical Centre	1.34	1.32	1.02 (0.98, 1.06)	<0.001
Princess Anne, Southampton	1.15	1.22	0.94 (0.90, 0.99)	
Princess Royal & Southern General, Glasgow	1.47	1.52	0.97 (0.94, 0.99)	
Queen Alexandra, Portsmouth	1.25	1.26	0.99 (0.96, 1.02)	
Rotunda, Dublin	1.52	1.60	0.95 (0.92, 0.98)	
Royal Bolton	1.50	1.18	1.27 (1.21, 1.34)	
St. Mary's, Manchester	1.40	1.45	0.97 (0.94, 1.00)	
Stoke Mandeville	1.35	1.33	1.02 (0.98, 1.05)	
UCLH	0.77	0.83	0.93 (0.86, 1.01)	
Warrington	1.47	1.55	0.95 (0.92, 0.97)	
Warwick	1.19	1.18	1.00 (0.94, 1.07)	
**Total** (unadjusted)	1.35	1.38	0.98 (0.97, 0.99)	

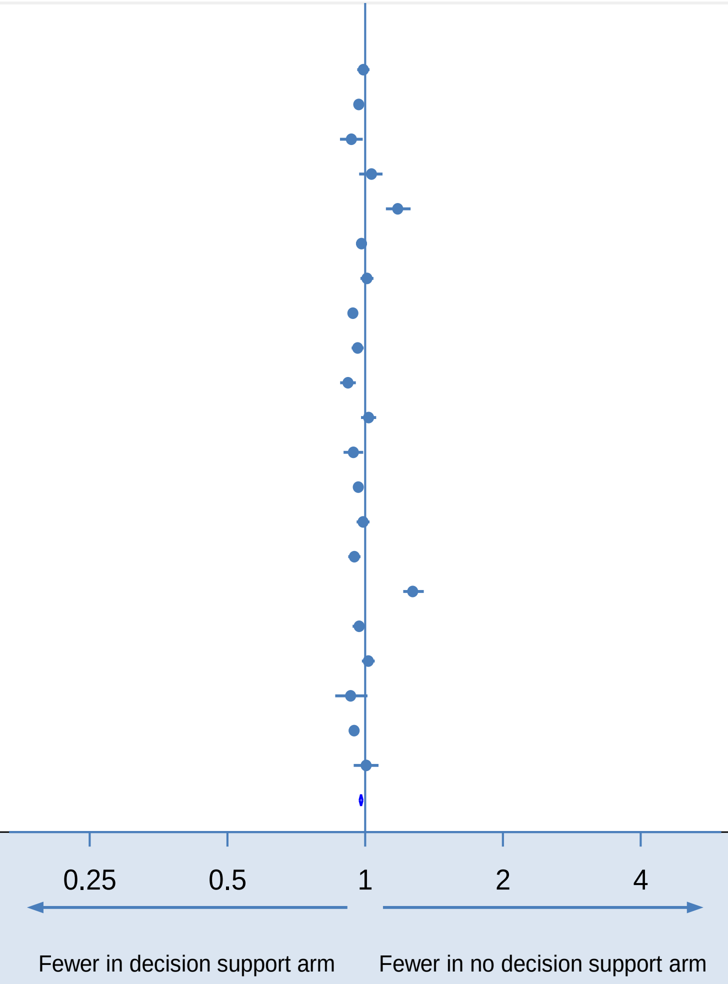

Figure S23: Number of blue levels of concern by centre

Subgroup analysis: Centre

	Decision support	No decision support	Rate ratio (99% CI)	P Value
	Rate per hour			
**Number of blue levels of concern**				
Birmingham Women's	1.02	0.98	1.04 (1.00, 1.08)	
Burnley General	1.10	1.10	1.00 (0.97, 1.03)	
Chelsea & Westminster, London	0.93	0.96	0.96 (0.90, 1.03)	
Coventry	0.63	0.60	1.05 (0.98, 1.12)	
Derby	0.73	0.59	1.23 (1.14, 1.33)	
Derriford, Plymouth	1.19	1.16	1.02 (0.99, 1.06)	
Homerton, London	1.07	1.03	1.05 (1.01, 1.09)	
Liverpool Women's	1.23	1.25	0.98 (0.96, 1.01)	
North Staffordshire	0.87	0.87	1.01 (0.98, 1.04)	
Northwick Park, London	0.86	0.89	0.97 (0.92, 1.01)	
Nottingham City & Queen's Medical Centre	1.09	1.06	1.03 (0.99, 1.08)	<0.001
Princess Anne, Southampton	0.87	0.89	0.98 (0.92, 1.03)	
Princess Royal & Southern General, Glasgow	1.21	1.23	0.99 (0.96, 1.02)	
Queen Alexandra, Portsmouth	0.98	0.97	1.01 (0.97, 1.05)	
Rotunda, Dublin	1.13	1.14	0.99 (0.96, 1.03)	
Royal Bolton	0.99	0.78	1.26 (1.19, 1.35)	
St. Mary's, Manchester	1.13	1.15	0.99 (0.95, 1.03)	
Stoke Mandeville	1.07	1.03	1.04 (1.00, 1.08)	
UCLH	0.50	0.52	0.96 (0.86, 1.07)	
Warrington	1.09	1.10	0.99 (0.96, 1.02)	
Warwick	0.92	0.88	1.04 (0.97, 1.12)	
**Total** (unadjusted)	1.04	1.03	1.01 (1.00, 1.02)	

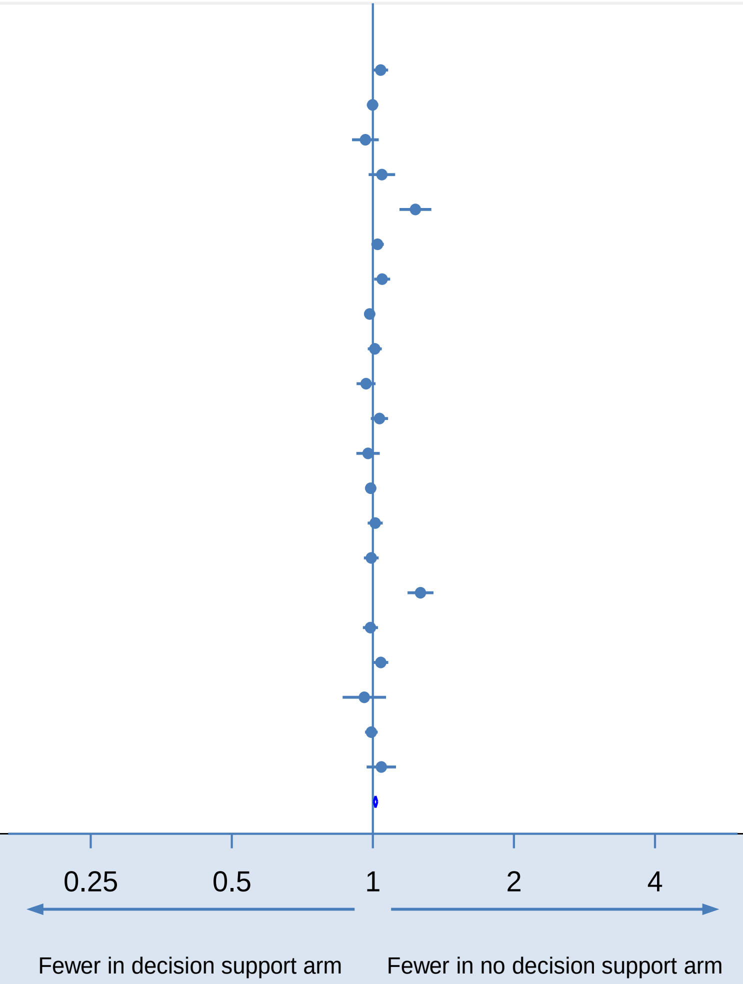

Figure S24: Number of yellow levels of concern by centre

Subgroup analysis: Centre

	Decision support	No decision support	Rate ratio (99% CI)	P Value
	Rate per hour			
**Number of yellow levels of concern**				
Birmingham Women's	0.37	0.42	0.89 (0.84, 0.94)	
Burnley General	0.26	0.30	0.87 (0.82, 0.92)	
Chelsea & Westminster, London	0.25	0.30	0.85 (0.75, 0.96)	
Coventry	0.21	0.21	0.98 (0.88, 1.10)	
Derby	0.30	0.28	1.08 (0.96, 1.22)	
Derriford, Plymouth	0.26	0.32	0.84 (0.79, 0.89)	
Homerton, London	0.43	0.47	0.92 (0.87, 0.98)	
Liverpool Women's	0.28	0.35	0.79 (0.75, 0.84)	
North Staffordshire	0.22	0.27	0.80 (0.75, 0.86)	
Northwick Park, London	0.30	0.37	0.80 (0.74, 0.86)	
Nottingham City & Queen's Medical Centre	0.22	0.24	0.93 (0.85, 1.02)	<0.001
Princess Anne, Southampton	0.26	0.30	0.85 (0.76, 0.94)	
Princess Royal & Southern General, Glasgow	0.24	0.28	0.86 (0.81, 0.91)	
Queen Alexandra, Portsmouth	0.26	0.28	0.91 (0.85, 0.98)	
Rotunda, Dublin	0.37	0.44	0.83 (0.78, 0.88)	
Royal Bolton	0.50	0.38	1.31 (1.20, 1.44)	
St. Mary's, Manchester	0.25	0.28	0.89 (0.83, 0.97)	
Stoke Mandeville	0.26	0.29	0.91 (0.84, 0.97)	
UCLH	0.26	0.30	0.88 (0.76, 1.02)	
Warrington	0.35	0.42	0.82 (0.78, 0.87)	
Warwick	0.26	0.30	0.89 (0.78, 1.01)	
**Total** (unadjusted)	0.29	0.33	0.87 (0.86, 0.89)	

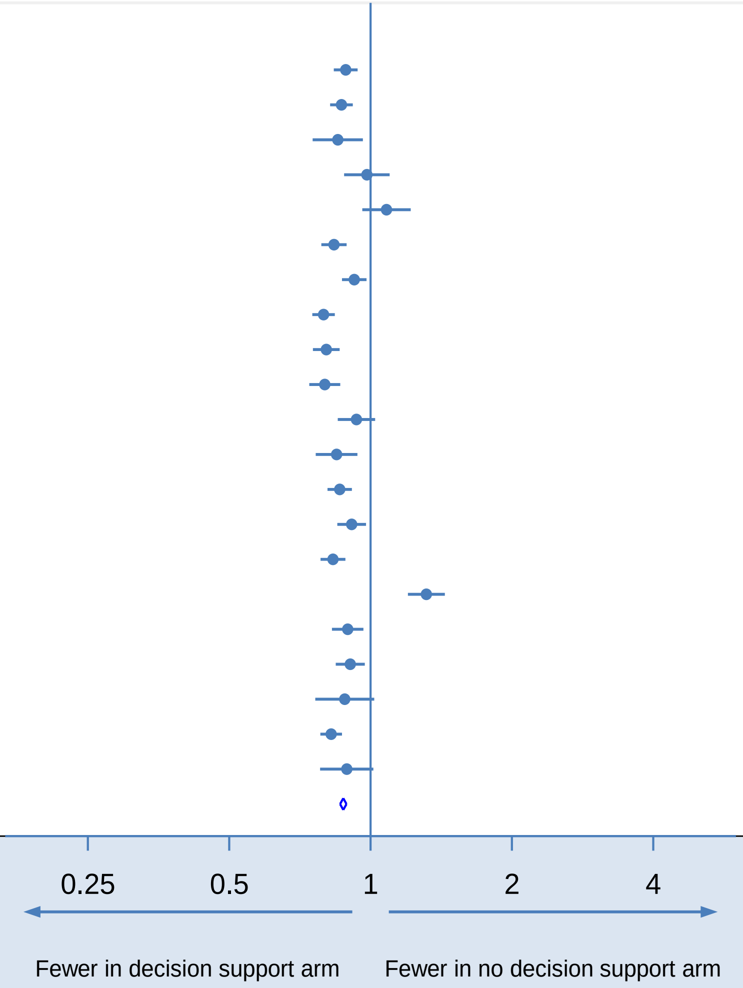

Figure S25: Number of red levels of concern by centre

Subgroup analysis: Centre

	Decision support	No decision support	Rate ratio (99% CI)	P Value
	Rate per hour			
**Number of red levels of concern**				
Birmingham Women's	0.01	0.01	0.73 (0.51, 1.03)	
Burnley General	0.01	0.02	0.78 (0.61, 0.99)	
Chelsea & Westminster, London	0.02	0.03	0.75 (0.48, 1.16)	
Coventry	0.02	0.02	1.12 (0.75, 1.70)	
Derby[a]				
Derriford, Plymouth	0.02	0.02	0.87 (0.69, 1.09)	
Homerton, London	0.02	0.02	1.13 (0.82, 1.55)	
Liverpool Women's	0.01	0.02	0.72 (0.55, 0.94)	
North Staffordshire	0.01	0.01	1.18 (0.87, 1.59)	
Northwick Park, London	0.01	0.02	0.85 (0.59, 1.21)	
Nottingham City & Queen's Medical Centre	0.02	0.02	1.28 (0.94, 1.76)	0.008
Princess Anne, Southampton	0.02	0.02	0.92 (0.64, 1.32)	
Princess Royal & Southern General, Glasgow	0.02	0.02	1.01 (0.81, 1.26)	
Queen Alexandra, Portsmouth	0.01	0.01	1.02 (0.68, 1.52)	
Rotunda, Dublin	0.02	0.02	0.92 (0.71, 1.21)	
Royal Bolton	0.01	0.01	0.77 (0.43, 1.36)	
St. Mary's, Manchester	0.02	0.02	0.97 (0.73, 1.29)	
Stoke Mandeville	0.02	0.02	1.54 (1.17, 2.04)	
UCLH	0.01	0.01	0.98 (0.50, 1.96)	
Warrington	0.03	0.03	0.93 (0.76, 1.12)	
Warwick[a]				
**Total** (unadjusted)	0.02	0.02	0.96 (0.89, 1.02)	

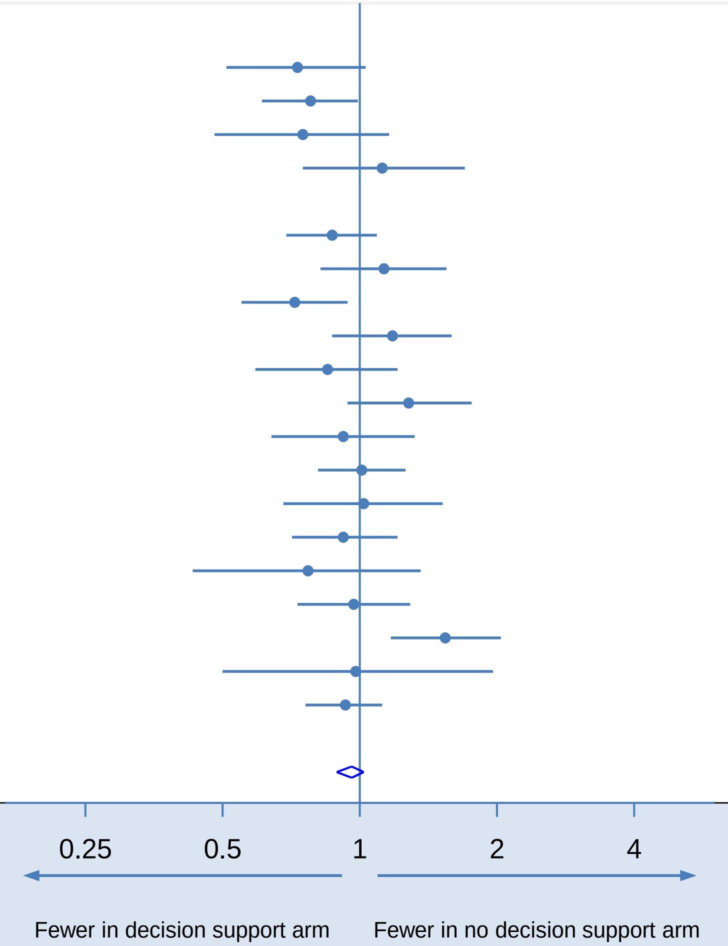

[a] Data on timing of red level of concerns not available for 2 centres: Warwick (n=823) and Derby (n=832)

Figure S26: Time from last red level of concern to delivery

Subgroup analysis: Centre

	Decision support	No decision support	Hazard ratio (99% CI)	P Value
	Median time to event (minutes)			
**Time from last red level of concern to delivery**[a]				
Birmingham Women's	47	51	0.98 (0.68, 1.42)	
Burnley General	42	35	0.94 (0.72, 1.22)	
Chelsea & Westminster, London	74	45	1.03 (0.65, 1.65)	
Coventry	89	93	1.01 (0.64, 1.58)	
Derby[b]				
Derriford, Plymouth	52	43	1.01 (0.79, 1.30)	
Homerton, London	58.5	135	1.08 (0.75, 1.54)	
Liverpool Women's	105.5	123	1.05 (0.78, 1.42)	
North Staffordshire	72.5	132	1.16 (0.82, 1.63)	
Northwick Park, London	45	58	1.05 (0.72, 1.54)	
Nottingham City & Queen's Medical Centre	84.5	64.5	1.02 (0.71, 1.48)	0.50
Princess Anne, Southampton	283	123	0.80 (0.52, 1.24)	
Princess Royal & Southern General, Glasgow	84	111	1.07 (0.84, 1.37)	
Queen Alexandra, Portsmouth	67	49	0.79 (0.51, 1.21)	
Rotunda, Dublin	31	21	0.78 (0.59, 1.04)	
Royal Bolton	24	55	0.90 (0.50, 1.62)	
St. Mary's, Manchester	55	74.5	1.13 (0.83, 1.54)	
Stoke Mandeville	97	134	1.17 (0.85, 1.61)	
UCLH	50	19	0.87 (0.41, 1.81)	
Warrington	35	38.5	0.93 (0.75, 1.14)	
Warwick[b]				
**Total** (unadjusted)	58	58	0.99 (0.92, 1.07)	

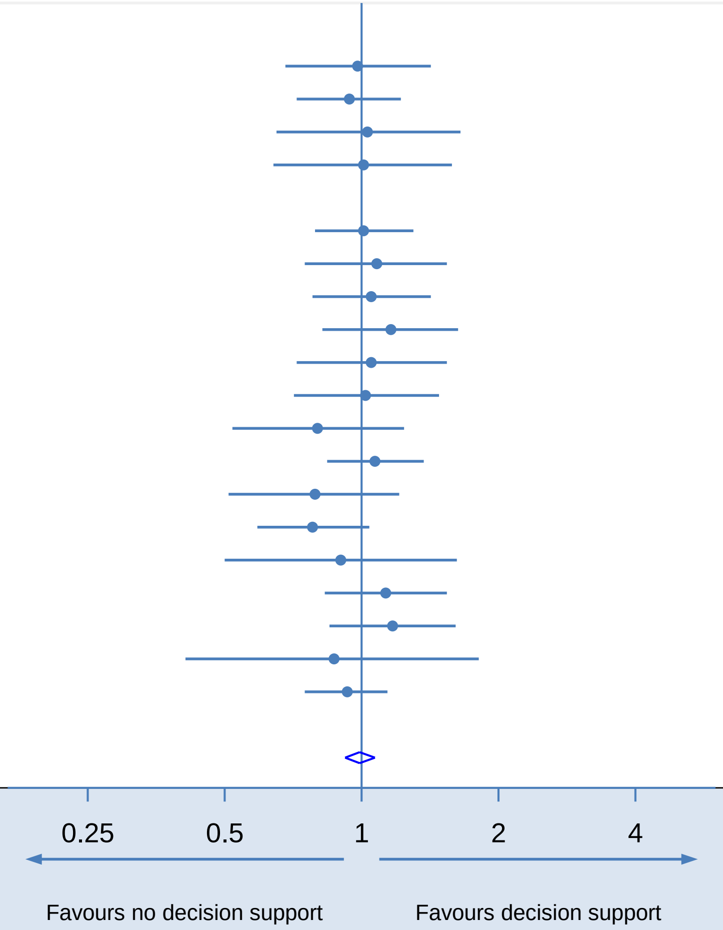

[a] Hazard ratio >1 favours decision support as this implies that time to delivery (from last red level of concern) was reached more quickly compared to no decision support
[b] Data on timing of red level of concerns not available for 2 centres: Warwick (n=823) and Derby (n=832)

Figure S27: Histograms of cord artery pH by trial allocation

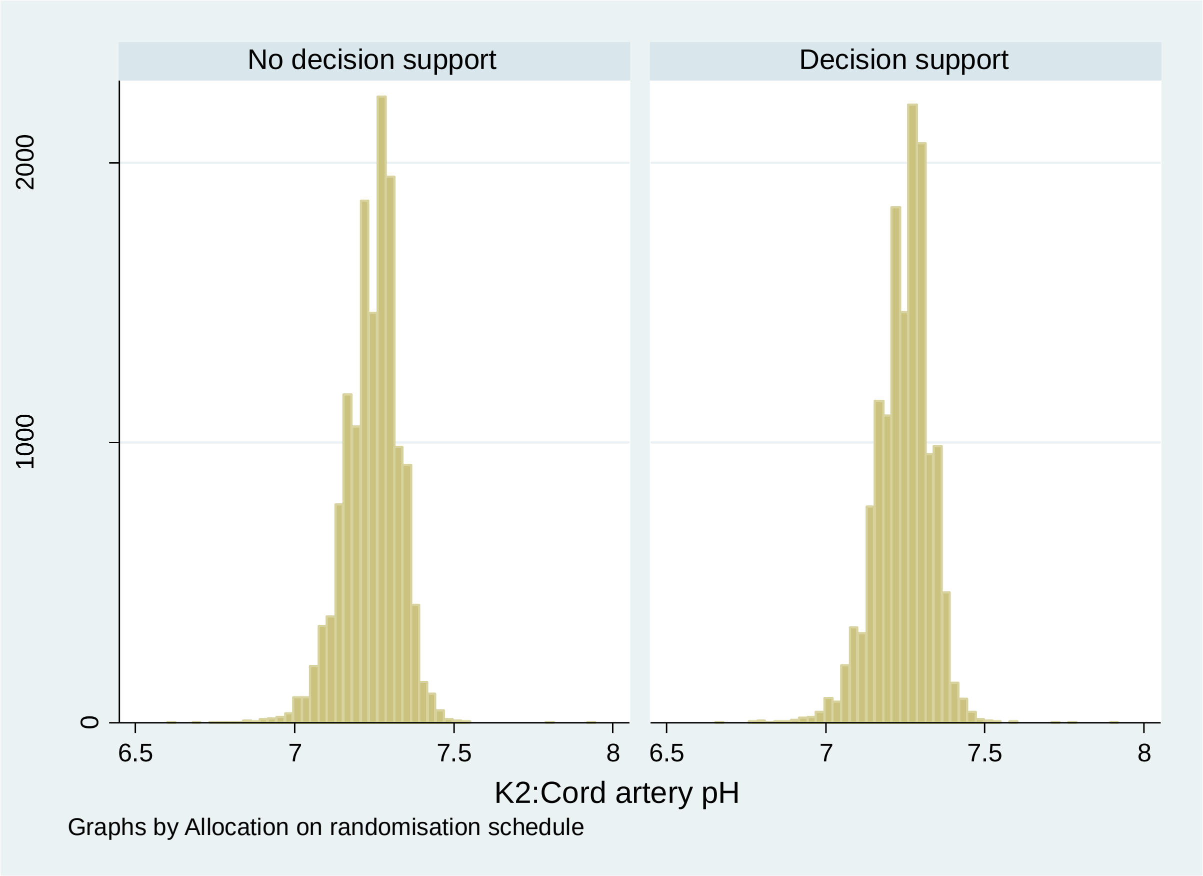

Subgroup analyses

Table S5: PARCA-R Composite score by twin pregnancy

	n	(%)	Decision support Mean [SD]		No decision support Mean [SD]		Mean difference (95% CI)	
Singleton	6,555	(98)	98.2	[33.7]	97.4	[33.4]	0.82	(-0.81 to 2.44)
Twin	152	(2)	85.7	[37.3]	89.7	[32.4]	-3.95	(-19.4 to 11.5)
**Total** (unadjusted)	6,707	(100)	98.0	[33.8]	97.2	[33.4]	0.73	(-0.88 to 2.34)
Unknown	*359*							

P value from the test of interaction = 0.54

Table S6: PARCA-R Composite score by suspected fetal growth restriction (FGR)

	n	(%)	Decision support Mean [SD]		No decision support Mean [SD]		Mean difference (95% CI)	
No FGR	6,472	(96)	98.3	[33.7]	97.5	[33.4]	0.77	(-0.89 to 2.42)
FGR	235	(4)	89.4	[35.0]	90.9	[34.1]	-1.50	(-10.5 to 7.54)
**Total** (unadjusted)	6,707	(100)	98.0	[33.8]	97.2	[33.4]	0.73	(-0.88 to 2.34)
Unknown	*359*							

P value from the test of interaction = 0.63

Table S7: PARCA-R Composite score by body mass index

	n	(%)	Decision support Mean [SD]		No decision support Mean [SD]		Mean difference (95% CI)	
12 to 18.4	104	(2)	94.6	[37.5]	99.9	[37.9]	-5.27	(-20.0 to 9.41)
18.5 to 24.9	2,039	(30)	98.2	[33.7]	97.4	[32.8]	0.86	(-2.07 to 3.78)
25 to 29.9	1,508	(23)	98.3	[34.8]	98.6	[33.3]	-0.34	(-3.82 to 3.14)
30 to 70	1,157	(17)	96.7	[34.8]	95.3	[33.1]	1.43	(-2.53 to 5.39)
Unrecorded	1,899	(28)	98.3	[32.4]	97.1	[34.0]	1.27	(-1.76 to 4.30)
**Total** (unadjusted)	6,707	(100)	98.0	[33.8]	97.2	[33.4]	0.73	(-0.88 to 2.34)
Unknown	*359*							

P value from the test of interaction = 0.86

Table S8: PARCA-R Composite score by centre

Centre[a]	n	(%)	Decision support Mean	[SD]	No decision support Mean	[SD]	Mean difference	(95% CI)
Birmingham Women's	262	(4)	95.1	[32.0]	96.8	[31.0]	-1.74	(-9.54 to 6.06)
Burnley General	832	(12)	94.0	[35.7]	94.0	[33.4]	0.02	(-4.73 to 4.78)
Derriford, Plymouth	814	(12)	95.6	[33.0]	94.7	[34.3]	0.84	(-3.85 to 5.53)
Liverpool Women's	725	(11)	102.4	[33.4]	97.6	[34.6]	4.79	(-0.28 to 9.85)
North Staffordshire	826	(12)	97.1	[34.3]	97.9	[33.3]	-0.75	(-5.37 to 3.88)
Northwick Park, London	146	(2)	86.3	[34.8]	88.4	[31.4]	-2.19	(-13.3 to 8.87)
Nottingham City & Queens Medical Centre	135	(2)	98.1	[33.2]	104.4	[30.0]	-6.30	(-17.1 to 4.50)
Princess Anne, Southampton	205	(3)	99.1	[32.0]	100.9	[32.0]	-1.77	(-10.7 to 7.15)
Princess Royal & Southern General, Glasgow	333	(5)	106.1	[31.9]	105.4	[32.4]	0.69	(-6.27 to 7.65)
Queen Alexandra, Portsmouth	693	(10)	98.2	[33.7]	97.7	[32.4]	0.56	(-4.44 to 5.56)
Rotunda, Dublin	161	(2)	109.8	[32.2]	110.0	[34.9]	-0.19	(-10.7 to 10.3)
St Mary's, Manchester	208	(3)	100.0	[34.8]	96.2	[32.4]	3.73	(-5.73 to 13.2)
Stoke Mandeville	462	(7)	98.3	[32.4]	96.4	[32.0]	1.86	(-4.08 to 7.80)
Warwick	905	(14)	97.4	[33.6]	96.8	(34.4]	0.61	(-3.88 to 5.10)
**Total** (unadjusted)	6,707	(100)	98.0	[33.8]	97.2	[33.4]	0.73	(-0.88 to 2.34)
Unknown	*359*							

P value from the test of interaction = 0.94

[a] 7 centres joining the trial at a later stage did not have infants included in the 2 year follow-up sample.
